# Tail risk, large fluctuations and downfalls in renewable energy markets

**DOI:** 10.1371/journal.pone.0351106

**Published:** 2026-07-15

**Authors:** Akram Shavkatovich Hasanov, Rustam Ibragimov, Ramazan Yildirim, Shwen Kitt Chong, Sirojiddin Abrorov

**Affiliations:** 1 Department of Econometrics and Business Statistics, Monash University Malaysia, Subang Jaya, Malaysia; 2 Imperial College Business School, London, United Kingdom; 3 New Economic School, Moscow, Russia; 4 Upsite Consulting, Muharraq, Kingdom of Bahrain; 5 Financial Market Sector of the Research Centre “Scientific Bases and Issues of the Development of the Economy of Uzbekistan”, Tashkent State University of Economics, Tashkent, Uzbekistan; Incheon National University, KOREA, REPUBLIC OF

## Abstract

The renewable energy sector has expanded rapidly as countries pursue decarbonization goals, increasing investor exposure to market risk and extreme price movements. While prior research has examined downside risk, volatility, and dependence structures in clean energy markets, less is known about the statistical properties governing the decay of extreme returns and the existence of finite moments. This study addresses this gap by analyzing the heavy-tailed behavior of renewable and conventional energy equity indices using daily data from April 2005 to February 2025. We estimate tail indices to assess the degree of heavy-tailedness and to infer the existence of finite moments, relying on confidence-interval-based inference rather than point estimates. In addition to full-sample analysis, a recursive expanding-window approach is employed to examine the time variation of tail risk and its response to major market stress events. The results indicate that both renewable and conventional energy indices exhibit heavy-tailed return distributions consistent with power-law behavior. Confidence-interval-based inference supports the existence of finite first and second moments across all indices, while higher-order moments may be infinite, implying limitations for models that rely on skewness or kurtosis. The recursive analysis reveals pronounced increases in tail risk during periods of systemic stress, particularly during the global financial crisis, highlighting the state-dependent nature of extreme risk in energy markets. Overall, the findings emphasize the importance of statistically justified tail analysis for risk measurement, portfolio construction, and stress testing in renewable energy markets, complementing existing studies focused on volatility and dependence.

## 1. Introduction

Climate change, largely driven by carbon dioxide emissions, has become one of the most pressing global challenges. Carbon dioxide emissions have increased sharply since the Industrial Revolution, with more than 36 billion tonnes released into the atmosphere annually [1]. The consequences of climate change are increasingly visible through more frequent extreme weather events, environmental degradation, and growing pressure on natural and economic systems [2–6]. In response, governments across the globe have intensified efforts to mitigate climate change through decarbonisation strategies, policy interventions, and financing initiatives designed to accelerate the transition from conventional energy sources to clean and renewable energy and to improve energy efficiency [7].

Alongside these developments, interest in renewable energy markets has grown substantially. Increased investment flows into renewable energy have led to a marked expansion in the size and market capitalization of renewable energy firms. Among the available financing channels, equity markets play a central role in supporting renewable energy projects [8]. Prior studies show that the development of renewable energy stock markets facilitates access to capital, enhances liquidity, and supports higher investment in clean and sustainable energy projects [9–11]. Consequently, the stability and risk characteristics of renewable energy equity markets have become increasingly important not only for portfolio allocation decisions, but also for the broader financing conditions underpinning long-term energy-transition objectives and infrastructure deployment. In addition, renewable energy equity markets are influenced by a range of economic and financial factors, including oil price movements, technology sector dynamics, policy uncertainty, and aggregate macroeconomic shocks [12,13]. The interaction of these factors, combined with heightened investor participation, can generate large price movements and increase the likelihood of sharp changes and downfalls in renewable energy stock prices and returns.

Against this background of rapid market growth and increased exposure to market-wide shocks, accurate assessment of extreme fluctuations in renewable energy equity returns becomes essential for managing the risks associated with renewable energy investment portfolios. It is widely acknowledged that financial asset returns deviate from the Gaussian distribution and frequently exhibit heavy-tailed behavior [14–21]. Heavy-tailedness implies that extreme outcomes occur with higher probability than predicted by thin-tailed distributions, which has important consequences for risk measurement, statistical inference, and the reliability of many models used in finance and economics. In the context of renewable energy finance, inaccurate assessment of tail risk may contribute to the mispricing of extreme downside exposures, potentially affecting investment decisions, capital allocation, and the perceived riskiness of renewable energy assets during periods of systemic stress.

Renewable energy equity indices are often reported to exhibit higher volatility than conventional energy benchmarks. For example, the WilderHill New Energy Global Innovation Index recorded annual volatility of approximately 35% between 2005 and 2016, compared with about 17% for a conventional energy index over the same period [22]. While such evidence highlights differences in volatility, dispersion alone does not fully characterize the likelihood or severity of extreme return realizations. Volatility captures average variability around the mean, whereas tail behavior reflects the probability structure governing extreme outcomes. Accordingly, differences in volatility do not necessarily imply statistically meaningful differences in heavy-tailedness or tail risk. The focus of this study is therefore on the unconditional tail behavior of return distributions rather than conditional variance dynamics.

Careful examination of heavy-tailedness is particularly important because sufficiently strong tail behavior can undermine standard conclusions of widely used models in economics, finance, and risk management, including results related to diversification optimality and the conditions under which commonly applied risk measures such as value at risk (VaR) and expected shortfall (ES) remain coherent [21,23]. Moreover, if higher-order moments are unstable or potentially non-finite, statistical inference and portfolio optimization procedures relying on higher moments may become unreliable, particularly during episodes of severe market stress. Reliable analysis of heavy-tailedness can improve the robustness of risk management tools and support more informed decision-making by investors, policymakers, and financial regulators.

A substantial body of literature has investigated tail risk and extreme behavior in financial and commodity markets, including extensive work on cryptocurrency markets [14,21,24–29]. In the context of renewable energy markets, several studies have examined extreme dependence, downside risk, and volatility spillovers using methods such as copulas, CoVaR, and wavelet-based approaches. More recent contributions incorporate global economic and financial events, including geopolitical developments, and analyze their effects across sectoral equity markets, sustainable investments, and clean and conventional energy assets [30–34]. While these studies provide important insights into tail dependence and downside risk, relatively few contributions focus directly on tail index estimation and the associated inference on the existence and order of finite moments in renewable energy equity returns. As a result, the statistical properties governing the decay of extreme returns and the associated inference on tail indices and moment existence in renewable energy markets remain less systematically documented.

This study contributes to the literature in several ways. First, it provides systematic evidence on the degree of heavy-tailedness in renewable energy equity returns using confidence interval (CI)-based tail index inference, allowing direct assessment of the existence and order of finite moments. This perspective differs from much of the existing literature, which primarily emphasizes volatility dynamics, downside risk measures, and dependence structures rather than the unconditional decay of extreme returns. Second, by applying log-log rank-size regression (LLRS) with an optimal rank shift and valid standard errors (SE), the study employs a robust and statistically justified framework for tail index inference in renewable energy markets. Third, the analysis compares renewable energy equity indices with a conventional energy benchmark, offering insights into similarities and differences in tail behavior across energy sectors based on statistically justified inference rather than point estimates alone. In this respect, the study contributes to a better understanding of how extreme market risk may influence the stability and resilience of renewable energy investment environments during periods of systemic stress. Together, these contributions provide a clearer characterization of extreme return behavior in renewable energy equity markets and complement existing evidence on risk and dependence structures.

Consistent with these contributions, the present study conducts a detailed analysis of heavy-tailedness in renewable energy equity returns using tail index estimation methods grounded in extreme value theory (EVT). The analysis is based on five major energy-related equity indices: ECO, SPGCE, ERIX, and SUN, representing renewable energy markets, and DJUSEN as a conventional energy sector benchmark. The data consist of daily historical observations obtained from the Bloomberg Terminal, covering the period from 1 April 2005–25 February 2025. The sample period includes major episodes of global financial and economic stress, most notably the 2007−2008 Global Financial Crisis (GFC) and the COVID-19 pandemic, allowing assessment of tail-risk dynamics under distinct crisis environments.

In addition to full-sample analysis, the study examines the evolution of tail behavior over time using a recursive window approach, following earlier applications in the literature [35,36]. Recursive estimation allows the assessment of how extreme events influence tail behavior as new observations enter the sample. A notable feature of this approach is that once extreme realizations occur, they persist in subsequent windows unless additional extreme observations are recorded as the sample expands. This approach is particularly useful for evaluating the persistence of crisis-related tail risk and the extent to which major systemic shocks continue to influence estimated tail behavior over time.

Several methods have been proposed to estimate the degree of heavy-tailedness in return distributions, among which Hill’s estimator and ordinary least squares (OLS) estimation based on LLRS regressions are among the most widely applied [15,21,37]. Previous studies show that inference based on Hill’s estimator may be sensitive to deviations from power-law behavior, dependence structures, and finite-sample effects [15]. To address these concerns, the present study employs LLRS regression with the optimal rank shift of −1⁄2 and valid SE, which has been shown to substantially reduce finite-sample bias and perform well under dependence structures commonly observed in financial returns, including GARCH-type processes [38]. This modified LLRS framework has been applied extensively in empirical studies across financial and commodity markets [39–43].

The primary objectives of this study are twofold. First, it examines the heavy-tailedness properties of major renewable energy equity indices by estimating tail indices and conducting inference based on CIs to evaluate the existence of finite moments. Second, it applies robust and statistically justified econometric methods to assess the implications of heavy-tailed behavior for tail risk measurement and model reliability in renewable energy markets. Accurate tail index estimates can inform the evaluation of tail-based risk measures such as VaR and ES, as well as the robustness of conclusions drawn from models used in economics, finance, and insurance [21,23]. More broadly, improved understanding of extremal risk in renewable energy markets may contribute to more informed risk assessment, portfolio construction, and stress-testing practices in periods of heightened market uncertainty. This study may also be of interest to financial institutions, investors, risk managers, and policymakers concerned with the tail risk and heavy-tailedness properties of renewable energy equity returns.

The remainder of the paper is organized as follows. Section 2 reviews the relevant literature. Section 3 presents the data and methodological framework. Section 4 reports the empirical results, followed by their discussion in Section 5. Section 6 concludes.

## 2. Literature review

### 2.1 Renewable energy markets

The renewable energy market is expected to expand further as global commitments to mitigate climate change intensify. This has contributed to the growth of the clean energy finance literature, which examines renewable energy markets in relation to financial, economic, technological, and policy-related factors. Existing studies investigate renewable energy assets alongside technology stocks, crude oil prices, broad equity markets, and macroeconomic conditions to better understand the drivers and risk characteristics of renewable energy market dynamics.

A substantial strand of the literature examines the interrelationships between renewable energy markets, conventional energy markets, and technology stocks. Existing studies document significant dependence, causality, spillovers, and transmission mechanisms across these markets [10,44–49]. Empirical evidence suggests that renewable energy equity returns are influenced by both oil price dynamics and technology sector performance, while nonlinear and tail-dependent relationships are also widely documented through copula-based and multi-horizon analyses [46,47]. Additional studies report substantial spillover effects between clean energy, technology, crude oil, and broader equity markets, highlighting the strong interconnectedness of renewable energy assets with global financial and energy systems [48,49].

Another important body of research focuses on the political, economic, and financial risks associated with renewable energy investments. Existing evidence suggests that renewable energy projects are particularly sensitive to financing conditions, policy uncertainty, and market-related risks due to their capital-intensive and innovation-driven nature [50–53]. While some studies report that socially responsible and renewable energy investments do not significantly underperform conventional benchmarks [50], others argue that renewable energy assets may offer relatively modest risk-adjusted returns [51]. Additional evidence identifies policy design risk and financing constraints as important barriers to renewable energy deployment, particularly during the early stages of investment [52,53].

Uncertainty arising from broader economic and financial conditions has also been shown to affect renewable energy markets. Using conditional VaR-based frameworks, some studies find that uncertainty originating from financial and oil markets exerts a stronger influence on energy stock markets than policy uncertainty, while policy uncertainty itself appears more pronounced in renewable energy markets than in traditional energy markets [54]. Additional research documents bidirectional causal relationships between geopolitical risk and renewable energy markets, highlighting the sensitivity of clean energy investments to global political developments [55].

More recent contributions examine heavy-tailedness and tail risk properties of stock returns across different markets, with particular emphasis on the role of financial crises. [36] analyze excess volatility and tail behavior in developed and emerging stock markets and show that, while developed markets often conform to the Cubic Law of Stock Returns, emerging markets exhibit heavier tails. Importantly, after the 2008 financial crisis, both developed and emerging markets experienced a decline in their tail indices, indicating more pronounced heavy-tailedness. Focusing on the Chinese A- and H-share markets, [56] compare tail risk properties under different institutional environments and find that tail indices are similar across markets, with some returns potentially exhibiting infinite second moments. Further analysis of the 2015 Chinese stock market crash reveals structural breaks in heavy-tailedness and identifies liquidity and firm size as key determinants of tail risk, while also showing that tail properties did not revert immediately after the crisis [57].

Traditional asset pricing frameworks such as the Capital Asset Pricing Model (CAPM) and multifactor models have also been applied to renewable energy equities to assess their risk-return characteristics [58]. Empirical findings remain mixed. Some studies report relatively strong risk-adjusted performance and evidence of a green equity premium [59,60], whereas others document periods of weak or negative returns [51,59]. These mixed results underscore the heterogeneity of performance outcomes in renewable energy markets and suggest that renewable energy assets cannot be characterized uniformly from a risk-return perspective.

Against this background, the existing literature provides important insights into the drivers, interdependencies, and risk characteristics of renewable energy markets. At the same time, differences in geographic coverage, sectoral composition, and index construction across studies raise questions regarding the comparability of empirical findings. These issues are addressed in the following subsection, which reviews the renewable energy indices commonly employed in empirical research.

### 2.2 Renewable energy indices

A range of renewable energy equity indices has been employed in the literature to analyze clean energy markets. These indices differ in geographic coverage, sectoral composition, and weighting methodology, which may affect their comparability and the interpretation of empirical findings, particularly in the context of distributional properties and extreme market behavior.

One of the most widely used renewable energy indices is the WilderHill Clean Energy Index (ECO). This equal-dollar-weighted index tracks publicly traded U.S. firms operating in clean energy sectors such as wind, ethanol, solar, and hydrogen fuel cells [61]. As one of the earliest indices designed to capture clean energy and climate-related solutions, ECO remains an important benchmark in the literature and has been employed in studies examining renewable energy market dynamics, uncertainty transmission, causality, and volatility behaviour [62–64]. Owing to its U.S.-centric composition, ECO primarily reflects developments in the North American clean energy market.

The S&P Global Clean Energy Select Index (SPGCE) provides broader international exposure by tracking firms engaged in clean energy-related activities across developed and emerging markets [65]. Its global scope has motivated its use in studies examining dependence structures, safe-haven properties, and interactions between clean energy and commodity markets [66–68]. The inclusion of firms from emerging economies may expose SPGCE to additional sources of regulatory, geopolitical, and market-related risk, potentially influencing its tail behavior relative to regionally concentrated indices.

The European Renewable Energy Index (ERIX) focuses on renewable energy firms operating within Europe, including companies active in wind, biomass, marine, water, and geothermal energy. ERIX has been used to examine the effects of non-renewable energy shocks, carbon risk management, and renewable energy market dynamics within the European institutional environment [69,70]. Its regional focus reflects regulatory and market conditions specific to European energy markets, which may shape both return dynamics and extremal risk characteristics.

In contrast to the broader clean energy indices discussed above, the MAC Global Solar Energy Index (SUN) focuses specifically on firms operating in the solar energy sector, including equipment manufacturers and suppliers of services and materials to solar technology producers. This index has been employed in several empirical studies of renewable energy markets [66,71–73]. Due to its sector-specific composition, SUN may be particularly sensitive to technology cycles, supply-chain disruptions, and policy interventions, potentially generating tail behavior distinct from that of more diversified clean energy indices.

To facilitate comparison with conventional energy markets, the present study also considers the Dow Jones U.S. Energy Index (DJUSEN) as a benchmark. DJUSEN tracks large U.S.-based firms engaged in traditional energy activities, including oil and gas exploration, production, and related services. Due to its U.S.-centric composition and focus on conventional energy production, DJUSEN reflects exposure to different regulatory frameworks, commodity price dynamics, and market structures than renewable energy indices, making it a suitable benchmark for assessing differences in tail behavior between clean and conventional energy markets [74].

The indices considered in this study differ materially in terms of geographic exposure, sectoral focus, and weighting methodology. ECO is U.S.-focused and equal-weighted, SPGCE represents a global renewable energy universe and is market-capitalization weighted, SUN is sector-specific with a focus on solar energy firms, and DJUSEN serves as a conventional U.S. oil and gas benchmark. This heterogeneity enhances the external relevance of the analysis by capturing alternative market representations of energy assets. Differences in weighting methodology may also influence the degree of exposure to smaller and potentially more volatile firms, which could affect observed tail behavior across indices. At the same time, it limits direct causal attribution of cross-index differences in tail behavior to any single index characteristic.

### 2.3 Heavy-tailedness and inference

Heavy-tailed distributions refer to probability distributions whose tails are not exponentially bounded, in contrast to thin-tailed distributions such as the normal distribution [75]. Compared with thin-tailed distributions, heavy-tailed distributions exhibit slower tail decay and therefore assign relatively higher probability to extreme realizations [21]. The presence of such extremes affects sample statistics and their asymptotic properties, often resulting in inflated estimates of higher-order moments. Examples include Pareto, Student-t, stable, and other power-law distributions [15,21,23,76].

A broad empirical literature across economics, finance, insurance, risk management, and related disciplines documents that many variables of interest, including financial returns and exchange rates, deviate from Gaussianity and exhibit heavy-tailed behavior, often in the form of power-law tails [15–21,23,76]. Early contributions by [77] and [78] established the presence of heavy-tailed characteristics in financial returns and commodity prices, motivating a large subsequent literature. Later theoretical and empirical studies, particularly [18,79,80], link the emergence of power-law tails to economic mechanisms associated with the Zipf distribution of firm and investor sizes and their trading impact. Heavy-tailedness therefore plays a central role in assessing the likelihood of extreme market fluctuations and is commonly quantified through tail indices.

The degree of heavy-tailedness is measured by tail indices, which characterize both (i) the rate of power-law decay of distributional tails and (ii) the highest order of finite moments of the underlying stochastic variable [21]. For a return or economic variable r (e.g., a measure of risk) that follows a heavy-tailed power-law distribution, the tail behavior can be expressed as follows:


$$\begin{array}{c} P(r>x)\;\sim \;\frac{M_{\text{1}}}{x^{\zeta^{+}}} \end{array}$$ (1)

$$\begin{array}{c} P(r<-x)\;\sim \;\frac{M_{\text{2}}}{x^{\zeta^{-}}} \end{array}$$ (2)

where $\zeta^{+},\zeta^{-}>\text{0},\;M_{\text{1}},M_{\text{2}}>\text{0}$, and x→+∞. Defining $\zeta =\text{min}(\zeta^{+},\zeta^{-})$, one obtains


$$\begin{array}{c} P\left(|r|>x\right)\;\sim \;\frac{M}{x^{\zeta }} \end{array}$$ (3)

with M>0 as x→+∞. The parameters ζ, $\zeta^{+}$, and $\zeta^{-}$ are referred to as the tail index and the right and left tail indices, respectively. Smaller values of ζ imply heavier tails, slower decay of extreme probabilities, and a lower order of finite moments. Consequently, lower tail index values correspond to a higher likelihood of observing extreme outcomes [21].

The tail index directly determines moment existence. If ζ>1, the mean is finite; if ζ>2, the variance is finite; and if ζ>4, the fourth moment and kurtosis are finite. Empirical evidence from financial and commodity markets indicates that tail indices for stock returns and exchange rates in developed markets often lie in the range of 2<ζ<4, implying finite means and variances but infinite fourth moments [18–20,24,80]. At the same time, returns in both developed and emerging markets may exhibit tail indices below 2, corresponding to infinite variances [21,25,26,56,57]. Related evidence from wealth and income distributions suggests tail index values around ζ≈1.5 for wealth and ζ∈(1.5,3) for income, indicating potentially infinite variances and higher moments [19–21].

The literature proposes several approaches for estimating tail indices of heavy-tailed distributions, with comprehensive reviews provided in [15,21,81]. Two of the most widely applied methods are (i) Hill’s estimator [82], also known as a quasi-maximum likelihood estimator, and (ii) OLS estimation based on LLRS regressions [21,38]. Although alternative and more complex estimation techniques exist, Hill’s estimator and LLRS regressions remain popular due to their relative simplicity and widespread applicability in empirical work.

An important practical consideration in tail index estimation concerns the choice of the tail threshold or truncation level. Diagnostic tools such as mean excess plots and the stability of Hill estimates across alternative threshold choices are commonly discussed in the EVT literature. At the same time, many empirical studies adopt fixed tail fractions, such as 5% or 10%, to facilitate comparability across assets and to mitigate small-sample instability in financial return data. Consistent with this established practice, the present study employs fixed truncation levels while emphasizing statistical inference based on CI rather than reliance on point estimates alone. Lower truncation levels may capture more extreme observations but can also increase estimation uncertainty due to the smaller effective number of tail observations, particularly in finite samples.

In empirical applications, observed tail behavior may deviate from an exact Pareto specification due to slowly varying components and nonlinear dependence structures typical of financial time series. Importantly, LLRS regression with the optimal rank shift and corrected SE has been shown to exhibit favorable finite-sample performance and robustness under such deviations, including in the presence of volatility clustering and GARCH-type dynamics, as documented in [38]. In this setting, tail index estimation is interpreted as characterizing effective tail thickness and moment implications rather than testing a literal Pareto data-generating process.

Overall, statistically justified tail index estimation is essential, as inappropriate modeling of tail behavior may lead to misleading inference and economic conclusions. These considerations motivate the emphasis on robust inference procedures and CI-based interpretation when assessing heavy-tailedness and extremal risk in financial markets.

## 3. Methodology and data

This study employs two complementary approaches to estimate the tail indices of renewable energy stock returns, as discussed in the previous section. Specifically, we apply Hill’s estimator and the OLS LLRS regression approach with the optimal rank shift parameter γ =1/2 and corrected SE, following [21,38]. Hill’s estimator is used as a benchmark method that is widely adopted in the literature on heavy-tailed financial returns, while the LLRS regression with optimal shift constitutes the primary estimation framework of the analysis due to its favorable finite-sample properties and robustness to dependence structures commonly observed in financial data.

### 3.1 Hill’s estimator

Hill’s estimator is a semi-parametric method for estimating the tail index of a heavy-tailed distribution. It approximates the upper tail of the return distribution by a Pareto law and estimates the corresponding tail index using a maximum likelihood principle. Owing to its conceptual simplicity and long-standing use in empirical finance, Hill’s estimator provides a natural benchmark for assessing the degree of tail risk in asset returns.

Consider a sample $r_{\text{1}},\;r_{\text{2}},\;\ldots ,\;r_{N}$ drawn from a population whose tail behavior follows a power-law distribution as defined in Eqs. (1)-(3) with tail ζ. Let n<N and define the ordered absolute returns as:


$$\begin{array}{c} |r{|}_{\left(\text{1}\right)}\geq |r{|}_{\left(\text{2}\right)}\geq \;\ldots \geq |r{|}_{\left(n\right)}\geq |r{|}_{\left(n+\text{1}\right)} \end{array}$$ (4)

where $|r{|}_{\left(j\right)}$ denotes the j-th largest absolute return. In empirical applications, the truncation level n is typically selected as a fixed fraction of the total sample size, such as 5% or 10% of N. This choice reflects a trade-off between bias and variance in tail index estimation. Lower truncation levels focus more directly on the most extreme observations but may also increase estimation uncertainty due to the smaller effective number of tail observations, particularly in finite samples. This effect is typically reflected in wider CIs at lower truncation levels. Further details on truncation rules are provided in [21,25].

Under these conditions, Hill’s estimator ${\hat{\zeta }}_{Hill}$ of the tail index ζ associated with the absolute-tail power-law model in Eq. (3) is given by:


$$\begin{array}{c} {\hat{\zeta }}_{Hill}=\frac{n}{\sum_{t=\text{1}}^{n}\left(log|r{|}_{\left(t\right)}-log|r{|}_{\left(n+\text{1}\right)}\right)} \end{array}$$ (5)

The corresponding SE is:


$$\begin{array}{c} {SE}_{Hill}=\frac{\text{1}}{\sqrt{n}}{\hat{\zeta }}_{Hill} \end{array}$$ (6)

which yields the following 95% CI for ζ:


$$\begin{array}{c} \text{9}\text{5}\%\;{CI}_{Hill}=\left[{\hat{\zeta }}_{Hill}-\frac{\text{1}.\text{9}\text{6}}{\sqrt{n}}{\hat{\zeta }}_{Hill},\;{\hat{\zeta }}_{Hill}+\frac{\text{1}.\text{9}\text{6}}{\sqrt{n}}{\hat{\zeta }}_{Hill}\right] \end{array}$$ (7)

Hill’s estimator possesses desirable efficiency properties under the assumption of independent and identically distributed (i.i.d.) observations drawn from an exact power-law distribution. In such settings, it coincides with the maximum likelihood estimator (MLE) and exhibits standard asymptotic behavior [21,83]. However, empirical studies have shown that Hill-based inference is sensitive to finite samples, heterogeneous observations, and serial dependence, all of which may lead to biased tail index estimates and understated SE. These limitations are particularly relevant in financial applications, where return series exhibit volatility clustering and temporal dependence in extreme observations, violating the i.i.d. assumption [15,19,21,38,83]. Illustrations of poor finite-sample performance under dependence and deviations from exact power-law behavior are provided by the so-called Hill horror plots in Chapters 4–6 of [15].

To address these shortcomings, several alternative tail index estimators have been proposed, including weighted variants of Hill’s estimator [84] and more sophisticated nonlinear estimation procedures [15,37]. Despite these developments, the LLRS regression framework remains one of the most widely used approaches for tail index estimation in empirical research. Its continued popularity reflects both its analytical simplicity and its robustness properties when appropriately implemented. In particular, LLRS regressions with an optimal rank shift and corrected SE have been shown to perform well in settings characterized by dependence and deviations from idealized power-law assumptions, as documented in [38] and discussed further in [21].

### 3.2 OLS LLRS regression

While Hill’s estimator provides a widely used benchmark for tail index estimation, an alternative and equally prominent approach is based on LLRS regressions. This method exploits the linear relationship implied by power-law tails between the logarithm of an observation’s rank and the logarithm of its magnitude and has been extensively employed in empirical studies of heavy-tailed financial and economic variables due to its simplicity and flexibility.

To estimate the tail index ζ using the conventional LLRS approach, one typically considers the following OLS regression with shift parameter γ = 0, as discussed in [21,38]:


$$\begin{array}{c} og(t-\gamma )=a-b\;log|r{|}_{\left(t\right)},\;t=\text{1},\;...,\;n \end{array}$$ (8)

where t denotes the rank of the ordered absolute return $|r{|}_{\left(t\right)}$, defined as in Eq. (4), and n is the tail truncation level. Equivalently, interpreting t as the rank and $|r{|}_{\left(t\right)}$ as the size of the observation, the estimated regression can be written as:


$$\begin{array}{c} og(rank-\gamma )=a-b\;log(size) \end{array}$$ (9)

Under the power-law model for absolute returns given in Eq. (3), the slope coefficient $\hat{b}$ obtained from Eqs. (8) or (9) provides an estimate of the tail index ζ. As with Hill’s estimator, the LLRS framework can be applied separately to positive and negative returns in order to estimate the right and left tail indices $\zeta^{+}$ and $\zeta^{-}$ defined in Eqs. (1) and (2), respectively.

The LLRS regression approach to tail index estimation has been widely adopted in the empirical literature on heavy-tailed phenomena, including applications in finance, insurance, and macroeconomic risk analysis. Representative examples include [85–87], among many others. Its appeal stems from its transparent interpretation, ease of implementation, and direct link to the underlying power-law structure of tail distributions.

Despite these advantages, it is well known that the standard LLRS estimator based on γ=0 may suffer from substantial finite-sample bias, particularly when the number of tail observations is limited. This limitation motivates refinements of the LLRS framework that improve its small-sample performance and robustness properties.

### 3.3 OLS LLRS regression with optimal shift γ =1/2

As established in [38], the OLS estimator $\hat{b}$ obtained from the standard LLRS regression in Eq. (8) with γ=0 is consistent for the tail index ζ under idealized conditions. However, this estimator exhibits pronounced finite-sample bias, which is particularly relevant in empirical applications where the effective number of tail observations is limited. To address this limitation, [38] propose a simple yet effective correction based on introducing an optimal rank shift of γ=1/2 into the LLRS regression.

Specifically, the modified LLRS approach consists of estimating the following OLS regression:


$$\begin{array}{c} og(t-\text{1}/\text{2})=a-b\;log|r{|}_{\left(t\right)},\;t=\text{1},\;\ldots ,\;n \end{array}$$ (10)

As in the standard LLRS framework, the slope coefficient $\hat{b}$ from Eq. (10) provides an estimate of the tail index ζ. Throughout the paper, the resulting bias-corrected estimator is denoted by ${\hat{\zeta }}_{RS}$.

The rank-shift of 1/2 (implemented as log(t−1/2)) is optimal in the sense that it eliminates the leading-order finite-sample bias of the LLRS estimator. Moreover, [38] derive the correct asymptotic SE for ${\hat{\zeta }}_{RS}$, given by:


$$\begin{array}{c} {SE}_{RS}=\sqrt{\frac{\text{2}}{n}}{\hat{\zeta }}_{RS} \end{array}$$ (11)

Based on these corrected SE, the corresponding 95% CI for the tail index ζ is:


$$\begin{array}{c} \text{9}\text{5}\%\;{CI}_{RS}=\left[{\hat{\zeta }}_{RS}-\text{1}.\text{9}\text{6}\;\times \sqrt{\frac{\text{2}}{n}}{\hat{\zeta }}_{RS},\;{\hat{\zeta }}_{RS}+\text{1}.\text{9}\text{6}\;\times \sqrt{\frac{\text{2}}{n}}{\hat{\zeta }}_{RS}\right] \end{array}$$ (12)

Importantly, the order statistics used in tail index estimation are dependent even when the underlying sample is i.i.d., since the ranking procedure itself induces cross-sectional dependence across observations. For this reason, the asymptotic SEs derived in [38] differs from conventional OLS SE and incorporate the $\sqrt{\text{2}}$ correction in Eq. (11), yielding valid CIs for tail index inference. As demonstrated in their theoretical analysis and numerical evidence, the shifted LLRS estimator with corrected SE remains robust under empirically relevant dependence, including volatility clustering and GARCH-type dynamics.

An important advantage of the LLRS regression with optimal rank shift is its robustness to deviations from exact power-law behavior, including slowly varying components that may arise in empirical tail data. As documented in [38], the estimator exhibits favorable finite-sample performance under such departures from idealized Pareto specifications. These properties make the shifted LLRS framework particularly suitable for empirical tail-risk analysis in financial markets. The LLRS regression with optimal rank shift and corrected SE has subsequently been employed in a wide range of empirical studies, including [25–27,39,56,57,88,89], among others. Owing to its favorable finite-sample properties and theoretical robustness, this approach constitutes the primary tail index estimation method used in the present study.

To complement these theoretical robustness properties, we assess serial dependence and volatility clustering in the return series using Ljung-Box diagnostics (Appendix Table A1 S1 Table), apply GARCH(1,1) filtering to extract standardized return innovations (Appendix Fig A1 S1 Fig), and re-estimate tail indices using both raw and filtered returns as a robustness check (Appendix Table A2 S2 Table). Comparing tail index estimates obtained from raw returns and GARCH-filtered residuals provides additional insight into the sources of heavy-tailedness in renewable energy equity returns. While filtering reduces the influence of volatility clustering and conditional heteroskedasticity, the persistence of heavy-tailed behavior in standardized residuals indicates that extremal risk cannot be attributed solely to time-varying volatility dynamics. The filtered-return analysis is therefore interpreted as a robustness assessment of whether heavy-tailedness primarily reflects conditional volatility effects or broader unconditional distributional properties.

Throughout the paper, ${\hat{\zeta }}_{Hill}$ and ${\hat{\zeta }}_{RS}$ denote the Hill and shifted LLRS estimates of the tail index ζ associated with the power-law model in Eq. (3). The corresponding SE and 95% CI are denoted by ${SE}_{Hill}$, ${SE}_{RS}$, 95% ${CI}_{Hill}$ and 95% ${CI}_{RS}$, as defined in Eqs. (6), (11), (7), and (12), respectively. Estimates of the right and left tail indices are denoted by $\overset{+}{\underset{Hill}{\hat{\zeta }}},\;\overset{-}{\underset{Hill}{\hat{\zeta }}},\;\overset{+}{\underset{RS}{\hat{\zeta }}}$, and $\overset{-}{\underset{RS}{\hat{\zeta }}}$, with analogous notation used for their corresponding SE and CI.

### 3.4 Data

This study analyzes four renewable energy equity indices and one conventional energy index to examine tail risk and heavy-tailedness properties across clean and traditional energy markets. The renewable energy indices considered are the WilderHill Clean Energy Index (ECO), the S&P Global Clean Energy Index (SPGCE), the European Renewable Energy Index (ERIX), and the MAC Global Solar Energy Index (SUN). The Dow Jones U.S. Oil and Gas Index (DJUSEN) is employed as a benchmark representing conventional energy assets. Together, these indices provide coverage across different geographic regions (United States, Europe, and global markets) and sectoral scopes (broad renewable energy versus solar-specific activity), allowing for meaningful cross-index comparisons.

Daily index price data are obtained from the Bloomberg Terminal. The price series cover the period from 1 April 2005–25 February 2025. Daily log returns are computed as $r_{t}=\text{1}\text{0}\text{0}\times \Delta \text{log}\left(P_{t}\right)$ where $P_{t}$ denotes the index price at time t.[All main empirical conclusions remain qualitatively unchanged when arithmetic returns are used instead.] Log returns are commonly used in extreme value and tail-risk analysis and provide a convenient representation for asymptotic tail inference. This transformation ensures scale invariance, facilitates comparability across indices with different price levels, and aligns the empirical framework with standard practice in financial econometrics.

Due to the log-return transformation, the effective return sample begins on 4 April 2005 and ends on 25 February 2025. The number of available observations differs slightly across indices because of exchange-specific trading calendars, public holidays, and index-level data availability. The final sample sizes are 5,008 observations for ECO, 5,176 for SPGCE, 5,112 for ERIX, 5,169 for SUN, and 5,021 for DJUSEN. All data transformations and subsequent empirical analyses were implemented using R.

A summary description of the indices and their defining characteristics is provided in Table 1. These indices are widely employed in empirical studies of renewable and conventional energy markets and offer a suitable basis for examining tail risk and heavy-tailedness across alternative geographic and sectoral representations of energy assets.

**Table 1:** Renewable energy and conventional energy indices.

Index	Abbreviation	Bloomberg Ticker	Definition
**WilderHill Clean Energy Index**	ECO	ECOTR	An equal-dollar-weighted index tracking publicly traded U.S. companies whose business activities significantly contribute to the transition toward cleaner energy and energy conservation, including wind, solar, ethanol, and hydrogen technologies.
**S&P Global Clean Energy Select Index**	SPGCE	SPGTCLEN:IND	A global equity index tracking companies involved in clean energy-related businesses, including equipment manufacturing, production, and enabling technologies, across developed and emerging markets. The index comprises approximately 30 constituents, selected based on liquidity and exposure to clean energy activities.
**European Renewable Energy Index**	ERIX	ERIX:IM	An index measuring the performance of European renewable energy companies engaged in one or more renewable segments, including wind, biomass, geothermal, marine, water, and solar energy.
**MAC Global Solar Energy Index**	SUN	SUNIDX	An index tracking publicly listed companies worldwide that specialize in solar energy products and services, including solar equipment manufacturers and related supply-chain firms.
**Dow Jones U.S. Oil and Gas Index**	DJUSEN	DJUSEN	An index representing large U.S.-based companies operating in the oil and gas sector, including exploration, production, and related services, and serving as a benchmark for conventional energy markets.

Notes: Table 1 reports the renewable and conventional energy equity indices analyzed in this study, including abbreviations, Bloomberg tickers, and index definitions. All data are sourced from the Bloomberg Terminal.

## 4 Results

Due to the well-documented small-sample bias of Hill’s tail index estimator, its sensitivity to dependence in observations, and potential deviations from exact power-law behavior, the empirical analysis and inference on heavy-tailedness in this paper primarily rely on the LLRS regression approach. LLRS offers improved finite-sample performance and robustness under dependence structures while remaining consistent with Pareto-type tail behavior. Nevertheless, for completeness and comparability with the existing literature, Hill-type tail index estimates are also reported. Consistent with prior evidence (e.g., [25]), both approaches yield similar point estimates and lead to qualitatively aligned conclusions regarding tail behavior. Formal inference throughout this section is based on CIs and hypothesis testing principles outlined in Section 2.3.

### 4.1 Return plots and summary statistics

**Fig 1:**
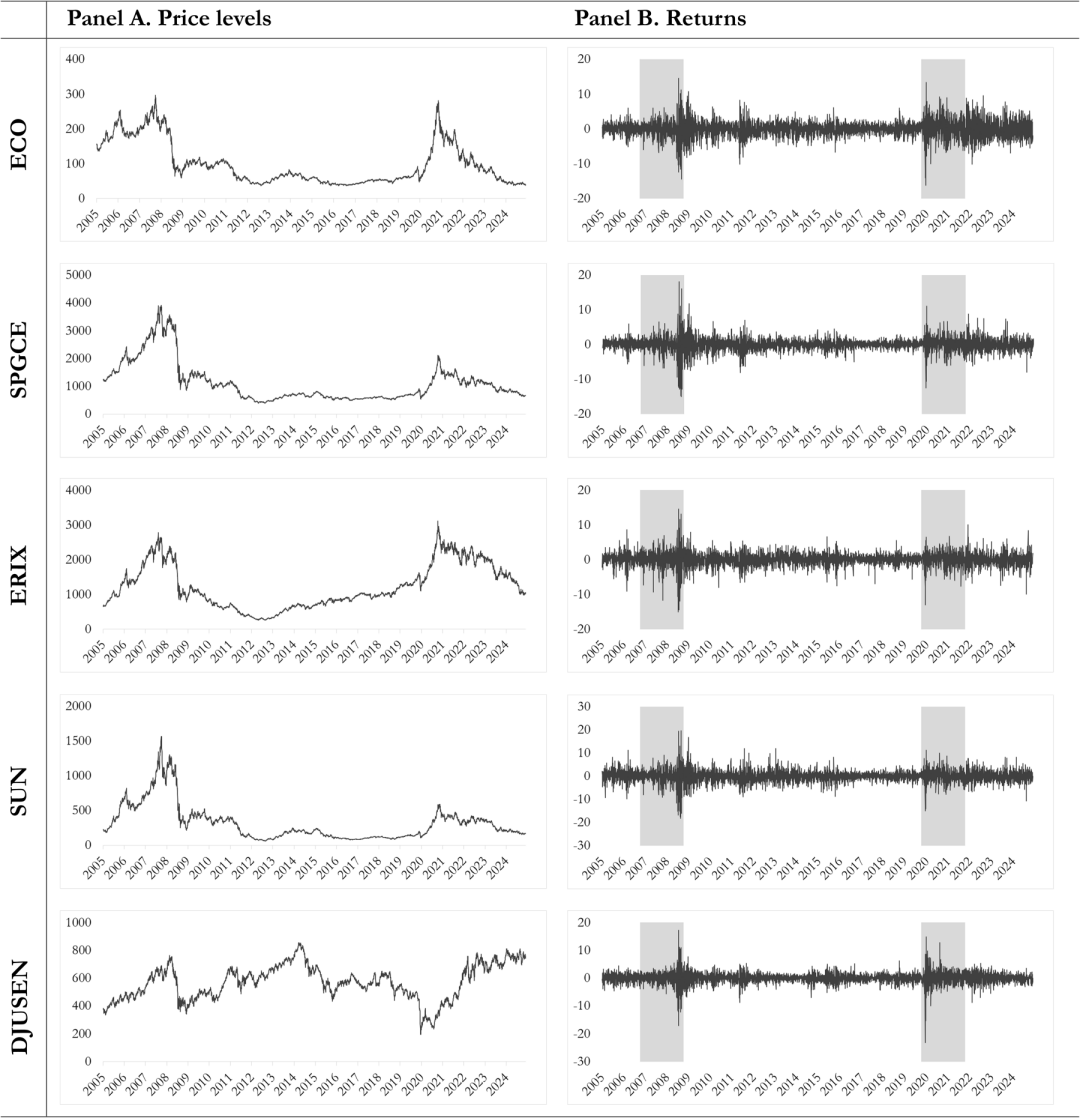
Price and return dynamics of renewable and conventional energy indices. Notes: Panel A shows price levels, and Panel B shows daily log returns for ECO, SPGCE, ERIX, SUN, and DJUSEN over the sample period. Grey shaded areas in Panel B denote periods associated with major global stress events, namely the 2007–2008 global financial crisis (GFC) and the COVID-19 market shock in 2020–2021.

Fig 1 presents the evolution of price levels (Panel A) and daily log returns (Panel B) for renewable energy indices (ECO, SPGCE, ERIX, SUN) and the conventional energy index (DJUSEN) over the full sample period. The price series exhibit pronounced boom-bust dynamics, particularly during major global stress episodes, while the return series display clear volatility clustering and episodic extreme movements. Visual inspection suggests broadly similar volatility patterns across renewable and conventional energy markets, especially during periods of systemic financial stress.

Two major episodes of heightened volatility are apparent as highlighted in Fig 1 Panel B. The first corresponds to the 2007−2008 GFC, during which global financial markets and banking systems experienced severe distress and exceptionally elevated volatility (e.g., [90]). These conditions affected all major asset classes, including renewable and conventional energy markets. The second episode occurs in early 2020 following the outbreak of the COVID-19 pandemic. The unprecedented collapse in economic activity, combined with disruptions in energy demand and financialized commodity trading, culminated in extreme price and return movements, including the historically negative settlement price of West Texas Intermediate (WTI) crude oil futures in April 2020. Existing literature documents strong interdependencies between oil prices and renewable energy markets, implying that extreme return realizations in renewable energy indices during this period are consistent with broader energy-market dynamics (see Section 2.1).

Table 2 reports summary statistics for daily log returns of all indices considered. Average returns are close to zero across all series, which is typical for high-frequency financial data. Among renewable energy indices, SUN exhibits the highest return volatility, as measured by the standard deviation (SD), while SPGCE displays the lowest. Skewness estimates are uniformly negative, indicating a tendency toward larger downside than upside return realizations.

**Table 2:** Summary statistics of daily log returns.

	Mean	Median	Maximum	Minimum	SD	Skewness	Kurtosis	JB
**ECO**	−0.028	0.073	14.519	−16.239	2.285	−0.262	3.991	3385.981***
**SPGCE**	−0.012	0.043	18.093	−14.973	1.853	−0.440	11.048	26516.291***
**ERIX**	0.009	0.077	14.589	−14.990	1.893	−0.411	6.288	8574.697***
**SUN**	−0.006	0.010	19.630	−18.212	2.537	−0.282	6.459	9062.368***
**DJUSEN**	0.013	0.054	17.226	−23.179	1.873	−0.681	13.978	41304.974***

Notes: Table 2 reports descriptive statistics for daily log returns of renewable energy indices (ECO, SPGCE, ERIX, SUN) and the conventional energy index (DJUSEN). JB denotes the Jarque-Bera test for normality. ***, **, and * indicate statistical significance at the 1%, 5%, and 10% levels, respectively.

Kurtosis and Jarque-Bera statistics strongly reject the null hypothesis of normality for all return series at conventional significance levels, confirming substantial departures from Gaussian behavior. These statistics are reported as descriptive diagnostics of non-normality and distributional asymmetry. However, in the presence of volatility clustering, dependence, and potentially infinite higher-order moments, kurtosis is not informative about tail heaviness. Accordingly, tail behavior and moment existence are formally evaluated using tail index estimates and CI-based inference, as reported in Section 4.2.

Augmented Dickey-Fuller (ADF) test results indicate that all price series are non-stationary in levels, while their corresponding return series are stationary across alternative deterministic specifications. These results, consistent with standard financial time-series properties, are reported in Appendix Table A3 S3 Table.

### 4.2 Tail risk and tail index estimates

Building on the descriptive evidence in Section 4.1 and the heavy-tailed inference framework outlined in Section 2.3, this section provides a formal assessment of tail risk in renewable and conventional energy markets using tail index estimation. The analysis focuses on overall tail behavior and potential gain-loss asymmetries, with statistical inference conducted using CI-based methods rather than reliance on point estimates alone.

Table 3 reports tail index estimates obtained using the LLRS regression approach with the optimal rank shift of −1/2, together with corresponding estimates based on Hill’s estimator. Results are presented for two truncation levels, 10% and 5%, retaining the largest 0.10N and 0.05N extreme observations, respectively, from a total sample of size N. These truncation choices are standard in the EVT and financial tail-risk literature and facilitate comparability across assets and studies (see [15,25,56,91,92]). For each estimator and truncation level, standard errors and 95% CIs are reported.

**Table 3:** The tail index estimates for renewable energy and conventional energy indexes.

Index	LLRS regression			Hill’s estimation			N[n]
	${\hat{\zeta }}_{RS}$	${SE}_{RS}$	95% CI [LB, UB]	${\hat{\zeta }}_{Hill}$	${SE}_{Hill}$	95% CI [LB, UB]	
**Part A. Truncated (10%)**							
**ECO**	3.410	0.216	[2.987, 3.833]	2.993	0.134	[2.731, 3.256]	5008 [500]
**SPGCE**	2.626	0.163	[2.305, 2.946]	2.536	0.112	[2.317, 2.755]	5176 [517]
**ERIX**	3.110	0.195	[2.729, 3.492]	2.827	0.125	[2.582, 3.072]	5112 [511]
**SUN**	3.051	0.190	[2.679, 3.424]	2.711	0.119	[2.477, 2.945]	5169 [516]
**DJUSEN**	2.878	0.182	[2.522, 3.234]	2.888	0.129	[2.636, 3.141]	5021 [502]
**Part B. Truncated (5%)**							
**ECO**	3.862	0.345	[3.185, 4.539]	3.411	0.216	[2.988, 3.834]	5008 [250]
**SPGCE**	2.800	0.247	[2.317, 3.283]	2.557	0.159	[2.245, 2.869]	5176 [258]
**ERIX**	3.386	0.300	[2.798, 3.974]	3.108	0.195	[2.727, 3.490]	5112 [255]
**SUN**	3.303	0.291	[2.733, 3.873]	3.052	0.190	[2.680, 3.425]	5169 [258]
**DJUSEN**	2.832	0.253	[2.337, 3.328]	3.052	0.193	[2.675, 3.430]	5021 [251]

Notes: Table 3 reports tail index estimates for renewable energy indices (ECO, SPGCE, ERIX, SUN) and the conventional energy index (DJUSEN) based on daily log returns. ${\hat{\zeta }}_{RS}$ denotes tail index estimates obtained using the LLRS regression approach with optimal rank shift 1/2 (implemented as log(t − 1/2)), while ${\hat{\zeta }}_{Hill}$ denotes estimates based on Hill’s estimator. Reported 95% CIs are shown in square brackets. Results are shown for truncation levels of 10% (Panel A) and 5% (Panel B), where n denotes the number of extreme observations used in estimation and N denotes the total sample size. See Appendix Table A4 S4 Table for a summary of moment existence implications based on the reported CIs.

**Fig 2:**
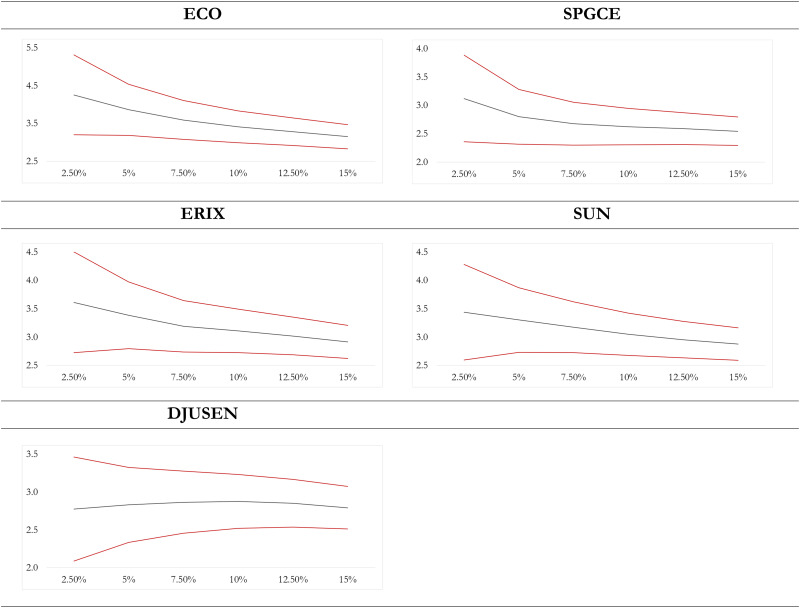
LLRS-based tail index estimates across alternative truncation levels. Notes: Fig 2 displays LLRS-based tail index estimates for renewable energy indices (ECO, SPGCE, ERIX, SUN) and the conventional energy index (DJUSEN) across truncation levels ranging from 2.5% to 15%. Black lines denote point estimates of the tail index, while red lines indicate the corresponding upper and lower 95% CI. Truncation levels represent the fraction of the largest absolute return observations retained in the tail estimation.

To support the choice of truncation levels and assess robustness, Fig 2 presents truncation-stability plots of LLRS-based tail index estimates across a wider range of tail fractions, from 2.5% to 15%, together with their corresponding CIs. CIs widen at lower truncation levels because the effective number of tail observations decreases as estimation focuses more narrowly on the most extreme realizations, thereby increasing estimation uncertainty in finite samples. The estimates exhibit a relatively stable pattern across truncation levels, and the substantial overlap of CIs indicates that differences across thresholds are not statistically distinguishable. This suggests that the inferred power-law tail behavior of renewable energy returns is robust to the choice of truncation level and that the core conclusions regarding the (in)finiteness of moments are not driven by a specific threshold selection. Across this range, the effective number of tail observations varies from approximately 125 to over 750 observations, ensuring that asymptotic approximations are meaningful while avoiding undue contamination from the central part of the return distribution.

Differences in estimated tail indices across renewable energy indices should therefore be interpreted as descriptive evidence of heterogeneous tail risk profiles rather than as outcomes driven by a single underlying factor. While broader geographic exposure, sectoral breadth, or weighting schemes may plausibly contribute to heavier tails, particularly for global indices such as SPGCE, the present analysis does not seek to disentangle these effects. Accordingly, cross-index comparisons are framed in terms of robustness and CI-based inference rather than causal attribution.

Based on Table 3, tail index estimates for both renewable and conventional energy indices are consistent with heavy-tailed return distributions characterized by power-law decay. Across indices and estimation methods, estimated tail indices exceed the threshold associated with infinite means and are generally above the boundary for finite second moments (i.e., ζ>2), while remaining below values typically associated with thin-tailed behavior. These findings align with extensive evidence documenting heavy-tailed returns in financial and commodity markets [21,25,93].

A formal comparison based on the reported CIs indicates that tail index estimates for renewable and conventional energy indices are not statistically distinguishable at conventional significance levels. Specifically, the CIs overlap across indices, truncation levels, and estimation approaches, implying that numerical differences in point estimates cannot be interpreted as statistically significant. Accordingly, CI-based inference does not support statistically meaningful differences in tail heaviness between renewable and conventional energy markets.

The tail index estimates in Table 3 further allow inference on the existence of statistical moments implied by power-law behavior. Under a power-law distribution, the magnitude of the tail index determines the highest finite moment, with ζ>1 implying a finite mean, ζ>2 implying a finite variance, and ζ>4 implying a finite fourth moment (see Section 2.3). Based on CI-based inference, the results provide strong support for the existence of finite first and second moments for both renewable and conventional energy indices. In particular, for all indices and estimation approaches, the LBs of the reported CIs lie above the threshold ζ=2, indicating finite variances. At the same time, CIs frequently extend below the threshold ζ=4, indicating that fourth moments and kurtosis may not be finite for several indices and specifications.

Inference regarding higher-order moments is less conclusive. In several cases, CIs extend across the threshold ζ=3, preventing definitive conclusions regarding the finiteness of third moments. These findings are consistent with a broad body of empirical evidence documenting financial return distributions characterized by finite variances but potentially infinite higher-order moments [21,25,93].

In addition to overall heavy-tailedness, asymmetry between upward and downward extremes is a well-documented stylized feature of financial return distributions (see [14,21,25] and references therein). Gain-loss asymmetry reflects the tendency for large negative returns to occur more frequently or with greater magnitude than large positive returns, particularly during periods of market stress. Empirical evidence further indicates that adverse shocks often exert a disproportionate impact on the lower tail of return distributions, while the upper tail remains comparatively less affected [94]. Despite its relevance for downside risk assessment, tail asymmetry in the context of power-law behavior has received limited attention in renewable energy markets. To examine potential asymmetries in tail behavior, Tables 4 and 5 report estimates of right-tail (positive returns) and left-tail (negative returns) indices based on the power-law specifications in equations (1) and (2). Results are presented for both the LLRS regression approach and Hill’s estimator at the 10% and 5% truncation levels, together with their corresponding CIs.

**Table 4:** The right tail index estimates for renewable and conventional energy indices.

Index	LLRS regression			Hill’s estimation			N[n]
	${\hat{\zeta }}_{RS}$	${SE}_{RS}$	95% CI [LB, UB]	${\hat{\zeta }}_{Hill}$	${SE}_{Hill}$	95% CI [LB, UB]	
**Part A. Truncated (10%)**							
**ECO**	3.419	0.301	[2.831, 4.008]	2.865	0.178	[2.516, 3.214]	2590 [259]
**SPGCE**	2.773	0.240	[2.303, 3.244]	2.680	0.164	[2.358, 3.001]	2674 [267]
**ERIX**	3.406	0.295	[2.828, 3.983]	3.068	0.188	[2.700, 3.436]	2679 [267]
**SUN**	3.260	0.286	[2.698, 3.821]	2.925	0.182	[2.568, 3.281]	2590 [259]
**DJUSEN**	3.014	0.264	[2.496, 3.533]	3.130	0.194	[2.750, 3.511]	2602 [260]
**Part B. Truncated (5%)**							
**ECO**	4.101	0.511	[3.100, 5.102]	3.412	0.300	[2.823, 4.001]	2590 [129]
**SPGCE**	2.965	0.364	[2.252, 3.677]	2.627	0.228	[2.180, 3.073]	2674 [133]
**ERIX**	3.506	0.430	[2.663, 4.348]	3.489	0.303	[2.896, 4.082]	2679 [133]
**SUN**	3.495	0.435	[2.642, 4.347]	3.271	0.288	[2.706, 3.835]	2590 [129]
**DJUSEN**	2.955	0.367	[2.236, 3.673]	3.104	0.272	[2.571, 3.638]	2602 [130]

Notes: Table 4 reports right-tail index estimates for renewable energy indices (ECO, SPGCE, ERIX, SUN) and the conventional energy index (DJUSEN) based on daily log returns. Right-tail indices correspond to extreme positive return realizations and are estimated using the LLRS regression approach with optimal rank shift 1/2 (${\hat{\zeta }}_{RS}$) and Hill’s estimator (${\hat{\zeta }}_{Hill}$). Notation and column definitions follow Table 3.

**Table 5:** The left tail index estimates for renewable and conventional energy indices.

Index	LLRS regression			Hill’s estimation			N[n]
	${\hat{\zeta }}_{RS}$	${SE}_{RS}$	95% CI [LB, UB]	${\hat{\zeta }}_{Hill}$	${SE}_{Hill}$	95% CI [LB, UB]	
**Part A. Truncated (10%)**							
**ECO**	3.344	0.305	[2.745, 3.942]	3.055	0.197	[2.668, 3.441]	2407 [240]
**SPGCE**	2.503	0.224	[2.063, 2.942]	2.400	0.152	[2.102, 2.698]	2495 [249]
**ERIX**	2.899	0.264	[2.382, 3.415]	2.652	0.171	[2.318, 2.987]	2423 [242]
**SUN**	2.874	0.255	[2.375, 3.373]	2.544	0.159	[2.232, 2.856]	2552 [255]
**DJUSEN**	2.770	0.253	[2.274, 3.265]	2.759	0.178	[2.410, 3.108]	2403 [240]
**Part B. Truncated (5%)**							
**ECO**	3.602	0.465	[2.690, 4.513]	3.436	0.314	[2.821, 4.051]	2407 [120]
**SPGCE**	2.665	0.338	[2.001, 3.328]	2.313	0.208	[1.906, 2.721]	2495 [124]
**ERIX**	3.421	0.440	[2.559, 4.283]	3.031	0.276	[2.491, 3.571]	2423 [121]
**SUN**	3.108	0.390	[2.344, 3.873]	3.036	0.269	[2.508, 3.564]	2552 [127]
**DJUSEN**	2.697	0.348	[2.015, 3.380]	2.923	0.267	[2.400, 3.446]	2403 [120]

Notes: Table 5 reports left-tail index estimates for renewable energy indices (ECO, SPGCE, ERIX, SUN) and the conventional energy index (DJUSEN) based on daily log returns. Left-tail indices correspond to extreme negative return realizations and are estimated using the LLRS regression approach with optimal rank shift 1/2 (${\hat{\zeta }}_{RS}$) and Hill’s estimator (${\hat{\zeta }}_{Hill}$). Notation and column definitions follow Table 3.

Across indices and truncation levels, point estimates of right- and left-tail indices differ in magnitude, indicating variation in the relative thickness of upper and lower tails. In several cases, point estimates can indicate that downward return movements are associated with heavier tails than upward movements, while for other indices and truncation levels the opposite pattern is observed. These patterns are not uniform across assets or estimation approaches, highlighting heterogeneity in tail behavior. However, formal comparisons based on CIs indicate that right- and left-tail indices are generally not statistically distinguishable at conventional significance levels. In particular, CIs for positive and negative tail indices overlap across indices, truncation levels, and estimation methods. As a result, differences implied by point estimates should be interpreted as directional tendencies rather than statistically established asymmetries. Overall, the asymmetry analysis provides suggestive but statistically inconclusive evidence regarding differences between extreme positive and negative return movements, consistent with existing findings for stock returns and exchange rates and with the broader volatility literature, where asymmetric volatility responses do not necessarily translate into distinct tail decay rates [25,57].

To further explore the time variation in tail risk, we analyze the dynamic evolution of tail indices using a recursive expanding-window estimation approach. Recursive estimation is widely used in forecasting and spillover analyses to capture changes in distributional properties over time, particularly in the presence of persistent extreme events (see, e.g., [95,96]). Following [35], the recursive procedure begins with an initial estimation window of 500 daily observations and then expands the sample one observation at a time until the end of the sample period. Once major extreme events occur, their effects carry over into subsequent estimation windows, allowing recursive tail indices to capture the evolving degree of tail heaviness through time. Unlike rolling-window approaches, recursive expanding-window estimation permanently retains the influence of past extreme events in subsequent estimation windows. Consequently, recursive tail indices primarily capture the persistence and long-run influence of crisis-period extremes rather than short-term mean reversion in tail behavior. A rolling-window framework may therefore produce more transitory fluctuations in estimated tail risk, whereas the recursive approach adopted here is better suited for assessing the lasting impact of systemic shocks on tail dynamics.

**Fig 3:**
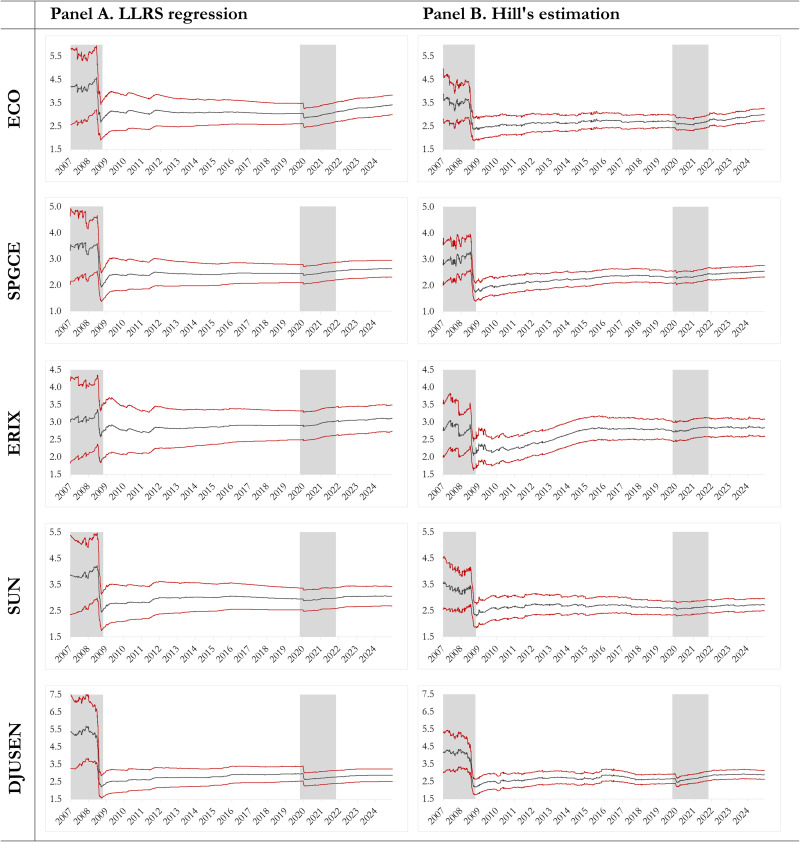
Recursive tail index estimates for renewable and conventional energy indices. Notes: Fig 3 reports recursive tail index estimates based on daily log returns using a recursive expanding window with an initial estimation window of 500 observations and a fixed truncation level of 10%. Panel A reports LLRS-based estimates using the optimal rank shift of 1/2, while Panel B reports estimates based on Hill’s estimator. Black lines denote point estimates and red lines indicate the corresponding 95% CIs. Grey shaded areas indicate the 2007–2008 GFC and the COVID-19 market shock in 2020–2021. Y-axes are standardized within each index to facilitate comparison of tail dynamics over time.

Fig 3 reports recursive tail index estimates for renewable and conventional energy indices based on a fixed truncation level of 10%, using the LLRS regression approach in Panel A and Hill’s estimator in Panel B. Across indices and estimation methods, the recursive estimates exhibit a pronounced decline during the 2007–2008 GFC, indicating a substantial increase in tail heaviness during this period of systemic financial stress. For certain indices, recursive tail estimates temporarily fall to levels consistent with ζ<2, implying that variance may not be finite during crisis periods. Such episodes highlight that standard finite-variance risk models may become unreliable during severe market stress, even if tail behavior stabilizes in subsequent periods.

After the GFC, recursive tail indices stabilize and evolve more gradually, indicating that the heightened tail risk induced by crisis-period extremes persists over time but does not continue to intensify. The post-crisis dynamics are broadly similar across renewable and conventional energy markets, suggesting that tail risk in clean energy assets responds to major global shocks in a manner comparable to traditional energy assets. A more modest change in tail behavior is observed around the onset of the COVID-19 pandemic in early 2020, particularly for the conventional energy index. The comparatively more modest tail-risk response during the COVID-19 period may reflect important differences in the underlying nature of the two crises. Whereas the GFC originated within the financial system and was characterized by severe banking distress, prolonged deleveraging, and widespread uncertainty regarding financial solvency, the COVID-19 shock was largely exogenous and accompanied by rapid monetary and fiscal policy interventions. These measures may have limited the persistence of systemic financial stress and contributed to a comparatively smaller deterioration in tail behavior across energy markets. While renewable energy indices also exhibit mild changes during this period, these shifts are considerably smaller than those observed during the GFC, consistent with broader evidence indicating the COVID-19 shock did not generate tail risk effects comparable to earlier systemic crises [42].

Taken together, the recursive analysis complements the static tail and asymmetry results by emphasizing the time-varying and state-dependent nature of tail risk in energy markets. Renewable energy investments display heavy-tailed dynamics similar to those of conventional energy assets, with tail risk responding strongly to major global shocks while remaining comparatively stable during more recent stress episodes. These results motivate a broader discussion of their implications for risk measurement, portfolio construction, econometric modeling, and policy considerations in renewable energy markets.

## 5. Discussion

Heavy-tailedness has implications of central importance for the robustness of conclusions drawn from many financial and economic models, including portfolio diversification, risk measurement, and asset pricing, with potential reversals of standard results depending on the degree of tail heaviness (see the comprehensive review in [21]). In particular, that monograph demonstrates that diversification remains optimal in VaR frameworks when risks are moderately heavy-tailed, that is, when tail indices satisfy ζ>1 and first moments are finite. By contrast, when risks are extremely heavy-tailed with ζ≤1 and infinite means, classical diversification results may fail or even reverse.

The existence of finite variances and higher moments is also a key assumption underlying many foundational models in economics and finance, including the CAPM and the Markowitz portfolio selection framework. More broadly, the finiteness of second and higher moments is essential for the validity of standard econometric tools, such as least squares regression, spectral analysis, and inference based on (auto)correlations. As discussed in [15,21,42,97,98], these classical methodologies may perform poorly or become invalid in heterogeneous, dependent, or heavy-tailed environments, which are frequently observed in financial and energy markets. This concern has motivated the use of robust inference approaches in recent empirical work. For example, [42] employ tail-robust methods to study the effects of the COVID-19 pandemic on global financial markets, documenting pronounced heavy-tailedness and potential non-stationarity in pandemic-related time series, while finding limited predictive power for stock returns. Similarly, [43] show that COVID-19 case counts in Chinese cities follow power-law distributions with tail indices near or below unity during early outbreak phases, implying infinite means and variances.

These considerations highlight the importance of reliable statistical inference on tail indices, as conclusions regarding tail heaviness are directly linked to the appropriateness and robustness of standard financial and econometric models. Tail-risk analysis is particularly critical when tail indices lie near key theoretical thresholds, such as ζ=1 (finite mean) or ζ=2 (finite variance), which determine the validity of many commonly used modelling and inference techniques.

Within this framework, the results of the present study provide evidence, based on CI-based inference rather than point estimates, that all renewable and conventional energy indices examined possess finite first and second moments, with tail indices exceeding ζ=2. This finding supports the applicability of risk measures and modelling approaches that rely on the existence of means and variances when analysing renewable energy asset returns. At the same time, the analysis indicates that higher-order moments, particularly third and fourth moments, may not be finite for several indices, which has important implications for portfolio construction and risk modelling.

From a risk-management perspective, these results clarify the conditions under which commonly used risk measures are appropriate. VaR is known to be non-coherent for distributions with ζ≤1, while expected shortfall (ES) is coherent only when first moments are finite (see [15,92,99]). Since CI-based inference in this study supports ζ>2 across all indices, both VaR and ES are theoretically well defined and applicable in the energy markets considered. This provides reassurance to investors, regulators, and risk managers that standard downside risk measures can be used meaningfully in renewable energy markets, while still warranting caution regarding tail-driven extreme losses. From a broader energy-transition perspective, elevated tail risk may increase financing costs and required risk premia for renewable energy firms, potentially affecting long-term capital allocation, infrastructure investment, and the pace of clean-energy deployment.

The findings also bear directly on portfolio theory. The classical Markowitz mean-variance (MV) framework, introduced in [100] and widely applied in studies such as [101], relies on the adequacy of first and second moments to characterize investment risk. However, a growing literature emphasizes that higher moments, particularly skewness, can materially affect optimal portfolio choice and asset pricing (see, among others, [102–105]). MVS portfolio approaches have been shown to outperform traditional MV methods in certain non-normal settings [106]. Importantly, such approaches are theoretically valid only when third moments are finite, that is, when ζ>3.

In this respect, the present results warrant a cautious interpretation. While tail indices are sufficiently large to support MV analysis, CI-based inference does not provide reliable support for the existence of finite third moments. This implies that portfolio optimization approaches relying on skewness may lead to highly unstable and erratic portfolio weights in practice and should therefore be applied with considerable caution in renewable energy markets. In finite samples, this finding should be interpreted as evidence of extremely large and unstable higher-moment behavior rather than literal divergence. Consequently, skewness- and kurtosis-based measures are unlikely to be estimated with sufficient precision to support reliable optimization or inference. Empirical evidence in [107] demonstrates that portfolio strategies incorporating skewness can perform poorly or become numerically unstable under heavy-tailed return distributions, reinforcing the need for caution when applying MVS frameworks in environments characterized by pronounced tail risk.

The implications extend to volatility modelling and related risk-forecasting tools. A substantial literature documents the use of GARCH-type and stochastic volatility (SV) models in financial markets (see, among others, [94,98,99,108–110]). For example, [22] apply GARCH, EGARCH, and APARCH models to estimate VaR and ES for renewable and conventional energy stocks, finding higher volatility and more frequent VaR violations in renewable energy assets. Given the evidence in this study that fourth moments may be infinite, inference based on autocorrelations and volatility clustering should be interpreted with care, as standard asymptotic properties may not hold in heavy-tailed settings [98]. These considerations are also relevant for regulatory stress-testing frameworks, where underestimation of tail heaviness may lead to insufficient capital buffers and inadequate assessment of systemic downside risk during periods of severe market stress.

Importantly, the results do not support the hypothesis that renewable energy assets are systematically less heavy-tailed than conventional energy assets in a statistically meaningful sense. While point estimates may differ, CI-based inference indicates no statistically significant differences in tail heaviness. Accordingly, claims of uniformly lower tail risk in renewable energy markets should be avoided. Although prior studies document lower volatility or higher efficiency in clean energy markets relative to conventional ones [44,111], the present findings emphasize that renewable energy assets remain exposed to substantial tail risk, particularly during periods of systemic stress. One possible explanation for these similarities is that both renewable and conventional energy equities are increasingly integrated into broader global financial markets and therefore remain exposed to common macro-financial shocks, liquidity contractions, and shifts in investor risk sentiment during crisis periods. As a result, systemic stress events may generate comparable tail-risk dynamics across energy sectors despite differences in underlying technologies and business models.

This perspective has important policy and investment implications. Mis-characterizing renewable energy assets as inherently ‘lower-risk’ could encourage excessive portfolio concentration or complacency in risk management. If tail risks are underestimated, particularly in the presence of climate policy uncertainty, regulatory shocks, or geopolitical disruptions, such over-allocation may amplify losses during adverse regimes. As highlighted by [112], asymmetry and tail dependence can materially increase downside risk even when average volatility appears low. The results therefore underscore the importance of prudent diversification, stress testing, and dynamic tail-risk monitoring in portfolios with significant exposure to renewable energy assets.

Finally, the recursive tail analysis reinforces these conclusions by demonstrating that tail risk in energy markets is both persistent and state-dependent. The sharp decline in tail indices during the 2007−2008 GFC, including temporary values below ζ=2 for some indices, signals heightened vulnerability to extreme events and underscores the limitations of standard risk models during crises. Subsequent stabilization and the comparatively milder tail response during the COVID-19 period highlight meaningful differences across shock types, reinforcing the value of tail indices as practical tools for identifying and monitoring systemic risk over time.

## 6. Conclusion

It is well established that financial asset returns deviate from Gaussianity and frequently exhibit heavy-tailed behavior, a stylized fact documented across equity, commodity, and financial markets (see [15,16,21], and references therein). As renewable energy markets continue to expand in response to global decarbonization efforts, investor participation in clean energy assets is expected to grow further. This development underscores the importance of accurately assessing downside risk and extreme events in renewable energy markets. In this context, heavy-tailedness analysis plays a critical role, as reliable inference on tail indices is a prerequisite for understanding tail risk and for evaluating the robustness of risk management, portfolio construction, and econometric modeling frameworks applied to renewable energy assets.

This study provides a comprehensive assessment of tail risk in major renewable energy indices using robust tail index estimation approaches, including Hill’s estimator and the LLRS regression with the optimal rank shift of −1/2. The analysis further incorporates recursive expanding-window estimation to capture the time-varying nature of tail risk and to identify periods of heightened vulnerability to extreme events. Throughout, renewable energy indices are benchmarked against a conventional energy index to assess whether statistically meaningful differences in tail behavior exist. Importantly, the empirical analysis consistently relies on CI-based inference rather than point estimates alone.

The results indicate that both renewable and conventional energy indices are characterized by heavy-tailed return distributions consistent with power-law behavior. CI-based inference supports the existence of finite first and second moments across all indices considered, implying that tail indices exceed the critical threshold ζ=2. At the same time, inference regarding higher-order moments remains inconclusive, as CIs frequently span the threshold ζ=3 and extend below ζ=4. These findings suggest that while MV-based modeling frameworks are generally admissible, higher moments such as skewness and kurtosis may not be reliably estimable, with important implications for portfolio optimization, econometric inference, and volatility modeling.

The gain-loss asymmetry analysis further provides suggestive but not statistically decisive evidence that upper and lower tails may differ across indices and truncation levels. While point estimates occasionally indicate heavier downside tails, overlapping CIs imply that such differences should be interpreted as directional tendencies rather than statistically established asymmetries. Accordingly, the analysis does not support strong conclusions regarding systematic dominance of downside or upside tail risk in renewable energy markets relative to conventional energy markets.

The recursive tail analysis highlights the state-dependent nature of tail risk in energy markets. Tail indices decline sharply during periods of systemic stress, most notably during the 2007−2008 GFC, with temporary values consistent with ζ<2 for some indices. Such episodes imply the potential breakdown of finite-variance assumptions during crises and underscore the limitations of standard risk models in extreme market environments. In contrast, tail responses during more recent stress episodes, including the COVID-19 period, are comparatively milder, pointing to meaningful differences in the transmission and persistence of tail risk across shock types. These findings emphasize the value of dynamic tail monitoring and stress testing in renewable energy portfolios. More broadly, the results highlight that understanding extreme downside risk is increasingly important as renewable energy assets become more deeply integrated into global financial systems and play a larger role in financing the energy transition.

Taken together, the results support the applicability of risk measures and modeling frameworks that rely on finite first and second moments, while simultaneously cautioning against uncritical reliance on higher-moment-based methods. In particular, while MV portfolio approaches remain admissible, CI-based evidence does not rule out infinite third moments, suggesting that skewness-based portfolio optimization methods may be unstable in renewable energy markets. This reinforces the discussion in Section 5 regarding the careful use of advanced portfolio and volatility models in heavy-tailed environments.

Several avenues for future research naturally emerge from this analysis. First, extending the framework to multivariate settings would allow investigation of tail dependence and systemic risk transmission across renewable energy indices and between renewable and conventional energy markets. Copula-based approaches provide a natural tool for modeling such dependence under heavy tails and for extending EVT methods to multivariate contexts. Relatedly, identifying and evaluating the economic, financial, and policy determinants of tail heaviness in renewable and conventional energy markets remains an important open research question (see [26]). Second, the recursive methodology employed in this study can be further developed into real-time tail monitoring systems for renewable energy portfolios, enabling timely detection of regime shifts in tail risk. Complementary fixed-length rolling window approaches may further clarify whether crisis-induced tail behavior reflects persistent structural shifts or gradually reverts once earlier extreme observations exit the estimation window, thereby providing additional insight into the persistence mechanism inherent in recursive estimation. Third, integrating tail-risk analysis with climate stress-testing frameworks, including NGFS scenario-based transition and physical risk assessments, represents a promising direction for linking financial tail risk with climate policy uncertainty.

From a methodological perspective, future work may build on recent advances in robust inference under heterogeneity and dependence. In particular, group-based and t-statistic-based inference approaches for tail index estimation [113,114] offer promising tools for addressing cross-sectional dependence and heterogeneous tail behavior across assets and markets. In parallel, alternative tail inference methods based on a fixed number of extreme observations [115,116] provide a complementary perspective grounded in EVT limit theory and may be especially useful in smaller samples. While such fixed-k approaches differ conceptually from Hill and LLRS estimators that rely on increasing numbers of tail observations, their robustness to volatility clustering and temporal dependence remains an open question. Addressing these issues, and integrating robust tail inference with dependence-aware and multivariate frameworks, constitutes an important agenda for future research on tail risk in renewable energy and financial markets.

## Supporting information

S1 Table A1: Ljung-Box test for serial dependence. (DOCX)

S2 Table A2: Tail index estimates (raw log returns vs. GARCH standardized residuals). (DOCX)

S3 Table A3: Augmented Dickey-Fuller (ADF) unit root tests for energy index price levels and returns. (DOCX)

S4 Table A4: Moment existence implied by CI-based tail index inference (total tail). (DOCX)

S1 Fig A1 GARCH(1,1) standardized residuals for energy indices. (TIFF)

S1 Data Sample data (price levels and log returns) used in the empirical analysis. (XLSX)
