# Supplementary material for: Tail risk, large fluctuations and downfalls in renewable energy markets
Source: PLoS One. 2026 Jul 15;21(7):e0351106. doi: 10.1371/journal.pone.0351106 (PMC13372164; doi:10.1371/journal.pone.0351106)
Supplement: S1 Table — (DOCX) [file pone.0351106.s001.docx]

**Table A1. Ljung-Box test for serial dependence.**

| **Index** | **Lag** | **p-value (\|r_t_\|)** | **p-value (r_t_^2^)** |
| --- | --- | --- | --- |
| **ECO** | 10 | <0.000 | <0.000 |
|  | 20 | <0.000 | <0.000 |
| **SPGCE** | 10 | <0.000 | <0.000 |
|  | 20 | <0.000 | <0.000 |
| **ERIX** | 10 | <0.000 | <0.000 |
|  | 20 | <0.000 | <0.000 |
| **SUN** | 10 | <0.000 | <0.000 |
|  | 20 | <0.000 | <0.000 |
| **DJUSEN** | 10 | <0.000 | <0.000 |
|  | 20 | <0.000 | <0.000 |
| Notes: Table A1 reports Ljung-Box test p-values for serial dependence in absolute (\|r_t_\|) and squared daily log returns (r_t_^2^). The null hypothesis of no serial correlation is rejected at conventional significance levels for all indices and lag orders, indicating pronounced volatility clustering. This motivates the use of GARCH filtering prior to tail index estimation. | | | |
