# Supplementary material for: Tail risk, large fluctuations and downfalls in renewable energy markets
Source: PLoS One. 2026 Jul 15;21(7):e0351106. doi: 10.1371/journal.pone.0351106 (PMC13372164; doi:10.1371/journal.pone.0351106)
Supplement: S2 Table — (DOCX) [file pone.0351106.s002.docx]

**Table A2. Tail index estimates (raw log returns vs. GARCH standardized residuals).**

| **Index** | **Tail** | **Truncation (%)** | **Estimator** | **Raw log returns** | | | **GARCH standardized residuals** | | |
| --- | --- | --- | --- | --- | --- | --- | --- | --- | --- |
|  |  |  |  | $\hat{\zeta}$ | **LB** | **UB** | $\hat{\zeta}$ | **LB** | **UB** |
| **ECO** | **Total** | 10% | LLRS_RS | 3.410 | 2.812 | 4.008 | 4.911 | 4.050 | 5.771 |
|  |  |  | Hill | 2.993 | 2.731 | 3.256 | 4.206 | 3.837 | 4.575 |
|  |  | 5% | LLRS_RS | 3.862 | 2.904 | 4.819 | 5.755 | 4.328 | 7.182 |
|  |  |  | Hill | 3.411 | 2.988 | 3.834 | 4.942 | 4.329 | 5.554 |
|  | **Right** | 10% | LLRS_RS | 3.419 | 2.587 | 4.252 | 5.212 | 3.935 | 6.490 |
|  |  |  | Hill | 2.865 | 2.516 | 3.214 | 4.328 | 3.798 | 4.858 |
|  |  | 5% | LLRS_RS | 4.101 | 2.686 | 5.517 | 6.288 | 4.109 | 8.467 |
|  |  |  | Hill | 3.412 | 2.823 | 4.001 | 5.124 | 4.237 | 6.012 |
|  | **Left** | 10% | LLRS_RS | 3.344 | 2.498 | 4.190 | 4.740 | 3.550 | 5.929 |
|  |  |  | Hill | 3.055 | 2.668 | 3.441 | 4.134 | 3.615 | 4.652 |
|  |  | 5% | LLRS_RS | 3.602 | 2.313 | 4.891 | 5.570 | 3.593 | 7.547 |
|  |  |  | Hill | 3.436 | 2.821 | 4.051 | 4.980 | 4.096 | 5.864 |
| **SPGCE** | **Total** | 10% | LLRS_RS | 2.625 | 2.173 | 3.078 | 4.232 | 3.502 | 4.961 |
|  |  |  | Hill | 2.536 | 2.317 | 2.755 | 3.520 | 3.217 | 3.824 |
|  |  | 5% | LLRS_RS | 2.800 | 2.116 | 3.483 | 4.823 | 3.646 | 6.000 |
|  |  |  | Hill | 2.557 | 2.245 | 2.869 | 4.442 | 3.900 | 4.985 |
|  | **Right** | 10% | LLRS_RS | 2.773 | 2.108 | 3.439 | 4.175 | 3.158 | 5.191 |
|  |  |  | Hill | 2.680 | 2.358 | 3.001 | 3.556 | 3.123 | 3.989 |
|  |  | 5% | LLRS_RS | 2.964 | 1.957 | 3.972 | 4.945 | 3.239 | 6.652 |
|  |  |  | Hill | 2.627 | 2.180 | 3.073 | 4.640 | 3.839 | 5.441 |
|  | **Left** | 10% | LLRS_RS | 2.503 | 1.881 | 3.124 | 4.243 | 3.207 | 5.278 |
|  |  |  | Hill | 2.400 | 2.102 | 2.698 | 3.588 | 3.150 | 4.026 |
|  |  | 5% | LLRS_RS | 2.665 | 1.727 | 3.603 | 4.726 | 3.095 | 6.356 |
|  |  |  | Hill | 2.313 | 1.906 | 2.721 | 4.093 | 3.386 | 4.799 |
| **ERIX** | **Total** | 10% | LLRS_RS | 3.110 | 2.571 | 3.650 | 4.063 | 3.359 | 4.768 |
|  |  |  | Hill | 2.827 | 2.582 | 3.072 | 3.390 | 3.096 | 3.684 |
|  |  | 5% | LLRS_RS | 3.386 | 2.555 | 4.217 | 4.606 | 3.475 | 5.737 |
|  |  |  | Hill | 3.108 | 2.727 | 3.490 | 4.107 | 3.603 | 4.612 |
|  | **Right** | 10% | LLRS_RS | 3.406 | 2.589 | 4.223 | 4.654 | 3.516 | 5.792 |
|  |  |  | Hill | 3.068 | 2.700 | 3.436 | 3.734 | 3.278 | 4.191 |
|  |  | 5% | LLRS_RS | 3.506 | 2.314 | 4.697 | 5.225 | 3.415 | 7.036 |
|  |  |  | Hill | 3.489 | 2.896 | 4.082 | 5.013 | 4.144 | 5.881 |
|  | **Left** | 10% | LLRS_RS | 2.899 | 2.168 | 3.629 | 3.713 | 2.798 | 4.628 |
|  |  |  | Hill | 2.652 | 2.318 | 2.987 | 3.177 | 2.786 | 3.569 |
|  |  | 5% | LLRS_RS | 3.421 | 2.202 | 4.640 | 4.418 | 2.875 | 5.961 |
|  |  |  | Hill | 3.030 | 2.491 | 3.570 | 3.985 | 3.289 | 4.680 |
| **SUN** | **Total** | 10% | LLRS_RS | 3.051 | 2.525 | 3.578 | 4.166 | 3.447 | 4.885 |
|  |  |  | Hill | 2.711 | 2.477 | 2.945 | 3.468 | 3.169 | 3.767 |
|  |  | 5% | LLRS_RS | 3.303 | 2.497 | 4.109 | 4.747 | 3.588 | 5.905 |
|  |  |  | Hill | 3.052 | 2.680 | 3.425 | 4.163 | 3.655 | 4.671 |
|  | **Right** | 10% | LLRS_RS | 3.260 | 2.466 | 4.054 | 4.106 | 3.098 | 5.114 |
|  |  |  | Hill | 2.925 | 2.568 | 3.281 | 3.460 | 3.035 | 3.885 |
|  |  | 5% | LLRS_RS | 3.495 | 2.288 | 4.701 | 4.830 | 3.150 | 6.510 |
|  |  |  | Hill | 3.271 | 2.706 | 3.835 | 4.166 | 3.442 | 4.891 |
|  | **Left** | 10% | LLRS_RS | 2.874 | 2.168 | 3.579 | 4.172 | 3.160 | 5.184 |
|  |  |  | Hill | 2.544 | 2.232 | 2.856 | 3.638 | 3.197 | 4.080 |
|  |  | 5% | LLRS_RS | 3.108 | 2.027 | 4.189 | 4.594 | 3.014 | 6.173 |
|  |  |  | Hill | 3.036 | 2.508 | 3.564 | 4.101 | 3.396 | 4.806 |
| **DJUSEN** | **Total** | 10% | LLRS_RS | 2.878 | 2.375 | 3.382 | 4.599 | 3.794 | 5.404 |
|  |  |  | Hill | 2.888 | 2.636 | 3.141 | 3.951 | 3.606 | 4.297 |
|  |  | 5% | LLRS_RS | 2.832 | 2.131 | 3.533 | 4.984 | 3.751 | 6.218 |
|  |  |  | Hill | 3.052 | 2.675 | 3.430 | 5.122 | 4.488 | 5.755 |
|  | **Right** | 10% | LLRS_RS | 3.014 | 2.282 | 3.747 | 5.312 | 4.003 | 6.621 |
|  |  |  | Hill | 3.130 | 2.750 | 3.511 | 4.449 | 3.901 | 4.997 |
|  |  | 5% | LLRS_RS | 2.955 | 1.939 | 3.970 | 5.798 | 3.773 | 7.823 |
|  |  |  | Hill | 3.104 | 2.571 | 3.638 | 5.630 | 4.647 | 6.613 |
|  | **Left** | 10% | LLRS_RS | 2.770 | 2.069 | 3.470 | 4.425 | 3.324 | 5.527 |
|  |  |  | Hill | 2.759 | 2.410 | 3.108 | 3.750 | 3.283 | 4.216 |
|  |  | 5% | LLRS_RS | 2.697 | 1.732 | 3.663 | 4.766 | 3.088 | 6.444 |
|  |  |  | Hill | 2.923 | 2.400 | 3.446 | 4.949 | 4.078 | 5.821 |
| Notes: Table A2 reports tail index estimates and 95% CIs (LB, UB) for daily log returns (Raw log returns) and for standardized residuals from a fitted GARCH(1,1) model (GARCH std. residuals). Estimates are computed for the total tail (based on absolute returns $\left\vert r \right\vert$) as well as separately for right and left tails, using truncation levels of 10% and 5% of the sample. Two estimators are reported: the LLRS regression with optimal rank shift 1/2 (implemented as $log(t-1/2)$), denoted LLRS_RS, and Hill’s estimator. The GARCH filtering step is used as a robustness check to mitigate volatility clustering and isolate tail behavior of innovations. | | | | | | | | | |
