# Supplementary material for: Tail risk, large fluctuations and downfalls in renewable energy markets
Source: PLoS One. 2026 Jul 15;21(7):e0351106. doi: 10.1371/journal.pone.0351106 (PMC13372164; doi:10.1371/journal.pone.0351106)
Supplement: S3 Table — (DOCX) [file pone.0351106.s003.docx]

**Table A3. Augmented Dickey-Fuller (ADF) unit root tests for energy index price levels and returns.**

| **Index** | **Specification** | **ADF Stat. (**$\boldsymbol{\tau}$**)** | **CV (5%)** | **Decision (Unit Root)** |
| --- | --- | --- | --- | --- |
| **Panel A. Price levels** |  |  |  |  |
| **ECO** | None | -1.287 | -1.95 | Non-Stationary |
|  | Drift | -1.684 | -2.86 | Non-Stationary |
|  | Trend | -1.975 | -3.41 | Non-Stationary |
| **SPGCE** | None | -1.012 | -1.95 | Non-Stationary |
|  | Drift | -1.535 | -2.86 | Non-Stationary |
|  | Trend | -1.914 | -3.41 | Non-Stationary |
| **ERIX** | None | -0.604 | -1.95 | Non-Stationary |
|  | Drift | -1.684 | -2.86 | Non-Stationary |
|  | Trend | -1.574 | -3.41 | Non-Stationary |
| **SUN** | None | -1.254 | -1.95 | Non-Stationary |
|  | Drift | -1.948 | -2.86 | Non-Stationary |
|  | Trend | -2.440 | -3.41 | Non-Stationary |
| **DJUSEN** | None | 0.032 | -1.95 | Non-Stationary |
|  | Drift | -2.681 | -2.86 | Non-Stationary |
|  | Trend | -2.725 | -3.41 | Non-Stationary |
| **Panel B. Returns** |  |  |  |  |
| **ECO** | None | -21.629 | -1.95 | Stationary |
|  | Drift | -21.642 | -2.86 | Stationary |
|  | Trend | -21.641 | -3.41 | Stationary |
| **SPGCE** | None | -25.436 | -1.95 | Stationary |
|  | Drift | -25.437 | -2.86 | Stationary |
|  | Trend | -25.435 | -3.41 | Stationary |
| **ERIX** | None | -27.705 | -1.95 | Stationary |
|  | Drift | -27.705 | -2.86 | Stationary |
|  | Trend | -27.709 | -3.41 | Stationary |
| **SUN** | None | -47.223 | -1.95 | Stationary |
|  | Drift | -47.218 | -2.86 | Stationary |
|  | Trend | -47.217 | -3.41 | Stationary |
| **DJUSEN** | None | -41.270 | -1.95 | Stationary |
|  | Drift | -41.271 | -2.86 | Stationary |
|  | Trend | -41.268 | -3.41 | Stationary |
| Notes: Table A3 reports Augmented Dickey-Fuller (ADF) unit root test results for energy index price levels (Panel A) and daily log returns (Panel B). Tests are conducted under three deterministic specifications: no intercept (“None”), intercept only (“Drift”), and intercept with linear trend (“Trend”). The reported test statistic is the ADF $\tau$-statistic, with 5% critical values shown in the table. The null hypothesis is the presence of a unit root. Rejection of the null indicates stationarity. All price series are non-stationary in levels, while all return series are stationary across specifications. | | | | |
