# Supplementary material for: Tail risk, large fluctuations and downfalls in renewable energy markets
Source: PLoS One. 2026 Jul 15;21(7):e0351106. doi: 10.1371/journal.pone.0351106 (PMC13372164; doi:10.1371/journal.pone.0351106)
Supplement: S4 Table — (DOCX) [file pone.0351106.s004.docx]

**Table A4. Moment existence implied by CI-based tail index inference (total tail).**

| **Index** | **LLRS regression** | | | | **Hill's estimation** | | | |
| --- | --- | --- | --- | --- | --- | --- | --- | --- |
|  | **Mean** | **Var** | **Skew** | **Kurt** | **Mean** | **Var** | **Skew** | **Kurt** |
| **Panel A. Truncated (10%)** | | | | | | | | |
| **ECO** | Finite | Finite | Inconcl. | Inconcl. | Finite | Finite | Inconcl. | Inconcl. |
| **SPGCE** | Finite | Finite | Inconcl. | Inconcl. | Finite | Finite | Inconcl. | Inconcl. |
| **ERIX** | Finite | Finite | Inconcl. | Inconcl. | Finite | Finite | Inconcl. | Inconcl. |
| **SUN** | Finite | Finite | Inconcl. | Inconcl. | Finite | Finite | Inconcl. | Inconcl. |
| **DJUSEN** | Finite | Finite | Inconcl. | Inconcl. | Finite | Finite | Inconcl. | Inconcl. |
| **Panel B. Truncated (5%)** | | | | | | | | |
| **ECO** | Finite | Finite | Finite | Inconcl. | Finite | Finite | Inconcl. | Inconcl. |
| **SPGCE** | Finite | Finite | Inconcl. | Inconcl. | Finite | Finite | Inconcl. | Inconcl. |
| **ERIX** | Finite | Finite | Inconcl. | Inconcl. | Finite | Finite | Inconcl. | Inconcl. |
| **SUN** | Finite | Finite | Inconcl. | Inconcl. | Finite | Finite | Inconcl. | Inconcl. |
| **DJUSEN** | Finite | Finite | Inconcl. | Inconcl. | Finite | Finite | Inconcl. | Inconcl. |
| Notes: Table A4 summarizes the implications of CI-based tail index inference for the existence of statistical moments implied by power-law behavior. Results are based on LLRS regression and Hill tail index estimates reported in Table 3 for the total tail at truncation levels of 10% (Panel A) and 5% (Panel B). “Finite” indicates that the lower bound of the 95% CI exceeds the relevant threshold ($\zeta=1, 2, 3, 4$), supporting the existence of the corresponding moment. “Inconcl.” denotes inconclusive inference regarding moment existence and indicates that the corresponding CI includes the relevant threshold value. | | | | | | | | |
