# Supplementary material for: Tail risk, large fluctuations and downfalls in renewable energy markets
Source: PLoS One. 2026 Jul 15;21(7):e0351106. doi: 10.1371/journal.pone.0351106 (PMC13372164; doi:10.1371/journal.pone.0351106)
Supplement: Attachment — Submitted filename: Point-by-point Response to Reviewers.docx [file pone.0351106.s009.docx]

**PLoS ONE - Reviewers' Comments and Authors' Responses**

**Title: Tail Risk, Large Fluctuations and Downfalls in Renewable Energy Markets**

We sincerely thank the Academic Editor and reviewers for providing us with very helpful and constructive comments and suggestions, which have helped us to improve the paper. We took on board all the issues raised and addressed them, as outlined below. We hope that our responses to the comments in the reviews are satisfactory.

The following tables show the Editor’s and the reviewers’ comments on the left and summarise the changes made in the paper following them on the right. Based on the feedback, we have thoroughly revised the earlier version of the manuscript. All results presented in the tables and figures have been updated using the most recent data available. Our main results remain unchanged. The data used in the analysis is now available and is submitted together with the revised version of the paper, as is discussed below.

| **Editor’s comments** | **Authors' Responses** |
| --- | --- |
| 1. Please ensure that your manuscript meets PLOS ONE's style requirements, including those for file naming. The PLOS ONE style templates can be found at  <https://journals.plos.org/plosone/s/file?id=wjVg/PLOSOne_formatting_sample_main_body.pdf>  and  <https://journals.plos.org/plosone/s/file?id=ba62/PLOSOne_formatting_sample_title_authors_affiliations.pdf> | Thank you very much for the comments. We have addressed the journal’s requirements in the style of the manuscript using the provided templates.  In particular, author information has been revised according to the template. The format of major section names has been further revised at level 1. Sub-sections have become level 2. The names of the figures and the corresponding references have also been adjusted according to the format style. We have removed the figures from the manuscript and are providing the images in a separate folder. We have also formatted the entire article in double-spaced text. We have also adjusted the reference style according to the journal's suggested style. |
| 2. We note that you have indicated that there are restrictions to data sharing for this study. PLOS only allows data to be available upon request if there are legal or ethical restrictions on sharing data publicly. For more information on unacceptable data access restrictions, please see  <http://journals.plos.org/plosone/s/data-availability#loc-unacceptable-data-access-restrictions>.  a) If there are ethical or legal restrictions on sharing a de-identified data set, please explain them in detail (e.g., data contain potentially identifying or sensitive patient information, data are owned by a third-party organization, etc.) and who has imposed them (e.g., a Research Ethics Committee or Institutional Review Board, etc.). Please also provide contact information for a data access committee, ethics committee, or other institutional body to which data requests may be sent.  b) If there are no restrictions, please upload the minimal anonymized data set necessary to replicate your study findings to a stable, public repository and provide us with the relevant URLs, DOIs, or accession numbers. For a list of recommended repositories, please see  <https://journals.plos.org/plosone/s/recommended-repositories>. You also have the option of uploading the data as Supporting Information files, but we would recommend depositing data directly to a data repository if possible.  We will update your Data Availability statement on your behalf to reflect the information you provide. | Many thanks for the comments. We are uploading the data used in the study as Supporting Information files in a zip file together with the revised manuscript. The zip file includes the .txt files with the values of daily indices used in the study for the period from April 2005 to February 2025, as discussed in Section 3.4. If necessary, the data will be uploaded to a repository online.  Since we are using secondary data (market indices), there are no ethical or legal restrictions on sharing the data. |
| Additional Editor Comments:  What are the main contributions of this study? Please add them in the introduction.  The last paragraph of the introduction should "introduce" the remaining parts of the paper. | Thank you for highlighting this point. We have further highlighted the research gaps in the literature and the paper’s contribution in the introduction on pages 4 and 6, among others. This also addresses the comments by reviewers, e.g., those by Reviewer 1 (Comment 3) and Reviewer 2 (Comment 1).  As suggested, we have included a brief discussion of the structure of the paper and the remainder of the manuscript in the last paragraph of the introduction.  “*The remainder of the paper is organised as follows. Section 2 reviews the relevant literature. Section 3 discusses the methodological approaches and data. Section 4 presents the main empirical findings and results, followed by their discussion in Section 5. Finally, Section 6 concludes*.” |

**Reviewer 1**

| **Reviewer's Comments** | **Authors' Responses** |
| --- | --- |
| **Comments on Abstract and Introduction** | |
| 1. **Abstract**   The abstract outlines the study’s goals and methods but should provide information about the analyzed period.  Furthermore, to maximize the article’s impact, it should highlight the contributions and implications of the study. The abstract's first sentence should be about the problem, motivation, or question. The second sentence should be about what we already know from the literature. In the third sentence, please say what you are doing and how; the following two sentences should be about what you find, followed by the significance of your findings. It is important to say what the question is; why this is an interesting and important question; how you go about it; why this is a persuasive modeling strategy; what you found; how this changes our view of the world (focusing on its novelty in relation to what we already know from the literature). People only spend one or two minutes reading an abstract; if they do not see the novelty within that time, they tend to ignore a paper. Our aim is to ensure that your paper gets cited after publication. Please consider highlighting the big ideas clearly and succinctly that is informative, novel, logical, and engaging. A reader will not be interested only in what you found but why you found it and how it helps solve a problem with more focus on practical implications/economic story rather than pure technical results; if you can add why you find something or why you have done something, they can have a bigger impact than just saying what the paper is doing.  Please consider improving it. | Thank you very much for your helpful comments and suggestions.  We have rewritten the abstract, taking into account the points raised in this comment.  **Abstract**  “*The renewable energy sector is rapidly expanding as nations intensify their efforts toward decarbonization, yet its vulnerability to extreme market fluctuations remains underexplored, particularly in heavy-tailedness and tail risk. Although financial asset returns often deviate from Gaussian distributions, researchers have not fully explored the unique risk dynamics of renewable energy markets. This study fills this gap by analyzing the heavy-tailedness properties of renewable and conventional energy stock indices using daily data from 2005 to 2025. We employ robust log-log rank-size regressions with optimal rank shifts and valid standard errors to examine the distributional tails of returns. Our findings indicate that while renewable energy stocks exhibit heavy-tailedness with finite first and second moments, they likely have infinite higher moments, pointing to limitations for models that rely on skewness and kurtosis. The results suggest that renewable energy indices are less heavy-tailed than conventional energy markets, supporting their potential as relatively lower-risk investment alternatives. This work provides new insights for investors, portfolio managers, and policymakers by emphasizing the need for ongoing risk assessment and adjusting risk models to better reflect the unique characteristics of renewable energy markets, particularly in extreme events and asymmetric risk profiles*.” |
| 2. While climate change and renewable energy trends are discussed extensively, the connection to the specific research focus (tail risk in renewable energy markets) is introduced too late. Try to condense the discussion of general climate change trends and expand the link between heavy-tailedness and renewable energy finance. Try incorporating recent studies (e.g., heavy-tailedness in financial markets from the last five years). | In response to this comment, we have removed portions of the discussion on climate change and renewable energy trends, including the paragraphs and sentences in the introduction that begin with:  “*In 2015, 196 nations, including the world’s two major carbon dioxide emitters…*”  “*Deploying renewable energy is of utmost importance*…”  “*According to Bolatoff & Boyer (2009), who assessed the performance of 310 “green” companies across various sectors*…”  Alongside these changes, we removed references to some relatively older literature and works, retaining more recent and relevant studies.  To ensure a balanced flow, the paper includes a discussion of renewable energy with references like: “With the obligatory commitment to decarbonisation, governments globally have been ramping up efforts, including a wide array of policy interventions and financing initiatives to support the shift from conventional to clean and renewable energy and to improve energy efficiency (Atalla et al., 2021).”  We have incorporated several recent and relevant studies (Gu & Ibragimov, 2018; Chen & Ibragimov, 2019; Xing & Ibragimov, 2023) focusing on tail risk within broader financial markets. On pages 7-8, we have added the following paragraph discussing these studies.  *“Some relatively recent papers examine the heavy-tailedness and risk properties of stock returns in different markets, focusing on emerging markets and the impact of financial crises. Gu and Ibragimov (2018) analyze excess volatility in developed and emerging stock markets, comparing the tail distributions of stock returns to theoretical models. They find that developed markets generally follow the "Cubic Law of Stock Returns," while emerging markets exhibit heavier tails. After the 2008 financial crisis, both developed and emerging markets showed a drop in their tail index. Chen and Ibragimov (2019) focus on the Chinese A- and H-share markets, comparing the tail risk properties of these markets under different institutional environments. Their results indicate that the A- and H-share tail indices are similar, with some returns possibly exhibiting infinite second moments. Xing and Ibragimov (2023) analyze the 2015 Chinese stock market crash, finding structural breaks in heavy-tailedness and asymmetry in returns. They highlight liquidity and company size as key determinants of tail risk in the Chinese market and observe that the tail properties did not significantly change post-crisis.”* |
| 3. The introduction mentions financial implications but does not adequately explain how this study fills specific financial risk management literature gaps. Please clarify how this study contributes to gaps in existing literature on renewable energy volatility and financial modeling. | Thank you for pointing out this aspect. We have highlighted the research gap and contribution in the introduction on page 4.  “*According to the latter works, when the presence of heavy-tailedness becomes excessively prominent, it can lead to the reversal of standard conclusions of fundamental models in economics, finance and risk management, including those on diversification optimality and conditions for coherency of widely used risk measures such as value at risk, among others. Therefore, reliable and econometrically justified analysis of heavy-tailedness properties would help risk management tools perform better and generate more insightful results for risk managers, investors, policymakers and financial regulators. Many works in the literature have focused on the analysis of heavy-tailedness and tail risk properties in financial and commodity markets, and several studies are available for cryptocurrency markets (see, inter alia, Loretan and Phillips, 1994; Cont, 2001; Ibragimov et al., 2013, 2015; Ankudinov et al., 2017a,b; Gupta et al., 2021; Ghosh et al., 2023; and references therein). However, a limited number of works exist that primarily focus on heavy-tailedness in the renewable energy markets despite their importance. The highly volatile renewable energy markets represent an important setting for investigating the heavy-tailedness of asset returns. This paper aims to fill the above research gap in the existing literature on renewable energy markets. This study contributes to the financial risk management literature by examining tail risk dynamics in renewable energy markets - an area where extreme downside movements remain insufficiently understood. Using the log-log rank-size regression method with an optimal shift parameter and valid standard errors, we robustly estimate the tail behavior of return distributions, highlighting the inadequacy of standard models in capturing tail risks. Overall, the paper provides a comprehensive empirical assessment of heavy-tailedness in leading renewable energy indexes and evaluates the implications for risk modeling and portfolio strategies*.”  While the general implications for stakeholders regarding risk modeling and portfolio strategies are briefly noted here, the detailed implications are thoroughly discussed in Section 5. |
| 4. There is limited discussion on why Hill’s estimator and LLRS regression were chosen. | Thank you for bringing this point to our attention. The rationale for the choice of tail estimators considered is now further outlined in the Introduction on pages 4-5, supported by references to corresponding works in the literature. A detailed discussion of these estimators is provided in Sections 3.1, 3.2, and 3.3 of the manuscript.  The methods employed in the paper are the most widely used approaches to inference on tail exponents and the degree of heavy-tailedness. Hill's estimator is a semi-parametric tail index estimator that approximates the top *n* order statistics with a Pareto distribution and estimates the tail index 𝜁 using a maximum likelihood estimation approach.  Numerous studies have revealed that inferences drawn from tail indices estimated using Hill's estimators are subject to several limitations and problems. To illustrate, the analysis in Embrechts et al. (1997) and Gabaix and Ioannides (2004) has shown that the standard errors obtained can be underestimated in cases of finite samples and dependent heterogeneous observations. As commonly documented, financial returns exhibit volatility clustering, and extreme financial market returns are clustered in time. Thus, the returns’ observations are not i.i.d., and the i.i.d. standard errors may not be valid under dependence (see, among others, the discussion in Gabaix, 2009, Gabaix and Ibragimov, 2011, Ibragimov et al., 2015).  To address the above problems, several alternative methodologies have been developed to estimate the tail index. The OLS-based regression method, so-called log-log rank size regression (LLRS), remains one of the most prevalent methods to estimate the tail index. This is largely due to the simplicity of the LLRS regression approach to tail index estimation and the robustness of this approach, including that to dependence and deviations from exact heavy-tailed power laws (see Gabaix and Ibragimov, 2011, and the discussion in Ibragimov et al., 2015). |
| 5. Briefly mention the data sources and econometric methods to set the stage for subsequent sections. | Data, its sources, and econometric methodology have been briefly described in the introduction. Please see page 5 in the revised manuscript.  “*This study seeks to address the research gap in the literature on renewable energy markets by conducting a detailed heavy-tailedness analysis of associated stock prices and returns using a robust and statistically justified tail index estimation technique (i.e., the LLRS regression approach) with optimal rank shifts and correct standard errors. The analysis is based on five major energy-related indices - ECO, ERIX, SPG, SUN (representing renewable energy), and DJUSEN (as a conventional energy sector benchmark) - with daily data obtained from the Bloomberg Terminal, covering the period from 2005 to 2025.*” |
|  | |
| **LITERATURE REVIEW** | |
| 6. It lacks recent studies. There is minimal coverage of studies published in the last five years, particularly those addressing renewable energy markets and tail risk in the context of recent global crises, such as the COVID-19 pandemic, geopolitical conflicts, or heightened policy uncertainty around climate change (e.g., Bouri, E., Hammoud, R., & Kassm, C. A. (2023). The effect of oil implied volatility and geopolitical risk on GCC stock sectors under various market conditions. *Energy Economics*, *120*, 106617. <https://doi.org/10.1016/J.ENECO.2023.106617>; Gabriel, V., Dionísio, A., Almeida, D., & Ferreira, P. (2024). From wars to waves: geopolitical risks and environmental investment behaviour. *Post-Communist Economies*, 1–28. <https://doi.org/10.1080/14631377.2024.2323763>; Mirza, N., Abubakr, M., Thu, T., Nguyen, H., Arfaoui, N., & Oliyide, J. A. (2023). Are sustainable investments interdependent ? The international evidence. *Economic Modelling*, *119*(September 2021), 106120. <https://doi.org/10.1016/j.econmod.2022.106120>; Shahzad, U., Mohammed, K. S., Tiwari, S., Nakonieczny, J., & Nesterowicz, R. (2023). Connectedness between geopolitical risk, financial instability indices and precious metals markets: Novel findings from Russia Ukraine conflict perspective. *Resources Policy*, *80*, 103190. <https://doi.org/10.1016/J.RESOURPOL.2022.103190>; Almeida, D., Ferreira, P., Dionísio, A., & Aslam, F. (2025). Exploring the connection between geopolitical risks and energy markets. *Energy Economics*, *141*, 108113. <https://doi.org/10.1016/J.ENECO.2024.108113>; Zhang, W., He, X., & Hamori, S. (2022). Volatility spillover and investment strategies among sustainability-related financial indexes: Evidence from the DCC-GARCH-based dynamic connectedness and DCC-GARCH t-copula approach. *International Review of Financial Analysis*, *83*, 102223. <https://doi.org/10.1016/J.IRFA.2022.102223>). Furthermore, studies addressing the rapidly evolving nature of renewable energy markets, especially advancements in econometric modeling for volatility and tail risk, are notably absent. | As suggested, to enhance and build on the recent literature, we have incorporated these and other recent studies and linked them to the context of the paper in the Introduction. The studies recommended by the reviewer also help to shape the research gap addressed in our study.  Please see the Introduction on page 4:  “*Recent studies incorporate various global economic and financial events, including geopolitical events, into their analyses, applying diverse methodologies across sectoral equity markets in oil-exporting economies, sustainable investments, traditional and clean energy markets, and the interconnections among green financial assets (e.g., Zhang et al., 2022; Bouri et al., 2023; Gabriel et al., 2024; Shahzad et al., 2023; Almeida et al., 2025). Unlike previous studies, we examine the impact of global events within the context of inference on the degree of heavy-tailedness and tail risk using a recursive window approach employed in some earlier studies, such as Quintos et al. (2001) and Gu and Inbragimov (2018). A notable characteristic of recursive tail index and tail risk inference is that once extreme events occur, their extreme values persist and carry over into subsequent windows unless additional extreme values are observed as the window expands.”* |
| 7. There is a heavy focus on historical context. While historical studies provide valuable background, they dominate the discussion, leaving limited room for recent advancements or emerging topics like renewable energy market performance during global disruptions or the role of artificial intelligence in market forecasting. Expand the focus beyond the relationships between oil prices, technology stocks, and renewable energy, as these topics have been explored extensively in earlier works. Instead, emphasize more nuanced relationships, such as the interplay between renewable energy policy changes and market volatility. Introduce recent themes like green energy transitions and carbon trading and their implications for renewable energy stocks. | In the literature review, we have included several studies that examine the causes of heightened fluctuations in renewable energy markets. The studies, such as Henriques and Sadorsky (2008), Chen et al. (2011), Sadorsky (2012), Reboredo (2015), and Bondia et al. (2016), investigate renewable energy markets and their interrelationships with conventional energy and other markets using various methodologies.  The paper also discusses the existing literature that has extensively explored the dynamic interrelationship among renewable and non-renewable energy markets and technology stocks, with many studies demonstrating evidence of dependence, causality and transmission. In this regard, many studies have indicated that upward movements in oil prices lead to favourable returns on renewable energy equity prices, which ultimately causes the technology stock price to increase.  The paper further discusses that, unlike earlier studies, our analysis fills the research gap by exploring heavy-tailedness and tail risk properties of renewable energy markets using advanced robust and statistically justified inference methods. In this direction, we focus on applications of robust log-log rank-size regressions with optimal rank shifts and asymptotically valid standard errors, providing a more robust and subtle understanding of markets' tail behaviour. |
| 8. There is a limited connection to heavy-tailedness in energy markets. Although heavy-tailedness is a central theme of the paper, the literature review does not delve deeply into recent works that explore heavy-tailed distributions in renewable energy or broader financial markets (e.g., studies applying advanced econometric techniques, such as machine learning approaches, to model heavy-tailed risks are missing). | Thank you very much for the comment. We have incorporated several recent and relevant studies (Gu & Ibragimov, 2018; Chen & Ibragimov, 2019; Xing & Ibragimov, 2023) that focus on heavy-tailedness, heavy-tailed distributions and tail risk within broader financial markets. On pages 7-8, we have added the following paragraph discussing these studies.  “*Some relatively recent papers examine the heavy-tailedness and tail risk properties of stock returns in different markets, focusing on emerging markets and the impact of financial crises on them. Gu and Ibragimov (2018) analyze excess volatility in developed and emerging stock markets, comparing the observed heavy-tailedness and tail risk properties of stock returns to those implied by theoretical models. They find that developed markets generally follow the "Cubic Law of Stock Returns," while emerging markets exhibit heavier tails. After the 2008 financial crisis, both developed and emerging markets exhibited a drop in their tail index and more pronounced heavy-tailedness properties. Chen and Ibragimov (2019) focus on the Chinese A- and H-share markets, comparing the tail risk properties and heavy-tailed distributions in these markets under different institutional environments. Their results indicate that the A- and H-share tail indices are similar, with some returns possibly exhibiting infinite second moments. Xing and Ibragimov (2023) analyze the 2015 Chinese stock market crash, finding structural breaks in heavy-tailedness properties of returns. They highlight liquidity and company size as key determinants of the tail risk in the Chinese stock market and observe that the tail risk properties did not significantly change post-crisis*.”  The paper further discusses that, unlike these studies, which focus on broader stock markets, our research concentrates explicitly on the renewable energy markets. Using robust and statistically justified tail index estimation methods, we examine the heavy-tailedness of returns in this unique sector. Additionally, our sample includes recent events such as pandemics alongside financial crises, offering a more comprehensive view of how these markets behave in response to economic and other global shocks. This allows us to explore the distinct risk characteristics of renewable energy markets in the context of recent disruptions. |
| **METHODOLOGY AND DATA** | |
| 9. The assumptions are not entirely justified. For example, 5% and 10% truncation levels for extreme observations are mentioned but not adequately justified. Justify the choice of truncation levels. | Thank you very much for this important comment. In the revised version of the paper, we employ analogues of “Hill’s plots” constructed using the log-log rank-size regression with an optimal shift parameter to analyze tail index estimates obtained using different tail truncations. These plots were generated for the renewable energy indices and are presented in Figure 2. These plots display the estimated tail indices, $\hat{\zeta}_{LLRS}$, across a range of truncation levels (i.e., 2.5%, 5%, 7.5%, 10%, 12.5%, and 15%), along with their 95% confidence intervals. The estimates remain relatively stable across different truncation levels, and the overlap of the confidence intervals indicates no statistically significant differences in the tail index estimates across these levels. The results obtained in this study appear to be relatively robust to variations in truncation levels.  We added the following paragraph in Section 4.2.  “*To support the choice of tail truncation levels (5% and 10%) employed in the analysis, we draw on the methodology and recommendations of previous studies (e.g., Embrechts et al., 1997; Ibragimov et al., 2013; Chen & Ibragimov, 2019). Specifically, we present Hill-type plots based on the LLRS regression tail index estimates for the renewable energy indices (see Figure 2). These plots display the estimated tail indices,* $\hat{\zeta}_{LLRS}$*, across a range of truncation levels (i.e., 2.5%, 5%, 7.5%, 10%, 12.5%, and 15%), along with their 95% confidence intervals. The estimates remain relatively stable across different truncation levels, and the overlap of the confidence intervals indicates no statistically significant differences in the tail index estimates across these levels. This suggests that power-law behavior in the tails of the return distributions for the renewable energy indices is robust to the chosen truncation values. Furthermore, the figures confirm that the core conclusions regarding the (in)finiteness of the second, third, and fourth moments remain unaffected by the chosen truncation level*.” |
| 10. There is no sensitivity analysis, i.e., no discussion of how results might vary with different truncation levels or alternate methods. | As discussed above, the revised version of the paper now presents, in Section 4.2, the assessment of robustness of the inference results and conclusions on heavy-tailedness using Hill’s plots (Figure 2). According to the analysis using Hill plots, the results and conclusions in the study appear to be relatively robust to the choice of tail truncation levels. |
| 11. Was the stationarity of the data analyzed? Was the data stationary? Please provide the results of stationarity tests. | Many thanks for the comment. We have performed the ADF test and presented the results in Appendix A to this document. Due to space considerations, the ADF test results were not included in the manuscript but are available from the authors upon request. We used the urca package in R to conduct the ADF test.  The ADF test results indicate that all five series (DJUSEN, ECO, ERIX, SPG, and SUN) are non-stationary in levels, as the null hypothesis of a unit root cannot be rejected across all model specifications (including autoregressions with a drift and those with a drift and time trend). However, when transformed to returns, the ADF statistics for all five series become highly significant across all model specifications. This leads to rejecting the unit root null hypothesis, confirming that the returns of DJUSEN, ECO, ERIX, SPG, and SUN are stationary. This is consistent with typical financial time series behaviour, where price series tend to be non-stationary, while return series are stationary.  We have added the following paragraph on ADF test results in Section 4.1.  “*The ADF test results indicate that all five series (DJUSEN, ECO, ERIX, SPG, and SUN) are non-stationary in levels, as the null hypothesis of a unit root cannot be rejected across all model specifications (including autoregressions with a drift and those with a drift and time trend). However, when transformed to returns, the ADF statistics for all five series become highly significant across all model specifications. This leads to rejecting the unit root null hypothesis, confirming that the returns of DJUSEN, ECO, ERIX, SPG, and SUN are stationary. This is consistent with typical financial time series behaviour, where price series tend to be non-stationary, while return series are stationary. The ADF test results can be provided upon request.*” |
| 12. Data limitations. The data used was sourced from the Bloomberg terminal. However, its restricted availability may not follow the *Plos One* data-sharing policy, and no alternative public data sources are suggested. Address data-sharing limitations and suggest alternative publicly available datasets. | In response to this comment, we provide all the data used in this study. |
| 13. Explain the analyzed period (April 1, 2005, to July 29, 2022). Provide a strong explanation for the end date (July 29, 2022) or update the results (we are at the beginning of 2025; it does not seem adequate to accept studies with data only until 2022). | Many thanks for this comment. In the revised version all the results, including tables and figures, are updated using data up to February 25, 2025.  We have provided the relevant discussion of the data in Section 3.3.  “*The data on indices dealt with was extracted from a Bloomberg terminal. The data on both the renewable energy indices and the conventional energy index are daily. The time series on all the indices are for the period from 1 April 2005 to 25 February 2025, which includes 5,008 daily return observations for WilderHill Clean Energy Index, 5,112 daily return observations for European Renewable Energy Index, 5,176 daily return observations for S&P Global Clean Energy Index, 5,169 daily return observations for MAC Global Solar Energy Index, and 5,021 daily return observations for Dow Jones US Oil and Gas Index.*” |
| **RESULTS** | |
| 14. The section emphasizes tail index estimates but provides limited interpretation of gain/loss asymmetry or cross-index comparisons. Please expand the discussion on gain/loss asymmetry and its implications for risk management. | Thank you for the comment. We have included Tables 4 and 5 in the revised version of the paper that provide the results on gain and loss asymmetry, focusing on right and left tail behavior. We have expanded the discussion of gain/loss asymmetry in Section 4.2, with particular emphasis on cross-index comparisons. Additionally, we have refined the earlier discussion on right and left tail dynamics to enhance clarity and depth.  *“As is seen in Tables 4 and 5, the left and right tail point estimates are not similar for all indices analysed. It is noticed that the downward moves of SPG, SUN and DJUSEN appear to be somewhat more heavy-tailed than their upward moves. However, the point estimates reveal mixed patterns for the ECO and ERIX indexes across the 5% and 10% truncation levels. For ECO, the 5% truncation level indicates a heavier left tail, suggesting greater downside risk, whereas the 10% level points to a heavier right tail, implying more extreme gains. Similarly, ERIX exhibits opposing patterns: the 10% level shows a heavier left tail, while the 5% level suggests a heavier right tail. These results suggest the existence of some asymmetry in the tails of upward and downward movements, which aligns with existing literature that reveals asymmetry in the volatility of renewable energy stocks. Hill’s estimation results show that the left tail is heavier than the right one for ERIX, SPG, SUN, and DJUSEN across both truncation levels, indicating a higher likelihood of extreme losses. In contrast, Hill’s estimates suggest a heavier right tail for the ECO index, implying a greater probability of extreme gains.”* |
| 15. The results' robustness under different methodological assumptions (e.g., alternate truncation levels) is not discussed. Try to include robustness checks to ensure findings are not highly sensitive to methodological choices. | Thank you very much for these important comments and suggestions. As is discussed in the response to Comments 9 and 10, the revised version of the paper now presents the analysis of the results’ robustness to the choice of tail truncation using analogues of Hill’s plots for LLRS inference approach (Figure 2) in Section 4.2 in the revised manuscript. According to the analysis, the results obtained in this study appear to be relatively robust in terms of variations in tail truncation levels.  Although, as is discussed in the paper, LLRS tail index inference with an optimal shift in ranks has several advantages over widely used Hill’s estimation approach, we have also included the results and the discussion for Hill’s tail index inference method. As is discussed in the manuscript, one does not observe the substantial differences in the results and conclusions obtained using LLRS regressions with optimal shifts and Hill’s estimation approach.  The revised version of the paper further includes the following details:  “*Hill’s estimation results show that the left tail is heavier than the right one for ERIX, SPG, SUN, and DJUSEN across both truncation levels, indicating a higher likelihood of extreme losses. In contrast, Hill’s estimates suggest a heavier right tail for the ECO index, implying a greater probability of extreme gains.*”  The revised version of the paper includes the following discussion on page 22:  “*In a cross-index comparison based on Hill’s approach, the SPG index consistently exhibits the smallest right-tail and left-tail estimates at the 5% and 10% truncation levels. The largest left-tail Hill’s estimates are observed for the ECO index across both truncation levels, while the largest right-tail Hill’s estimates are associated with DJUSEN at the 10% level and ERIX at the 5% level*.”  Nevertheless, we primarily rely on the results obtained using the LLRS regression inference (with the optimal shift parameter) approach due to its advantages discussed above and in the paper including robustness of the inference approach.  The relevant discussion is provided at the beginning of the Results section.  “*Due to the problems of small sample bias of Hill's tail index estimation approach and its sensitivity to dependence in observations and deviations from power laws discussed in the previous section, the analysis and inference on the degree of heavy-tailedness and tail indices of energy indices in this paper is primarily based on applications of the LLRS regression approach. However, we emphasise that LLRS and Hill’s tail index inference approaches typically lead to similar (point) estimates and qualitative conclusions as in the case of the findings in Ibragimov et al., 2013 for foreign exchange rates in emerging markets*.”  Pages 19-20 further include the following discussion:  “*We reject the null hypothesis (i.e.,* $\zeta=1$*) in favour of* $\zeta>1$*for all indices, including renewable and conventional energy indices, according to both the LLRS regression and Hill's estimation procedures. Moreover, the null hypothesis* $\zeta=2$ *is rejected in favour of* $\zeta>2$ *for all indices by both of the approaches. The null hypothesis* $\zeta=3$ *is rejected in favour of* $\zeta<3$ *for SPG5%, SPG10% and SUN10% using Hill's approach and SPG10% by LLRS regression approach. We do not reject the null hypothesis* $\zeta=3$ *for ECO10%, ERIX5%, ERIX10%, SPG5%, SUN10%, SUN5%, DJUSEN5% and DJUSEN10% using the LLRS regression and for ECO10%, ECO5%, ERIX10%, ERIX5%, SUN5%, DJUSEN5% and DJUSEN10% using Hill’s estimation. The null hypothesis* $\zeta=4$ *is rejected in favour of* $\zeta<4$ *for all indices by Hill’s inference approach. We do not reject the null hypothesis* $\zeta=4$ *for ECO5% and ERIX5% by the LLRS regression approach.*” |
| 16. It is referred to as “This study's initialisation window is 100 days”. Please explain the selection of this window. Why was it selected? Were the results robust in different windows (e.g., 250, 500, 1000 observations)? | In the original version, the index computation approach was as follows: we began by taking the first 100 observations, selected the largest (in absolute value) 10% of observations regardless of sign, and computed the tail index. As a result, the initial values in the tail index plots exhibited significant fluctuations due to the small sample size. In response to your comment, we increased the initial window size to 500 observations, following the influential study by Quintos, Fan, and Phillips (2001), and recursively added one observation at a time to estimate the tail index until the end of the available sample (see Figure 3 and 4). This adjustment resulted in more stable estimates. We note that the LLRS estimation approach overcomes several limitations of Hill’s estimator. However, for completeness, we present the updated plots based on both LLRS and Hill’s estimates. Additionally, similar to Quintos, Fan, and Phillips (2001), we include time information on the *x*-axis to help identify potential events associated with changes in the tail index.  Figures 2 and 3, which are based on the updated data and the initial sample of 500 observations, indicate that our original findings regarding the impact of the major events remain consistent.  Following the above, we have revised the discussion based on the new graphs in Section 4.2 as follows.  “*The recursive window approach, which is a commonly used technique in spillover analysis and forecasting (e.g., see Tanin et al., 2022; Vellachami et al., 2023), allows one to analyse the dynamics of the tail distribution of each renewable energy index over the sample period. The recursive estimation involves setting a fixed initial sample data, which begins from a particular time t to estimate the tail indexes. Following Quintos et al. (2001), we set the size of the initial estimation window to 500 daily observations, gradually expanding it by adding one observation at a time until the end of the sample. In other words, the first tail index is estimated for return observations in the first 500 days of the sample period and then one keeps adding new observations in the sample used for estimation as the window expands. A notable characteristic of recursive tail estimation is that once extreme events occur, their effects and extreme values persist and carry over into subsequent windows together with incorporation of additional extreme values observed as the window expands. As a result, the recursive tail index can indicate the dynamics of the overall tail distribution through time. Figures 3 and 4 display the estimated tail indices under recursive windows using Hill's estimator and LLRS regression method.*”  And,  “*Graphically, all renewable energy indices exhibit similar patterns in the dynamics of the tail index through the recursive windows for both the LLRS regression and Hill's methods. Based on Figures 3 and 4, all the renewable energy tail indices experienced a sudden drop in late 2008, pointing to heavy-tailedness thus becoming more pronounced, with the tail index of SPG exhibiting the most significant drop to below 2.0. During the period, the tail index of ECO, estimated using the LLRS regression fell from 4.6 to 2.7 (3.82 to 2.33 based on Hill’s estimator); the tail index of ERIX fell from 3.42 to 2.62 (3.17 to 2.12); the tail index of SPG decreased from 3.65 to below 2.0 (3.42 to 1.76); and the tail index of SUN decreases from 4.3 to 2.5 (3.6 to 2.15). Following the decrease, ECO, ERIX, and SUN renewable energy indices consistently exhibit a stable tail exponent of approximately 3.0 according to LLRS regression, while SPG maintains a stable tail index of 2.5. The dynamics of the recursive tail index for the conventional energy index DJUSEN exhibits a pattern similar to that of the four renewable energy indices. During the same period, the tail index of DJUSEN encountered a drop from 4.5 to 2.2 (4.45 to 2.15 based on Hill’s estimator) and later stabilised at around 2.8.*” |
| **CONCLUSION** |  |
| 17. The Conclusion summarizes the study’s findings and reiterates their relevance to renewable energy markets and financial risk management. It restates the study's key contributions. However, the conclusions make broad claims about the relevance of tail risk without adequately referencing specific findings. Furthermore, the implications of data-sharing restrictions on replicability are not addressed, nor are there other limitations of the study. Please address these issues. | Many thanks for the comment and suggestions.  In the conclusion part, following this comment, we have also reported specific findings across three aspects: the finiteness of moments, the presence of heavy tails with left-right tail asymmetry, and sensitivity of tail estimates to global events, assessed through recursive window estimations.  The discussion in the conclusions section was revised with, in particular, inclusion of the following paragraphs.  “*All renewable energy indices exhibit finite first and second moments. Among the four indices, only SPG shows evidence of an infinite third moment. Overall, the conclusion regarding the finiteness of third and fourth moments is that all renewable energy indices considered have, or may have, infinite third moments. Meanwhile, DJUSEN has finite first and second moments but may exhibit infinite third and fourth moments. According to point estimates, the left tails of return’s distributions renewable energy market appear somewhat heavier than their right tails. However, the difference between the left and right tail indices and the degrees of heavy-tailedness is not statistically significant. Moreover, the results of right and left tail index estimation reveal consistent patterns: the SPG index exhibits the smallest tail estimates, indicating heavier tails, while the ECO indexes generally show the largest values, suggesting lighter tails. This pattern holds across different tail truncation levels and tail directions, highlighting systematic differences in tail behaviour across the energy indices. The sharp decline in tail indices during the 2008 financial crisis, particularly the drop in SPG’s index below 2.0, signals increased downside risk and greater vulnerability to extreme events. These findings highlight the importance of dynamic risk monitoring and stress testing in renewable energy portfolios. While the tail dynamics of DJUSEN during the COVID-19-induced oil shock in 2020 also reflect heightened risk, the decline in the tail index was noticeably less severe than during the subprime crisis. Similarly, changes in tail indices during geopolitical events, such as the Russia-Ukraine conflict, so far have been relatively modest. These differences highlight the importance of monitoring tail index movements across various types of shocks and reinforce the value of tail indices as practical tools for identifying systemic risk.*”  We also make all the data available. |

Once again, thank you very much for very useful and constructive comments and suggestions on the paper!

**Reviewer 2**

| **Reviewer's Comment** | **Authors' Responses** |
| --- | --- |
| The study addresses an important and timely topic, analyzing tail risks and extreme fluctuations in renewable energy markets, a crucial area for ESG investing and sustainable finance. The use of robust econometric techniques, such as the LLRS regression with optimal rank shifts and Hill's estimator, adds rigor to the heavy-tailedness analysis. The inclusion of multiple renewable energy indices, such as ECO, ERIX, SPG, and SUN, alongside a conventional energy index, provides a broad perspective for comparative analysis. The findings offer significant insights for investors, policymakers, and risk managers regarding portfolio diversification, tail risk management, and market behavior under extreme conditions. | Thank you very much for your constructive comments and suggestions to improve the earlier version of the manuscript. |
| 1. While the paper highlights the importance of renewable energy markets, the manuscript could better articulate how its findings advance existing knowledge or address gaps in the literature. | This comment is also closely aligned with the feedback provided by the other reviewer.  We have revised the discussion throughout the paper to highlight that the study addresses the following research gaps in the literature:  Firstly, existing literature on renewable energy markets mainly focuses on the interrelationships among renewable and non-renewable energy markets and technology stocks, with many studies demonstrating evidence of dependence, causality and shocks’ transmission. In this regard, several studies have indicated, for instance, that upward movements in oil prices lead to favourable returns on renewable energy equity prices.  Secondly, some studies (e.g., Gu and Ibragimov, 2018; Chen and Ibragimov, 2019; Xing and Ibragimov, 2023) on heavy-tail analysis focus on broader financial markets rather than specifically on renewable energy markets.  On the other hand, to our best knowledge, there is no study on comprehensive analysis on the tail risk measures and heavy-tailedness in the renewable energy markets.  Unlike earlier studies, our analysis fills the research gap in the literature by exploring the tail risk and heavy-tailedness of renewable energy markets using statistically justified robust tail index inference methods. In this direction, we focus on inference using robust log-log rank-size regressions with optimal rank shifts and asymptotically valid standard errors, providing a robust and subtle understanding of markets' tail risk behaviour.  We have further incorporated a discussion of several recent and relevant studies (Gu & Ibragimov, 2018; Chen & Ibragimov, 2019; Xing & Ibragimov, 2023) that focus on the analysis of the tail risk within broader financial markets. In particular, on page 8, we have included the following paragraph discussing these studies.  “*Some relatively recent papers examine the heavy-tailedness and tail risk properties of stock returns in different markets, focusing on emerging markets and the impact of financial crises on them. Gu and Ibragimov (2018) analyze excess volatility in developed and emerging stock markets, comparing the observed heavy-tailedness and tail risk properties of stock returns to those implied by theoretical models. They find that developed markets generally follow the "Cubic Law of Stock Returns," while emerging markets exhibit heavier tails. After the 2008 financial crisis, both developed and emerging markets exhibited a drop in their tail index and more pronounced heavy-tailedness properties. Chen and Ibragimov (2019) focus on the Chinese A- and H-share markets, comparing the tail risk properties and heavy-tailed distributions in these markets under different institutional environments. Their results indicate that the A- and H-share tail indices are similar, with some returns possibly exhibiting infinite second moments. Xing and Ibragimov (2023) analyze the 2015 Chinese stock market crash, finding structural breaks in heavy-tailedness properties of returns. They highlight liquidity and company size as key determinants of the tail risk in the Chinese stock market and observe that the tail risk properties did not significantly change post-crisis*.” |
| 2. Clearly define the unique contribution of this study compared to previous works on tail risks in renewable energy markets. | Thank you for pointing out this aspect. We have further highlighted the research gap in the literature and the paper’s contribution in the introduction on page 4.  “*Many works in the literature have focused on the analysis of heavy-tailedness and tail risk properties in financial and commodity markets, and several studies are available for cryptocurrency markets (see, inter alia, Loretan and Phillips, 1994; Cont, 2001; Ibragimov et al., 2013, 2015; Ankudinov et al., 2017a,b; Gupta et al., 2021; Ghosh et al., 2023; and references therein). However, a limited number of works exist that primarily focus on heavy-tailedness in the renewable energy markets despite their importance. The highly volatile renewable energy markets represent an important setting for investigating the heavy-tailedness of asset returns. This paper aims to fill the above research gap in the existing literature on renewable energy markets. This study contributes to the financial risk management literature by examining tail risk dynamics in renewable energy markets - an area where extreme downside movements remain insufficiently understood. Using the log-log rank-size regression method with an optimal shift parameter and valid standard errors, we robustly estimate the tail behavior of return distributions, highlighting the inadequacy of standard models in capturing tail risks. Overall, the paper provides a comprehensive empirical assessment of the tail risk and heavy-tailedness in leading renewable energy indexes and evaluates the implications for risk modeling and portfolio strategies*.” |
| 3. While the selected indices are relevant, the manuscript could provide more justification for their inclusion. For instance, are these indices the most representative of global renewable energy markets? The analysis would benefit from explaining whether the selected indices adequately represent regional diversity (e.g., North America, Europe, Asia). Including indices from emerging markets could add value, given their growing role in renewable energy. | Thank you for the comment. We provide justification for the selected indices in Section 2.2 and data definitions in Section 3.3 in a table. As noted by Dawar et al. (2021), these stock indexes monitor the performance of the largest companies in the global renewable energy sector.  The selected indices offer strong comparative and insightful depth by capturing both clean and conventional energy sectors. Their geographic diversity (U.S., Europe, global) and sectoral coverage (broad renewables and solar-specific) enable a thorough evaluation of investment behavior, capital allocation, and tail risk exposure across energy markets transitioning toward sustainability.  **ECO**: This modified, equally weighted index includes publicly traded companies operating in the United States whose business activities significantly contribute to the societal shift towards cleaner energy and conservation.  **ERIX**: This index monitors the progress of European clean energy firms engaged in one or more investment groups: geothermal, biofuels, marine, water, solar, and wind.  **SPGCE**: This index evaluates the performance of companies involved in global renewable energy-related industries, encompassing advanced and developing countries. The index aims to include 100 constituents as its target count.  **SUN**: This index monitors publicly listed companies worldwide that specialise in offering solar energy products and services.  **DJUSEN**: This index tracks the performance of oil and gas companies explicitly working within the United States.  Including indices from emerging markets is an excellent suggestion, and the relevant analysis is indeed important; however, due to the broadness of the detailed analysis, it is beyond the current study and will be addressed in future research. We have discussed the suggestions on future research in this and related directions on page 15 and the conclusions section. In particular, this includes the following discussion.  “*We thank the reviewer for highlighting the importance of the analysis of heavy-tailedness of indices from emerging markets, given their growing role in the renewable energy sector. A study in this direction will be the focus of future research*.”  To further emphasize the geographic diversity and sectoral coverage of the selected indices, we have included the following discussion in Section 3.3:  *“Hence, this study covers clean and conventional energy sectors using indices representing geographic diversity (U.S., Europe, global) and sectoral coverage (broad renewables and solar-specific ones).”* |
| 4. The manuscript should better explain why the LLRS regression and Hill's estimator are the most suitable methods for analyzing renewable energy indices. Comparing their advantages over alternative methodologies, such as EVT (Extreme Value Theory) or Copula-based approaches, would strengthen the study. The interpretation of tail index estimates and their implications for risk management could be more elaborated, especially for indices like SPG with pronounced heavy-tailed properties. | Many thanks for this comment.  As is well-known (see, among others, Ch. 6 in Embrechts et al., 1997, Ch. 5 in McNeil et al., 2015), the analysis and inference on heavy-tailed power law distributions are closely linked to Extreme Value Theory with its powerful tools for discrimination in the tails such as the limit theorems for maxima and, more generally, order statistics of observations. Several recent papers have focused on tail index inference approaches based on a fixed number of extreme observations using the above EVT limit theorems and their implications (see Müller and Wang, 2017, Toda and Wang, 2020). The approaches are qualitatively different from the methods like Hill’s and the LLRS inference that are based on the normal asymptotics of tail index estimators computed using extreme observations whose number *k* increases with the total sample size *n*. The fixed *k* inference approaches are especially useful in the case of relatively small sample sizes. At the same time, the inference methods require i.i.d.ness assumptions for the data used in observations that are typically not satisfied for returns with volatility clustering and dependence over time. Robustness of the inference approaches to dependence is yet unclear, and their extensions to dependent samples constitute an important open research problem that future studies may focus on, together with applications of the methods in the analysis of heavy-tailedness in financial and renewable energy markets. We should also note that large sample sizes of daily returns dealt with in this paper together with robustness of LLRS tail index inference to dependence discussed in this paper and other studies provide further justification for methodology used in the analysis.  Embrechts, Klüppelberg and Extreme Value Theory (EVT) and heavy-tail estimators are closely related but serve different purposes. EVT generally involves two main approaches: (1) the Block Maxima Method and (2) the Peaks Over Threshold (POT) method. In the POT method, threshold selection is often challenging, and it assumes that exceedances are independent. However, this assumption may not hold if the series exhibits autocorrelation or volatility clustering (e.g., GARCH effects). Since renewable asset returns exhibit strong serial correlation and volatility clustering, the POT method may not be suitable in our context. Moreover, unlike EVT methods, LLRS regression or Hill’s estimators have useful applications, such as testing the finiteness of moments.  As the focus of the paper is on tail index inference for several univariate return time series, we have not considered copula-based approaches, as they are typically used to model dependence between two or more variables. As we highlighted in the literature review of the manuscript and the responses to other comments, the literature contains numerous studies that explore the dependence, causality, and relationships between renewable energy and other markets. Applications of copula theory in the analysis of interdependence among the renewable energy indices and dependence between financial and renewable energy markets constitute important directions for future research.  We have included a discussion of the above points in the overview of suggestions for future research in the conclusions section. |
| 5. What can an investor take away from Figures 2 and 3? The manuscript should explicitly highlight the practical insights these figures provide, particularly in terms of actionable strategies, risk management, or portfolio diversification. Clarifying these points will enhance the relevance of the analysis for investors | Thank you for the comment. We have revised the discussion of Figures 3 and 4 in the current version of the paper (Figures 2 and 3 in the previous version of the manuscript) to include more detail and to highlight the practical implications of the results on pages 25-27. Specifically, the sharp drop in tail indices during the 2008 financial crisis, particularly the fall of SPG’s tail index below 2.0, signals increased downside risk and heightened vulnerability to extreme events. These findings emphasize the importance of dynamic risk monitoring and stress testing in renewable energy portfolios. The post-crisis stabilization of tail indices, especially for ECO, ERIX, and SUN, around 3.0, suggests a return to more predictable risk environments, potentially supporting diversification benefits. The tail dynamics of DJUSEN during the COVID-19-induced oil shock in 2020 also indicate elevated risk, although the decline in the tail index was noticeably less pronounced than that during the subprime crisis. Similarly, the changes in tail indices during geopolitical crises (i.e., Russia-Ukraine conflict) have been relatively limited. These differences highlight the value of monitoring tail index movements across various types of shocks and reinforce the role of tail indices as practical tools for identifying and measuring systemic, tail and downward risk. We now make these points explicit to enhance the relevance of our analysis for investors focused on tail risk management and investment decision-making during turbulent periods.  We have expanded the discussion section by incorporating a discussion of the above findings and their implications. In particular, the revised version of the paper includes a discussion as follows.  “*The sharp drop in tail indices during the 2008 financial crisis, particularly the fall of SPG’s index below 2.0, signals increased downside risk and heightened vulnerability to extreme events. These findings emphasize the importance of dynamic risk monitoring and stress testing in renewable energy portfolios. The post-crisis stabilization of tail indices, especially for ECO, ERIX, and SUN, around 3.0, suggests a return to more predictable risk environments, potentially supporting diversification benefits. While the tail dynamics of DJUSEN during the COVID-19-induced oil shock in 2020 also indicate elevated risk, the decline in the tail index was noticeably less pronounced than during the subprime crisis. Similarly, the changes in tail indices during geopolitical crises (e.g., Russia-Ukraine conflict) have been relatively limited. These differences highlight the value of monitoring tail index movements across various types of shocks and reinforce the role of tail indices as practical tools for identifying systemic risk*.” |
| 6. The asymmetry in gain/loss behavior for renewable indices is a valuable insight but is not deeply explored. Expand the discussion on why such asymmetry exists and how it could influence investment strategies. | Many thanks for the comments. Tables 4 and 5 in the revised version of the paper present the results on gain and loss asymmetry, focusing on right and left tail behavior. We have expanded the discussion of gain/loss and tail asymmetry in Section 4.2, with particular emphasis on cross-index comparisons. Additionally, we have refined the earlier discussion on right and left tail dynamics to enhance clarity and depth.  “*As is seen in Tables 4 and 5, the left and right tail point estimates are not similar for all indices analysed. It is noticed that the downward moves of SPG, SUN and DJUSEN appear to be somewhat more heavy-tailed than their upward moves. However, the point estimates reveal mixed patterns for the ECO and ERIX indexes across the 5% and 10% truncation levels. For ECO, the 5% truncation level indicates a heavier left tail, suggesting greater downside risk, whereas the 10% level points to a heavier right tail, implying more extreme gains. Similarly, ERIX exhibits opposing patterns: the 10% level shows a heavier left tail, while the 5% level suggests a heavier right tail. These results suggest the existence of some asymmetry in the tails of upward and downward movements, which aligns with existing literature that reveals asymmetry in the volatility of renewable energy stocks.*  *A cross-index analysis reveals that, at the 10% truncation level, the SPG index exhibits the smallest right-tail estimate (2.70), while the ECO index has the largest (3.32). For the left tail, SPG10% records the smallest index (2.57), and ECO10% shows the highest (3.45). At the 5% level, DJUSEN shows the smallest right-tail index (2.87), followed by SPG, whereas ECO again shows the largest right-tail value (3.96). On the left tail, SPG5% has the smallest index (2.76), while ECO5% exhibits the largest (3.75). The results reveal consistent patterns: the SPG index exhibits the smallest tail estimates, indicating heavier tails, while the ECO indexes generally show the largest values, suggesting lighter tails. This pattern holds across truncation levels and tail directions, highlighting systematic differences in tail behaviour across the energy indices. In a cross-index comparison based on Hill’s approach, the SPG index consistently exhibits the smallest right-tail and left-tail estimates at the 5% and 10% truncation levels. The largest left-tail estimates are observed for the ECO index across both truncation levels, while the largest right-tail estimates are associated with DJUSEN at the 10% level and ERIX at the 5% level*.” |
| 7. Although the study has significant implications for policymakers, the recommendations are not explicitly discussed. Add a dedicated section summarizing actionable insights for policymakers aiming to stabilize renewable energy markets. | Thank you for the comment and suggestions. We have rewritten and clearly highlighted the implications and recommendations in Section 5.  One of the key findings of this study is that all renewable energy indices considered have tail indices greater than two. This indicates that all indices possess finite first and second moments, supporting the use of economic and financial models that rely on the finiteness of means and variances when modelling or forecasting renewable energy asset returns. Only SPG shows evidence of an infinite third moment among the four indices analyzed. The overall conclusion is that all renewable energy indices under consideration have, or may have, infinite third moments and infinite fourth moments.  For investors, regulators, and risk managers, the results of this study provide reassurance that standard risk measures such as Value at Risk (VaR) and Expected Shortfall (ES) are appropriate tools for analysing, modelling, and overseeing renewable energy markets, given the confirmed finiteness of first and second moments.  For portfolio managers and financial analysts, the literature highlights that beyond mean and variance, higher moments such as skewness play an important role in optimizing portfolio choice, asset pricing, and related decisions. Methods based on mean-variance-skewness (MVS) frameworks have been shown to yield superior portfolios when dealing with non-normally distributed assets. However, the findings of this study caution that, given the potential infiniteness of third moments in the renewable energy indices analysed, such skewness-based optimization approaches may not be applicable in these markets.  For risk managers, investors, and policymakers, it is important to note that although GARCH and stochastic volatility (SV) models are widely used to model risk and volatility in financial markets, their application to renewable energy stocks requires caution. Studies such as Mora-Valencia et al. (2020) show that renewable energy stocks tend to have higher volatility and more frequent VaR violations compared to conventional energy stocks. Our findings further reveal that renewable energy indices exhibit infinite fourth moments, implying that traditional autocorrelation-based methods and standard volatility modeling techniques may produce unreliable results. Stakeholders should therefore apply more robust methods when analyzing volatility clustering and risk patterns in renewable energy markets. |

Once again, thank you very much for very helpful and constructive comments and suggestions on the paper!

**References**

Almeida, D., Ferreira, P., Dionísio, A., Aslam, F. (2025). Exploring the connection between geopolitical risks and energy markets. *Energy Economics*, **141**, 108113.

Bouri, E., Hammoud, R., Kassm, C. A. (2023). The effect of oil implied volatility and geopolitical risk on GCC stock sectors under various market conditions. *Energy Economics*, **120**, 106617.

Gabriel, V., Dionísio, A., Almeida, D., and Ferreira, P.(2024). From wars to waves: geopolitical risks and environmental investment behaviour. *Post-Communist Economies*, **36(5)**, 631-658.

Gu, Z., & Ibragimov, R. (2018). The ‘‘Cubic Law of the Stock Returns’’ in emerging markets. [*Journal of Empirical Finance*](https://www.sciencedirect.com/journal/journal-of-empirical-finance), **46**, 182-190.

McNeil, A. J., Frey, R., Embrechts, P. (2015). *Quantitative Risk Management: Concepts, Techniques and Tools*, revised edition. Berlin: Springer

Müller, U. K., & Wang, Y. (2017). Fixed-*k* Asymptotic Inference About Tail Properties. *Journal of the American Statistical Association* **112**, 1334–1343.

Shahzad , U., Mohammed, K. S., Tiwari, S., Nakonieczny, J., Nesterowicz, R. (2023). Connectedness between geopolitical risk, financial instability indices and precious metals markets: Novel findings from Russia Ukraine conflict perspective. *Resources Policy*, **80**, 103190.

Toda, A. A., & Wang, Y. (2020). Efficient minimum distance estimation of Pareto exponent from top income shares. *Journal of Applied Econometrics* **36**, 228–243

[Quintos](https://www.jstor.org/action/doBasicSearch?Query=au%3A%22Carmela%20Quintos%22), C., Fan, Z., Phillips, P. (2001). Structural change tests in tail behaviour and the Asian crisis. [*The Review of Economic Studies*](https://www.jstor.org/journal/revieconstud), **68**, 633-663.

Zhang, W., He, X., Hamori, S. (2022). Volatility spillover and investment strategies among sustainability-related financial indexes: Evidence from the DCC-GARCH-based dynamic connectedness and DCC-GARCH t-copula approach. *International Review of Financial Analysis,* **83**, 102223.

# Appendix A

# ADF test results

| **Series** | **Model** | **TestStat** | **CritVal** | **Decision** |
| --- | --- | --- | --- | --- |
| DJUSEN | Intercept | -2.68130 | -3.43 | Do not reject |
| DJUSEN | Intercept+Trend | 0.0317 | -2.58 | Do not reject |
| DJUSEN | None | -2.7253 | -3.96 | Do not reject |
| DJUSEN Returns | Intercept | -41.2712 | -3.43 | Reject |
| DJUSEN Returns | Intercept+Trend | -41.2696 | -2.58 | Reject |
| DJUSEN Returns | None | -41.2678 | -3.96 | Reject |
| ECO | Intercept | -1.6844 | -3.43 | Do not reject |
| ECO | Intercept+Trend | -1.2866 | -2.58 | Do not reject |
| ECO | None | -1.9745 | -3.96 | Do not reject |
| ECO Returns | Intercept | -21.6415 | -3.43 | Reject |
| ECO Returns | Intercept+Trend | -21.6287 | -2.58 | Reject |
| ECO Returns | None | -21.6407 | -3.96 | Reject |
| ERIX | Intercept | -1.6843 | -3.43 | Do not reject |
| ERIX | Intercept+Trend | -0.6038 | -2.58 | Do not reject |
| ERIX | None | -1.5736 | -3.96 | Do not reject |
| ERIX Returns | Intercept | -27.7046 | -3.43 | Reject |
| ERIX Returns | Intercept+Trend | -27.7051 | -2.58 | Reject |
| ERIX Returns | None | -27.7085 | -3.96 | Reject |
| SPG | Intercept | -1.5352 | -3.43 | Do not reject |
| SPG | Intercept+Trend | -1.01153 | -2.58 | Do not reject |
| SPG | None | -1.9135 | -3.96 | Do not reject |
| SPG Returns | Intercept | -25.4371 | -3.43 | Reject |
| SPG Returns | Intercept+Trend | -25.4363 | -2.58 | Reject |
| SPG Returns | None | -25.4352 | -3.96 | Reject |
| SUN | Intercept | -1.9477 | -3.43 | Do not reject |
| SUN | Intercept+Trend | -1.2537 | -2.58 | Do not reject |
| SUN | None | -2.4404 | -3.96 | Do not reject |
| SUN Returns | Intercept | -47.2184 | -3.43 | Reject |
| SUN Returns | Intercept+Trend | -47.2228 | -2.58 | Reject |
| SUN Returns | None | -25.4352 | -3.96 | Reject |
