# Supplementary material for: Tail risk, large fluctuations and downfalls in renewable energy markets
Source: PLoS One. 2026 Jul 15;21(7):e0351106. doi: 10.1371/journal.pone.0351106 (PMC13372164; doi:10.1371/journal.pone.0351106)
Supplement: Attachment — Submitted filename: Review_to_Authors.docx [file pone.0351106.s007.docx]

**Tail Risk, Large Fluctuations and Downfalls in Renewable Energy Markets**

**FIRST READ-THROUGH**

**Main goals:** to analyze the heavy-tailedness properties of major renewable energy indices using robust econometric methods.; (ii) evaluate tail indices and their degree of heavy-tailedness using Hill’s estimator and LLRS regression with optimal rank shifts; (iii) provide implications for risk management, financial regulation, and investment strategies in renewable energy markets.

**Methods:** the study employs: (i) two tail index estimation techniques—Hill's estimator and the OLS-based LLRS regression with optimal shifts; (ii) a comparative analysis of tail indices for renewable energy indices (ECO, ERIX, SPG, SUN) and a conventional energy index (DJUSEN); (iii) statistical inference on tail index values to assess the existence of first, second, third, and fourth moments.

**Data:** The study uses daily data sourced from the Bloomberg terminal. The data covers the period from April 1, 2005, to July 29, 2022. The study analyses four renewable energy indices (WilderHill Clean Energy Index (ECO), European Renewable Energy Index (ERIX), S&P Global Clean Energy Index (SPG), and MAC Global Solar Energy Index (SUN)) and one conventional energy index (Dow Jones US Oil and Gas Index (DJUSEN).

**Main results:** (i) renewable energy indices reveal heavy-tailedness, with SPG showing the most pronounced heavy-tailedness; (ii) renewable energy indices exhibit finite first and second moments but infinite fourth moments, implying fat tails in their return distributions; (iii) gains and losses exhibit asymmetry, with downward movements showing heavier tails than upward movements; (iv) the SPG index demonstrates more pronounced heavy-tailedness than regional indices like ECO and ERIX.

The work aligns with the scope of *Plos One*.

While the paper references key foundational works, recent studies from the last five years are underrepresented (they only represent about 31% of the references) in critical areas like renewable energy market volatility. Thus, the literature review must be improved.

The experimental design aligns with the study’s objectives.

The figures and tables are appropriate.

The conclusions align with the findings and offer insights into renewable energy risk management and volatility, which are relevant to finance, sustainability, and energy market researchers. However, some policy-related claims could be more substantiated. The practical implications of the heavy-tailedness analysis for policymakers and investors could be better emphasized, perhaps with examples of how the findings could inform investment strategies or risk assessments.

The English language is appropriate and understandable.

**DETAILED REVIEW**

**TITLE**

The title accurately reflects the manuscript's core focus. It is precise and relevant to the manuscript's content.

**KEYWORDS**

The keywords chosen are relevant and accurately reflect the study's scope and methodology, aiding discoverability.

**ABSTRACT**

The abstract outlines the study’s goals and methods but should provide information about the analyzed period.

Furthermore, to maximize the article’s impact, it should highlight the contributions and implications of the study. The abstract's first sentence should be about the problem, motivation, or question. The second sentence should be about what we already know from the literature. In the third sentence, please say what you are doing and how; the following two sentences should be about what you find, followed by the significance of your findings. It is important to say what the question is; why this is an interesting and important question; how you go about it; why this is a persuasive modeling strategy; what you found; how this changes our view of the world (focusing on its novelty in relation to what we already know from the literature). People only spend one or two minutes reading an abstract; if they do not see the novelty within that time, they tend to ignore a paper. Our aim is to ensure that your paper gets cited after publication. Please consider highlighting the big ideas clearly and succinctly that is informative, novel, logical, and engaging. A reader will not be interested only in what you found but why you found it and how it helps solve a problem with more focus on practical implications/economic story rather than pure technical results; if you can add why you find something or why you have done something, they can have a bigger impact than just saying what the paper is doing.

Please consider improving it.

**INTRODUCTION**

The Introduction provides a clear rationale for studying heavy-tailedness in renewable energy markets. The text contextualizes the relevance of renewable energy within the broader framework of climate change and financial risk management. The objectives of the study are clearly stated. However, this section should be improved. Considering the following:

1. While climate change and renewable energy trends are discussed extensively, the connection to the specific research focus (tail risk in renewable energy markets) is introduced too late. Try to condense the discussion of general climate change trends and expand the link between heavy-tailedness and renewable energy finance. Try incorporating recent studies (e.g., heavy-tailedness in financial markets from the last five years).
2. The introduction mentions financial implications but does not adequately explain how this study fills specific financial risk management literature gaps. Please clarify how this study contributes to gaps in existing literature on renewable energy volatility and financial modeling.
3. There is limited discussion on why Hill’s estimator and LLRS regression were chosen.
4. Briefly mention the data sources and econometric methods to set the stage for subsequent sections.

**LITERATURE REVIEW**

The Literature Review provides a solid foundation by discussing: (i) the key trends in renewable energy markets, such as the relationships between oil prices, technology stocks, and renewable energy investments; (ii) foundational studies on heavy-tailedness and its relevance to financial and energy markets; (iii) important renewable energy indices and their role in understanding market performance; (v) adequately highlighting the volatility and risks associated with renewable energy markets, establishing the importance of analyzing tail risks.

However, this section should also be improved, considering the following:

1. It lacks recent studies. There is minimal coverage of studies published in the last five years, particularly those addressing renewable energy markets and tail risk in the context of recent global crises, such as the COVID-19 pandemic, geopolitical conflicts, or heightened policy uncertainty around climate change (e.g., Bouri, E., Hammoud, R., & Kassm, C. A. (2023). The effect of oil implied volatility and geopolitical risk on GCC stock sectors under various market conditions. *Energy Economics*, *120*, 106617. <https://doi.org/10.1016/J.ENECO.2023.106617>; Gabriel, V., Dionísio, A., Almeida, D., & Ferreira, P. (2024). From wars to waves: geopolitical risks and environmental investment behaviour. *Post-Communist Economies*, 1–28. <https://doi.org/10.1080/14631377.2024.2323763>; Mirza, N., Abubakr, M., Thu, T., Nguyen, H., Arfaoui, N., & Oliyide, J. A. (2023). Are sustainable investments interdependent ? The international evidence. *Economic Modelling*, *119*(September 2021), 106120. <https://doi.org/10.1016/j.econmod.2022.106120>; Shahzad, U., Mohammed, K. S., Tiwari, S., Nakonieczny, J., & Nesterowicz, R. (2023). Connectedness between geopolitical risk, financial instability indices and precious metals markets: Novel findings from Russia Ukraine conflict perspective. *Resources Policy*, *80*, 103190. <https://doi.org/10.1016/J.RESOURPOL.2022.103190>; Almeida, D., Ferreira, P., Dionísio, A., & Aslam, F. (2025). Exploring the connection between geopolitical risks and energy markets. *Energy Economics*, *141*, 108113. <https://doi.org/10.1016/J.ENECO.2024.108113>; Zhang, W., He, X., & Hamori, S. (2022). Volatility spillover and investment strategies among sustainability-related financial indexes: Evidence from the DCC-GARCH-based dynamic connectedness and DCC-GARCH t-copula approach. *International Review of Financial Analysis*, *83*, 102223. <https://doi.org/10.1016/J.IRFA.2022.102223>). Furthermore, studies addressing the rapidly evolving nature of renewable energy markets, especially advancements in econometric modeling for volatility and tail risk, are notably absent.
2. There is a heavy focus on historical context. While historical studies provide valuable background, they dominate the discussion, leaving limited room for recent advancements or emerging topics like renewable energy market performance during global disruptions or the role of artificial intelligence in market forecasting. Expand the focus beyond the relationships between oil prices, technology stocks, and renewable energy, as these topics have been explored extensively in earlier works. Instead, emphasize more nuanced relationships, such as the interplay between renewable energy policy changes and market volatility. Introduce recent themes like green energy transitions and carbon trading and their implications for renewable energy stocks.
3. There is a limited connection to heavy-tailedness in energy markets. Although heavy-tailedness is a central theme of the paper, the literature review does not delve deeply into recent works that explore heavy-tailed distributions in renewable energy or broader financial markets (e.g., studies applying advanced econometric techniques, such as machine learning approaches, to model heavy-tailed risks are missing).

**METHODOLOGY AND DATA**

The section clearly explains Hill’s estimator and LLRS regression with optimal rank shifts, including their mathematical foundations, which are well explained in the Methodology and Data section. The data sources and indices analyzed are well-documented. However, this section needs improvements, considering the following:

1. The assumptions are not entirely justified. For example, 5% and 10% truncation levels for extreme observations are mentioned but not adequately justified. Justify the choice of truncation levels.
2. There is no sensitivity analysis, i.e., no discussion of how results might vary with different truncation levels or alternate methods.
3. Was the stationarity of the data analyzed? Was the data stationary? Please provide the results of stationarity tests.
4. Data limitations. The data used was sourced from the Bloomberg terminal. However, its restricted availability may not follow the *Plos One* data-sharing policy, and no alternative public data sources are suggested. Address data-sharing limitations and suggest alternative publicly available datasets.
5. Explain the analyzed period (April 1, 2005, to July 29, 2022). Provide a strong explanation for the end date (July 29, 2022) or update the results (we are at the beginning of 2025; it does not seem adequate to accept studies with data only until 2022).

**RESULTS**

This section presents the results systematically, with clear tables summarizing key findings. The comparison of renewable and conventional energy indices is insightful. Gain/loss asymmetry and its implications for financial modeling are briefly discussed. However, this section should also be improved, considering:

1. The section emphasizes tail index estimates but provides limited interpretation of gain/loss asymmetry or cross-index comparisons. Please expand the discussion on gain/loss asymmetry and its implications for risk management.
2. The results' robustness under different methodological assumptions (e.g., alternate truncation levels) is not discussed. Try to include robustness checks to ensure findings are not highly sensitive to methodological choices.
3. It is referred to as “This study's initialisation window is 100 days”. Please explain the selection of this window. Why was it selected? Were the results robust in different windows (e.g., 250, 500, 1000 observations)?

**DISCUSSION**

This section highlights the study's implications for risk management, financial institutions, and policymakers. However:

1. It briefly contrasts findings with prior literature, i.e., insufficient discussion of how the findings align with or challenge existing studies, especially recent ones.
2. The discussion of policy relevance is general and lacks specific recommendations. Expand the policy discussion to include concrete recommendations for risk management or financial regulation.
3. Furthermore, the potential for the findings to inform renewable energy portfolio strategies or regulatory frameworks is not fully explored. Please explore practical applications of the findings for portfolio diversification, investment strategies, or renewable energy incentives.

**CONCLUSION**

The Conclusion summarizes the study’s findings and reiterates their relevance to renewable energy markets and financial risk management. It restates the study's key contributions. However, the conclusions make broad claims about the relevance of tail risk without adequately referencing specific findings. Furthermore, the implications of data-sharing restrictions on replicability are not addressed, nor are there other limitations of the study. Please address these issues.
