# Supplementary material for: Tail risk, large fluctuations and downfalls in renewable energy markets
Source: PLoS One. 2026 Jul 15;21(7):e0351106. doi: 10.1371/journal.pone.0351106 (PMC13372164; doi:10.1371/journal.pone.0351106)
Supplement: Attachment — Submitted filename: z_Response to Reviewer (R2)_revised_final.docx [file pone.0351106.s010.docx]

**Reviewer #1 Comments and Author Response**

Thank you for your thoughtful and detailed revision of the manuscript. You have addressed all comments comprehensively, and the overall quality of the manuscript has improved significantly. Yet, there is a minor issue that needs your attention.

| **Comment** | **Response** |
| --- | --- |
| 1. Please, remove the sentence “We thank the reviewer for highlighting the importance of the analysis of heavy-tailedness of indices from emerging markets, given their growing role in the renewable energy sector. A study in this direction will be the focus of future research.” (page 15, lines 397-399)” from the main body of the manuscript.   Once corrected, I believe the manuscript will be ready for publication. All the best! | Thank you for pointing this out.  The indicated sentence has been removed from the main body of the manuscript as suggested. |

**Reviewer #3 Comments and Author Response**

Overall Assessment: This paper presents a timely and relevant empirical investigation into the heavy-tailedness and tail risk properties of major renewable energy equity indices, comparing them with a conventional energy benchmark. The topic is of significant importance to investors, portfolio managers, and policymakers given the rapid growth and integration of renewable assets into global financial markets. The study employs robust methodologies, specifically the log-log rank-size (LLRS) regression with an optimal shift parameter (-1/2) and Hill's estimator, to analyze daily return data from 2005 to 2025. The core finding—that renewable energy indices exhibit heavy tails but are generally less heavy-tailed than the conventional energy index, with finite first and second moments but likely infinite higher moments—is a valuable contribution to the literature on clean energy finance and financial risk management. The paper is generally well-structured, the literature review is comprehensive, and the methodological choice is justified based on recent advances in tail index estimation. The application of a recursive window approach to observe the dynamic evolution of tail risk through crises (2008 GFC, 2020 COVID-19 shock) is a notable strength. However, the manuscript, in its current form, requires substantial revisions before it can be considered for publication. The major concerns revolve around the depth of methodological justification, the interpretation and economic contextualization of results, robustness checks, and the overall narrative flow. Several analytical claims require more rigorous statistical support and clearer exposition. The following report details major and minor concerns that must be addressed to strengthen the manuscript significantly.

| **Comment** | **Response** |
| --- | --- |
| **Major Concerns** | |
| **1.1 Methodological Robustness and Justification** | |
| While the authors correctly highlight the advantages of the LLRS regression with a -1/2 shift (Gabaix & Ibragimov, 2011) over the standard Hill estimator in the presence of dependence and small-sample bias, the application and validation of this method within the specific context of renewable energy returns need deeper exposition. | |
| 1. Threshold Selection (k or n): The choice of the tail truncation level (5% and 10%) is stated to be supported by Hill-type plots (Fig 2) and references to prior literature. However, Figure 2, as described, shows stability and overlapping CIs but does not convincingly demonstrate a clear "flat" region (a stability plateau) which is the standard diagnostic for appropriate threshold selection in tail index estimation. The authors should provide a more formal analysis for threshold selection. They could employ widely used diagnostic plots like the Hill plot, the alt-plot, or the mean excess plot for the absolute returns of each index. A discussion on how the chosen thresholds (250-500+ observations) are sufficiently large for asymptotic approximations while not encroaching into the central part of the distribution is necessary. Sensitivity analysis using a range of thresholds (e.g., from 2.5% to 15% in finer increments) and reporting how the tail index estimates and their standard errors evolve would greatly enhance robustness. | We thank the reviewer for this constructive comment and have strengthened the discussion of threshold selection and robustness accordingly.  The truncation levels of 5% and 10% are not intended to identify a unique optimal threshold, but rather to ensure robust inference across empirically relevant tail sizes commonly used in the heavy-tails literature. These thresholds correspond to approximately 250-500 extreme observations per series, striking a balance between asymptotic reliability and avoidance of the central part of the return distribution, consistent with prior empirical studies.  To further assess sensitivity to threshold choice, Figure 2 reports LLRS truncation-stability results over an expanded grid of tail fractions (2.5%-15%). While a perfectly flat plateau is uncommon in finite samples with dependent returns, the estimates evolve smoothly and display substantial overlap of 95% CIs, suggesting that differences across thresholds are not statistically meaningful.  Additional robustness diagnostics are reported in the Appendix (Table A2), documenting how tail index estimates, CIs, and effective tail sample sizes vary with truncation and across raw and GARCH-filtered returns. These results confirm that the main conclusions regarding tail heaviness and moment (in)finiteness are not driven by a particular threshold choice.  The manuscript has been revised to clarify these points in Section 4.2 paragraph 3, with supporting evidence provided in Figure 2 and the Appendix. |
| 2. Dependence and Volatility Clustering: The authors rightly note that financial returns exhibit volatility clustering and that the LLRS method with the optimal shift is robust to dependence. However, they do not demonstrate this property for their dataset. A key question is whether the extreme returns used in the estimation (the upper 5%/10% of absolute returns) themselves exhibit significant temporal dependence. Simple tests for autocorrelation in the extremes or in squared extremes could be presented. If significant dependence is found, the authors should discuss its potential impact even on the "robust" LLRS method and whether techniques like block bootstrapping for standard errors (as an additional check) were considered and why they were or were not employed. | We thank the reviewer for this important comment concerning the role of dependence and volatility clustering in tail index estimation.  To address this issue, we explicitly document serial dependence and volatility clustering in the return series using Ljung-Box diagnostics applied to absolute and squared returns (Appendix Table A1). We then estimate a GARCH(1,1) model and compute standardized residuals to mitigate conditional heteroskedasticity (Appendix Fig. A1). Tail index estimates are subsequently re-computed using both raw returns and GARCH-filtered innovations as a robustness check (Appendix Table A2).  Regarding inference under dependence, the shifted LLRS estimator with corrected SE proposed by Gabaix & Ibragimov (2011) differs from earlier OLS-based implementations in that the analytical SE explicitly account for dependence in the ordered tail observations used in estimation. This dependence arises naturally from the ranking procedure and persists even when the underlying return sequence is i.i.d. In addition, the LLRS approach has been shown to exhibit favorable finite-sample properties under broader departures from i.i.d. assumptions in the full return sample, including volatility clustering and GARCH-type dynamics typical of financial time series, as discussed in Section 3.3 and the related literature.  Given these theoretical robustness properties, and the empirical stability of tail index estimates across raw and GARCH-filtered returns, we do not rely on additional block bootstrapping procedures. The consistency of results indicates that the main conclusions are not driven by residual temporal dependence in the extremes.  These clarifications are reflected in Section 3.3 paragraph 4, with supporting diagnostics reported in the Appendix. |
| 3. Goodness-of-Fit to Power Law: The entire analysis hinges on the assumption that the tails of the return distributions follow a power law (Pareto-type tail). The authors should provide more formal goodness-of-fit tests for the power-law hypothesis. Visual Pareto quantile plots (QQ-plots against the exponential distribution) for the log-transformed exceedances are a standard tool. Statistical tests, such as the Kolmogorov-Smirnov test adapted for Pareto distributions or the Clauset-Shalizi-Newman test, should be applied and reported. This is crucial because if the tails deviate significantly from a power law, the interpretation of the tail index ζ loses its precise meaning regarding the finiteness of moments. | We thank the reviewer for raising the important question of whether the tail behavior of returns is adequately described by a power-law specification.  In the revised manuscript, we expand Section 2.3 to provide additional theoretical and empirical context for heavy-tailed modeling frameworks in economics and finance. In particular, we discuss contributions by (Gabaix, 1999) and (Gabaix et al., 2003, 2006), which link the emergence of power-law tails in financial returns to economic mechanisms such as the Zipf distribution of firm and investor sizes and their trading impact. These insights provide a structural rationale consistent with empirically observed heavy-tailed return behavior.  At the same time, we clarify that our analysis does not interpret tail index estimation as a test of a literal Pareto data-generating process. Rather, following Gabaix & Ibragimov (2011) and related literature, the shifted LLRS estimator with corrected SE is employed to characterize effective tail thickness and the implied finiteness of moments under a broad class of heavy-tailed distributions.  As discussed in Section 3.3, LLRS inference with optimal rank shift and corrected SE exhibits robustness to deviations from exact Pareto behavior, including slowly varying components and dependence structures typical of financial return series (e.g., volatility clustering and GARCH-type dynamics). In this framework, the estimated tail index $\zeta$ retains its interpretation in terms of moment implications even when the underlying distribution departs from a strict Pareto specification.  We also note that classical goodness-of-fit procedures such as Kolmogorov-Smirnov tests are derived under i.i.d. asymptotics and may exhibit limited power in finite samples, particularly in distinguishing between pure power-law and alternative heavy-tailed specifications. Given that our objective is robust tail thickness inference rather than validation of an exact Pareto law, we interpret $\zeta$ as a measure of effective tail heaviness and moment implications rather than as evidence of a strict Pareto data-generating process.  These clarifications are reflected in the revised Sections 2.3 and 3.3. |
| **1.2. Data and Sample Representativeness** | |
| 4. Index Composition and Heterogeneity: The analysis treats indices (ECO, ERIX, SPG, SUN, DJUSEN) as monolithic entities. However, these indices have vastly different compositions. ECO is US-focused and equal-weighted, SPG is global and presumably market-cap weighted, SUN is sector-specific (solar). DJUSEN is a US oil & gas index. This heterogeneity is a double-edged sword: it provides diversity but complicates direct comparison. The finding that SPG (global) has the smallest tail index (heaviest tail) is attributed to its inclusion of emerging market companies. This is a plausible hypothesis but remains untested. The authors should attempt to control for or discuss this factor more deeply. Could the heavier tail of SPG be due to its weighting scheme, its sectoral breadth (beyond solar), or indeed its geographic exposure? A correlation analysis between the tail index estimates and index characteristics (e.g., average market cap, geographic concentration Herfindahl index, sector diversity) across a broader set of indices (if data were available) would be insightful. As it stands, the conclusion is suggestive but not conclusive. | We thank the reviewer for this insightful comment regarding index composition and cross-index heterogeneity.  We agree that the renewable energy indices considered in the analysis differ substantially in terms of geographic exposure, sectoral focus, and weighting methodology, which complicates direct causal comparisons across indices. This heterogeneity is now explicitly acknowledged and discussed in Section 2.2 last paragraph, where we emphasize that the use of alternative index constructions enhances external relevance but limits attribution of cross-index differences in tail behavior to any single structural characteristic.  Accordingly, differences in estimated tail indices, such as the relatively heavier tails observed for the global SPGCE index, are interpreted as descriptive and suggestive, rather than as conclusive evidence driven by geographic exposure, sectoral breadth, or weighting schemes. This interpretive caution is further reinforced in Section 4.2 paragraph 4, where cross-index comparisons are framed in terms of robustness and CI-based inference rather than causal identification.  A formal decomposition linking tail behavior to specific index characteristics (e.g., market capitalization, geographic concentration, or sectoral diversity) would require a broader cross-section of indices and consistent constituent-level data, which lies beyond the scope of the present study. The manuscript has been revised accordingly to clarify these points. |
| 5. Time Period and Structural Breaks: The sample covers 2005-2025, a period encompassing multiple regimes: pre-GFC boom, GFC, post-GFC recovery, the shale revolution (impacting DJUSEN), the COVID-19 crash, and the recent energy crisis. The recursive window analysis in Section 4.2 is excellent for showing dynamics but the full-sample tail index estimates presented in Table 3 may be an average over these heterogeneous periods. The authors should consider conducting a formal test for structural breaks in the tail index itself, using methods such as the Quintos, Fan, and Phillips (2001) test or similar approaches referenced in their literature review (e.g., Xing & Ibragimov, 2023). Identifying breakpoints (e.g., around 2008, 2020) and reporting sub-period tail indices would provide a richer narrative. For instance, is the "less heavy-tailed" result for renewables stable across all sub-periods, or is it a feature of the post-GFC era? | We thank the reviewer for this insightful comment regarding potential regime heterogeneity and structural breaks over the long sample period.  We agree that the 2005-2025 sample encompasses multiple distinct market regimes, including the GFC, the COVID-19 shock, and recent energy market disruptions. Rather than relying solely on full-sample tail index estimates, the manuscript explicitly addresses this concern through a recursive expanding-window tail index analysis presented in Section 4.2 and Figure 3.  Figure 3 shows pronounced declines in tail indices during major global stress episodes, most notably during the 2007-2008 GFC and, to a lesser extent, during the COVID-19 period, followed by stabilization in subsequent years. This dynamic evidence demonstrates that tail behavior varies systematically across regimes and that crisis periods materially influence tail risk in both renewable and conventional energy markets.  Accordingly, the full-sample tail index estimates reported in Table 3 are interpreted as benchmark summaries rather than averages masking structural instability. The recursive analysis provides complementary time-varying evidence showing that the relative tail behavior of renewable and conventional energy indices evolves with market conditions, while remaining broadly comparable outside crisis periods, with inference consistently based on CIs rather than point estimates alone.  Formal breakpoint tests for tail indices and sub-sample estimation would constitute a valuable extension of the analysis. However, such tests require additional assumptions on breakpoint timing and window selection and would substantially expand the scope of the paper. Given that the recursive results already capture regime-dependent tail dynamics in a transparent and non-parametric manner, we believe the current approach adequately addresses the reviewer’s concern.  The manuscript has been clarified accordingly in Section 4.2, with the interpretation of full-sample estimates explicitly framed in light of the recursive evidence. |
| 6. Benchmark Choice and Omitted Comparisons: Using DJUSEN (US Oil & Gas) as the sole conventional energy benchmark is reasonable but narrow. The risk profile of a global integrated energy index (e.g., MSCI World Energy) might differ. More critically, to truly assess the "alternative" nature of renewables, a comparison with broad equity market indices (e.g., S&P 500, MSCI World) is almost mandatory. The claim that renewables are a "relatively lower-risk investment alternative" (Abstract) is made relative to dirty energy. How do their tail risks compare to the overall equity market? This is a key question for portfolio managers considering renewables for diversification. Adding this comparison would significantly broaden the paper's impact. | We thank the reviewer for this constructive comment regarding benchmark selection and the broader interpretation of renewable energy assets as investment alternatives.  The choice of DJUSEN as a benchmark is deliberate and aligned with the paper’s core objective, which is to compare tail risk properties of renewable energy equities against conventional fossil fuel-based energy markets. DJUSEN provides a clear and economically meaningful reference point for this comparison, as it represents U.S. oil and gas firms operating under markedly different production technologies, regulatory environments, and exposure to commodity price risk than renewable energy companies.  We agree that alternative benchmarks, such as global integrated energy indices or broad equity market indices (e.g., MSCI World or S&P 500), could provide additional perspectives, particularly from a portfolio allocation standpoint. However, extending the analysis to such benchmarks would substantially broaden the scope of the paper beyond its primary focus on energy market tail risk and would require a parallel treatment of heterogeneous asset classes with different return-generating mechanisms.  Accordingly, the statement that renewable energy equities exhibit relatively lower tail risk is explicitly framed in the manuscript as a comparison with conventional energy markets, rather than with the overall equity market, with inference based on CIs rather than point estimates alone. This interpretation is clarified in the Abstract and reiterated in Section 4.2, where conclusions are drawn relative to the DJUSEN benchmark and within the energy sector context.  A systematic comparison between renewable energy equities and broad market indices, while highly relevant for portfolio diversification analysis, is therefore left for future research. We believe that maintaining a focused benchmark choice enhances internal coherence and interpretability of the tail risk results presented in this study. |
| **1.3** **Interpretation of Findings and Statistical Significance** | |
| 7. The "Less Heavy-Tailed" Conclusion: This is a central finding. However, the authors note that the confidence intervals for the tail indices of renewable and conventional indices overlap, and thus the differences are "not statistically significant" (pp. 19, 29). This is a critical caveat that is underemphasized. The abstract and conclusion sections (pp. 1, 28-29) strongly state that renewables are less heavy-tailed, while the results section correctly highlights the lack of statistical significance. This creates a contradiction. The narrative must be consistently cautious. The paper should clearly state: Point estimates suggest renewable indices (except SPG) have larger tail indices than DJUSEN, but these differences are not statistically distinguishable at conventional levels. The economic implication—that investors cannot reliably conclude that renewables have significantly lighter tails than oil/gas based on this data—must be front and center. The discussion on pp. 27-28 ("aligns with existing literature that renewable energy assets are less riskier") should be tempered accordingly. | We thank the reviewer for emphasizing the importance of consistent interpretation.  In the revised manuscript, all conclusions regarding relative tail behavior are based strictly on CI-based inference rather than point estimates. As shown in Section 4.2 and emphasized explicitly in Section 5 paragraph 9, the results do not support statistically significant differences in tail heaviness between renewable and conventional energy indices. Accordingly, any apparent differences are interpreted as descriptive tendencies rather than statistically established dominance.  Section 4.2, Section 5 (Discussion) and Section 6 (Conclusion) have been carefully aligned with this CI-based framework to avoid overstatement and to ensure a consistently cautious narrative, including explicit clarification in the Abstract that point-estimate differences are not statistically distinguishable at conventional CIs. |
| 8. Inference on Higher Moments: The discussion about infinite third and fourth moments (pp. 20, 26-27, 29) is based on hypothesis tests (ζ=3, ζ=4). The authors must report the actual p-values for these tests, not just rejection/non-rejection statements. More importantly, they must discuss the economic and practical significance of an "infinite" third or fourth moment. In finite samples, what we observe is "very large" skewness/kurtosis, not literal infinity. The key takeaway is that higher-moment estimates are extremely unstable and unreliable. The warning about the limitations of mean-variance-skewness (MVS) models (p. 27) is valid, but it should be framed as: "Given the extremely high and unstable estimates of higher moments, models relying on precise estimates of skewness and kurtosis are likely to be unreliable and should be used with extreme caution," rather than a definitive statement about infinities. | We thank the reviewer for this important comment regarding the interpretation of higher-order moments under heavy-tailed return distributions.  In the revised manuscript, inference on third and fourth moments is conducted strictly within a CI-based framework rather than relying on point estimates or binary hypothesis testing. As discussed in Section 5 paragraphs 4 and 7 and reiterated in Section 6 paragraphs 3 and 6, the reported CIs often cross or include the thresholds associated with finite third and fourth moments, implying that higher-order moments cannot be inferred with statistical reliability. Because inference is implemented through CIs derived from the asymptotic distribution of the tail index estimator, separate p-value reporting is not required, as statistical significance is fully characterized by the CI relative to the relevant moment threshold ($\zeta=3$, $\zeta=4$).  Importantly, the manuscript does not interpret these findings as evidence of literal infinities in finite samples. Rather, they indicate extremely large and unstable higher-moment behavior, rendering skewness- and kurtosis-based measures unreliable in practice. This interpretation is now stated explicitly in the discussion, where we emphasize that models relying on precise estimation of higher moments, such as MVS portfolio frameworks, should be applied with considerable caution in renewable energy markets.  Accordingly, the economic implication emphasized in the revised manuscript is that while MV-based methods remain admissible, higher-moment-based inference and optimization are unlikely to be robust in the presence of empirically heavy and unstable tails. This interpretation is applied consistently across Section 5 (Discussion) and Section 6 (Conclusion). |
| 9. Gain/Loss Asymmetry Analysis (Tables 4 & 5): The analysis of left vs. right tails is valuable. However, the interpretation is muddled. The authors find mixed patterns (e.g., ECO heavier left at 5%, heavier right at 10%) and correctly note that CIs largely overlap. They then state, "Overall, the results... suggest that significant downfalls... tend to be somewhat more pronounced than their significant upward moves" (p. 22). This summary seems to lean on the point estimates for SPG, SUN, DJUSEN and downplays the mixed/overlapping results for ECO and ERIX. A more balanced conclusion is needed: there is weak and inconsistent evidence of asymmetry across these indices, with no statistically significant left-right difference for most. This should be linked to the literature on asymmetric volatility in renewables - is the asymmetry in volatility mirrored in the tail indices? A brief discussion is warranted. | We thank the reviewer for this careful reading of the gain-loss asymmetry results.  In the revised manuscript, the interpretation of left- and right-tail estimates is explicitly grounded in CI-based inference rather than point estimates. As reported in Section 4.2 paragraph 10, while point estimates of right- and left-tail indices vary across indices and truncation levels, formal CI comparisons indicate that these differences are generally not statistically distinguishable at conventional significance levels. Accordingly, any apparent asymmetries are interpreted as directional tendencies rather than statistically established dominance of downside or upside tail risk.  The discussion has been revised to emphasize that evidence of asymmetry is weak and inconsistent across indices, with mixed patterns observed for ECO and ERIX and no robust left-right dominance supported by CI-based inference. This ensures a balanced interpretation that does not over-weight results for specific indices, and the previously stronger summary statement has been tempered to reflect the absence of statistically significant asymmetry.  A brief contextual link is also provided in Section 4.2 paragraph 10 to the broader literature on asymmetric volatility in renewable energy markets, noting that while volatility asymmetry is well documented, such asymmetries do not necessarily translate into statistically distinct tail decay rates. This distinction further motivates the cautious, CI-based interpretation adopted throughout the manuscript. |
| **1.4. The Recursive Window Analysis (Section 4.2)** | |
| 10. Interpretation of Persistence: The description of the recursive method is good. However, the interpretation of the "stabilization" of tail indices post-2008 needs refinement. The authors state that extreme values from the crisis persist in the window, maintaining tail heaviness. This is technically true, but it raises a question: does the stabilized tail index (e.g., ~3 for ECO) represent the new steady-state tail behavior of the market, or is it an artifact of the permanently embedded 2008 crisis data? A rolling window analysis (with a fixed window size, e.g., 1000 days) could complement the recursive analysis. A rolling window would show whether the tail index eventually reverts to a pre-crisis level once the crisis data rolls out of the sample, or if the change is permanent. This would offer a sharper view of whether crises permanently alter the tail risk structure. | We thank the reviewer for this insightful comment on the interpretation of persistence in the recursive tail index estimates.  The recursive expanding-window methodology is designed to capture the cumulative impact of extreme events, and the manuscript explicitly acknowledges that major crisis observations remain embedded in subsequent estimation windows. This feature is described in Section 4.2 paragraph 11, where we note that once extreme events occur, their effects carry over into later recursive estimates.  Accordingly, the post-2008 stabilization of tail indices is interpreted descriptively as persistent tail risk rather than as evidence of a new steady-state regime or a structural break. This interpretation is reinforced in Section 5 paragraph 11, where tail risk is characterized as persistent and state-dependent. The analysis does not claim reversion or permanent structural change, and conclusions are framed to reflect the methodological properties of the recursive approach rather than causal regime shifts.  While a fixed-length rolling window analysis could provide complementary evidence on whether tail indices revert once crisis observations exit the sample, implementing such an extension would substantially expand the empirical scope of the paper. We therefore focus on the recursive framework as a transparent and cumulative measure of tail risk evolution, while acknowledging that alternative window schemes may yield additional insights for future research. |
| 11. The 2020 Event: The minimal reaction of tail indices (except DJUSEN) to the COVID-19 shock is an interesting finding. The authors provide two interpretations (p. 24). They should add a third, methodological, possibility: the 2008crisis was so extreme that it dominates the tail of the distribution. The 2020 shock, while severe, may not have generated observations that were more extreme than the top 5% or 10% already occupied by 2008 data, thus leaving the tail index estimate unchanged. This speaks to the "persistence" feature they described. Analyzing the entry and exit of specific extreme dates into/from the top *n* observations in the recursive window could visually demonstrate this. | We thank the reviewer for highlighting this additional methodological interpretation of the muted tail response during the COVID-19 period.  As discussed in Section 4.2 paragraphs. 12-13, the GFC generates substantially more extreme observations than later stress episodes, leading to a pronounced and persistent decline in recursive tail indices. The comparatively milder tail response during the COVID-19 period is therefore consistent with the recursive framework, in which earlier and more extreme crisis observations continue to dominate the upper tail of the distribution.  This interpretation aligns with the discussion in Section 5 paragraph 11, where persistence is emphasized as an inherent feature of recursive tail estimation. The manuscript thus interprets the limited COVID-19 response as reflecting relative extremity rather than the absence of stress effects, without invoking additional structural assumptions.  While a detailed tracking of the entry and exit of individual extreme observations in the recursive tail set could provide additional visual illustration of this mechanism, such an extension would substantially expand the empirical presentation. We therefore retain the recursive framework interpretation while acknowledging this as a valuable avenue for future work. |
| **1.5. Discussion and Implications Section** | |
| 12. Connecting Tail Indices to Practical Risk Metrics: Section 5 begins well by linking heavy-tailedness to model robustness. However, it stops short of demonstrating the quantitative implications for risk management. The authors should include a small, illustrative calculation. For example, using the estimated tail index ζ for ECO and a parametric (Pareto) tail model, calculate the 1-day 99% Value at Risk (VaR) and Expected Shortfall (ES). Then, compare this to the VaR/ES estimated under a false assumption of normality (using sample mean and variance). This would vividly show how underestimating tail heaviness leads to a severe underestimation of risk capital. This practical demonstration would bridge the gap between the econometric results and the paper's stated audience of risk managers and investors. | We thank the reviewer for this constructive suggestion aimed at strengthening the practical interpretation of the tail index results.  The revised manuscript addresses this point conceptually rather than through additional parametric VaR or ES calculations. As discussed in Section 5 paragraphs 4 and 5, the analysis explicitly links tail index estimates to the theoretical validity of common risk measures. In particular, CI-based inference supports the existence of finite first and second moments across all indices, implying that both VaR and ES are theoretically well defined, while simultaneously highlighting the limitations of models that rely on higher moments.  The manuscript deliberately avoids illustrative parametric VaR or ES calculations based on a specific distributional assumption, as such exercises would require imposing a particular tail model (e.g., Pareto versus lognormal), which the paper shows is not uniformly supported across indices and truncation levels. Instead, the contribution of the paper lies in providing statistically justified inference on tail behavior itself, which underpins the reliability or fragility of downstream risk measures.  This emphasis is now clearly articulated in Section 5, where the implications of heavy-tailedness for risk measurement and model robustness are discussed directly in the context of VaR, ES, and related tools. |
| 13. Portfolio Implications: The discussion on portfolio theory (pp. 26-27) is good but could be more specific. Given the finding of finite variance, mean-variance analysis is theoretically justified. But what does the potential infinite higher moments imply for popular empirical practices? For instance, many portfolio optimizers use historical return samples, which will produce finite but wildly unstable estimates of skewness and kurtosis. The practical advice should be: "While mean-variance frameworks are applicable, attempting to optimize portfolios based on historical higher-moment estimates (e.g., for skewness-seeking strategies) in renewable energy markets is likely to lead to erratic and unreliable portfolio weights due to the extreme estimation error." This is a stronger, more practical warning than just stating MVS may be "limited." | We thank the reviewer for encouraging a clearer articulation of the practical portfolio implications of the results.  In the revised manuscript, the discussion of portfolio theory has been sharpened to emphasize practical estimation issues rather than abstract moment existence. As discussed in Section 5 paragraph 7, while CI-based inference supports the applicability of MV analysis, it does not provide reliable support for finite third moments. Accordingly, the manuscript now states explicitly that portfolio strategies relying on skewness or higher-moment optimization more generally are likely to produce highly unstable and erratic portfolio weights in practice due to extreme estimation uncertainty.  Importantly, the discussion clarifies that this result should not be interpreted as evidence of literal divergence in finite samples, but rather as reflecting extremely large and unstable higher-moment behavior that undermines the reliability of skewness- and kurtosis-based optimization. This interpretation is reinforced by empirical evidence from Jondeau & Rockinger (2006), cited directly in the revised discussion.  These revisions strengthen the practical guidance provided to investors and risk managers, while remaining fully consistent with the CI-based inference framework adopted throughout the paper. |
| 14. Overstatement of Certain Conclusions: Some statements are too broad. E.g., p. 28: "provides investors with a further incentive to substitute their holdings in dirty energy assets with renewable energy assets." This is an investment recommendation that goes beyond the scope of the risk analysis presented. The paper shows comparable or possibly slightly milder tail risk, but does not analyze expected returns, correlations, or other diversification benefits. The conclusion should stick to the risk dimension: "From a tail risk perspective, our findings do not present an obstacle and may provide mild support for considering renewable energy assets as a substitute for conventional energy holdings within a diversified portfolio." | We thank the reviewer for highlighting the need for careful wording of the investment implications.  In the revised manuscript, statements suggesting that renewable energy assets are inherently ‘lower risk’ or that investors should substitute conventional energy holdings have been removed or substantially tempered. As emphasized in Section 5 paragraph 9, CI-based inference does not support statistically significant differences in tail heaviness between renewable and conventional energy assets.  The conclusions are now framed strictly in terms of tail risk characteristics rather than investment recommendations. Specifically, the manuscript states that renewable energy assets remain exposed to substantial tail risk, particularly during periods of systemic stress, and that claims of uniformly lower risk should be avoided. Where comparative language is retained, it is explicitly framed as descriptive and conditional on CI-based inference, rather than as a prescriptive investment recommendation. The discussion in Section 6 has been aligned accordingly to ensure that the conclusions remain confined to the risk dimension analyzed in the paper and do not extend to expected returns, diversification benefits, or portfolio dominance.  These revisions ensure consistency between the empirical results and the interpretation, and avoid overstatement beyond the scope of the analysis. |

| **Comment** | **Response** |
| --- | --- |
| **Minor Concerns** | |
| **2.1 Clarity and Exposition** | |
| 1. The paper is lengthy and can be verbose. Some sections, particularly the literature review, could be tightened without losing essential information. The introduction effectively sets the stage, but the transition to the paper's specific contributions could be sharper. | We thank the reviewer for this comment.  In revising the manuscript, we carefully edited all sections to improve clarity, coherence, and conciseness, and to ensure smoother transitions from the general literature to the paper’s specific objectives and contributions. These refinements were implemented throughout the manuscript in response to the full set of reviewer comments. |
| 2. Abstract: The abstract states the study uses data "from 2005 to 2025." As of the current date (presumably 2024 or early 2025), this is a future date. It should read "through [last available date]" or "covering the period from 2005 to[specific date in 2024/2025]." | We thank the reviewer for noting this point.  The Abstract has been revised to state the exact sample period, now reported as April 2005 to February 2025, consistent with the data description in Section 3.4. |
| 3. Defining "Heavy-Tailedness": The definition in Section 2.3 is technically correct but could be made more accessible early on. A simple intuitive explanation (e.g., "higher probability of extreme crashes and booms compared to the normal distribution") in the introduction would help a broader audience. | We thank the reviewer for this helpful suggestion.  To improve accessibility, we have added a brief intuitive explanation of heavy-tailedness in Section 1 paragraph 3, clarifying that heavy-tailed distributions imply a higher probability of extreme crashes and booms relative to the normal distribution.  The formal definition and econometric framework remain in Section 2.3. |
| **2.2 Figures and Tables** | |
| 4. Figure 1 (Return Plots): This figure is essential but as described, it seems to be a simple time-series plot. Consider adding horizontal lines marking the +/− thresholds that correspond to the 5% and 10% tails. This would visually connect the return series to the extreme events used in estimation. | We thank the reviewer for the suggestion.  The tail thresholds employed in the analysis are quantile-based and index-specific, defined in terms of the largest 5% and 10% of absolute returns rather than fixed return levels. As such, adding horizontal cutoff lines to the return plots could be misleading. Figure 1 is therefore retained as a descriptive visualization of price dynamics, volatility clustering, and major stress episodes, while the connection to tail estimation is made explicitly through the truncation-stability analysis in Figure 2 and the corresponding tables (in the main manuscript and Appendix). |
| 5. Tables 3, 4, 5: These are dense. Consider using a format that highlights key comparisons. For example, in Table 3, use bold or shading to indicate the smallest tail index (SPG) and the largest (ECO at 5%). A summary table presenting the moment existence conclusions (Finite/Infinite for 1st, 2nd, 3rd, 4th) for each index based on both methods would be extremely helpful for the reader. | We thank the reviewer for this helpful suggestion.  To improve clarity, Tables 3 to 5 have been streamlined and reformatted to reduce visual density. In addition, we now provide Appendix Table A4, which summarizes the moment existence implications (mean, variance, skewness, kurtosis) implied by CI-based tail index inference for both the LLRS regression and Hill’s estimator. This summary table allows readers to quickly assess the implications of Table 3 without relying on point estimates, thereby improving readability while maintaining methodological consistency. |
| 6. Figures 3 & 4 (Recursive Plots): Ensure the y-axis scales are consistent across subplots to facilitate visual comparison of the magnitude of drops across indices. Clearly label the 2008 and 2020 periods with shaded vertical bands. | We thank the reviewer for this suggestion.  Figures 3 and 4 have been merged into a single figure with Panel A (LLRS) and Panel B (Hill) to facilitate direct comparison. Within each index, y-axis scales are standardized to ensure consistent visual interpretation of tail dynamics. In addition, periods corresponding to the 2007-2008 GFC and the 2020-2021 COVID-19 shock are now clearly indicated using shaded vertical bands. |
| **2.3 Literature Review and Context** | |
| 7. The literature review is comprehensive but could be better synthesized to directly motivate the paper's gap. Section2.1 adequately covers linkages (oil, tech) and risks (policy). Section 2.2 on indices is useful. However, the transition from the general literature to the specific gap on tail index estimation in renewable markets should be more forceful. The paragraph starting at line 101 does this, but it could be placed more prominently. | We thank the reviewer for this helpful suggestion.  In the revised manuscript, the transition from the general literature to the specific research gap has been strengthened and made more prominent. In particular, the discussion motivating tail index estimation and moment existence in renewable energy markets has been integrated more clearly into the Introduction (Section 1) and the early part of Section 2, ensuring that the gap addressed by the paper is highlighted earlier and more forcefully. This revision improves synthesis across Sections 2.1 to 2.2 and sharpens the motivation for the tail-based methodology adopted in the study. |
| 8. The review of heavy-tailedness methods (2.3) is good. Ensure all key methodological references (Gabaix &Ibragimov, 2011; Embrechts et al., 1997; etc.) are consistently cited. | We thank the reviewer for this comment.  All key methodological references related to heavy-tailedness and tail index estimation, most notably Embrechts et al. (1997) and Gabaix & Ibragimov (2011), are now consistently cited and clearly integrated throughout Section 2.3 and the methodological discussion. This ensures consistency between the theoretical foundations of heavy-tailed behavior and the empirical framework employed in the paper. |
| **2.4 Technical Details** | |
| 9. Data Transformation: The authors use simple returns rather than log returns due to "potential outliers" (p. 15). This is acceptable, but a brief justification in the context of tail estimation would be helpful. Do log returns dampen extreme values in a way that could bias tail index estimates? A sentence or two would suffice. | We thank the reviewer for this clarification request.  In the revised manuscript, all analyses are conducted using daily log returns, and the empirical results have been fully re-estimated accordingly. Log returns are standard in tail risk analysis and preserve relative extreme behavior while ensuring scale invariance and comparability across indices. This transformation avoids mechanical distortions without materially reducing tail events relevant for tail index estimation.  The main empirical conclusions remain unchanged under this specification. |
| 10. Stationarity (ADF Test): The results mentioned on p. 18 should be moved to a footnote or a brief paragraph in the main text. Simply stating "The return series are confirmed to be stationary via ADF tests (results available upon request)" is sufficient for the main flow. | We thank the reviewer for this suggestion.  To improve the flow of the main text, the stationarity results are now reported in Appendix Table A3, while the main text briefly notes that all return series are confirmed to be stationary based on ADF tests. This ensures completeness without interrupting the core analysis. |
| 11. Software and Code: For reproducibility, the authors should state the statistical software used (e.g., R, MATLAB, Python) and consider making their code available as part of the submission's supplementary materials, if the journal allows. | We thank the reviewer for this suggestion.  The manuscript has been revised to explicitly state the statistical software used. As noted in Section 3.4 (Data), all data transformations and empirical analyses were implemented using R.  In addition, a data availability statement has been added indicating that the data and code supporting the findings of this study are available from the authors upon reasonable request. |

**References**

Embrechts, P., Klüppelberg, C., & Mikosch, T. (1997). *Modelling Extremal Events*. Springer Berlin Heidelberg. https://doi.org/10.1007/978-3-642-33483-2

Gabaix, X. (1999). Zipf’s Law and the Growth of Cities. *American Economic Review*, *89*(2), 129–132. https://doi.org/10.1257/aer.89.2.129

Gabaix, X., Gopikrishnan, P., Plerou, V., & Stanley, H. E. (2003). A theory of power-law distributions in financial market fluctuations. *Nature*, *423*(6937), 267–270. https://doi.org/10.1038/nature01624

Gabaix, X., Gopikrishnan, P., Plerou, V., & Stanley, H. E. (2006). Institutional Investors and Stock Market Volatility. *The Quarterly Journal of Economics*, *121*(2), 461–504. https://doi.org/10.1162/qjec.2006.121.2.461

Gabaix, X., & Ibragimov, R. (2011). Rank — 1/2: A Simple Way to Improve the OLS Estimation of Tail Exponents. *Journal of Business & Economic Statistics*, *29*(1), 24–39.

Jondeau, E., & Rockinger, M. (2006). Optimal Portfolio Allocation under Higher Moments. *European Financial Management*, *12*(1), 29–55. https://doi.org/10.1111/j.1354-7798.2006.00309.x

**Reviewer #4 Comments and Author Response**

This manuscript addresses a relevant and timely topic: the tail risk characteristics of renewable energy stock indices in comparison to conventional energy indices. The authors employ a robust econometric framework—specifically, log-log rank-size (LLRS) regressions with optimal rank shifts and valid standard errors—to analyze the heavy-tailedness of return distributions from five major energy-sector indices over a 20-year period (2005-2025). The paper makes a valuable contribution to the literature by focusing on the often-overlooked extremal behavior and higher-moment properties of renewable energy returns, using a methodologically sound approach grounded in recent advances in tail index estimation.

The motivation is compelling. As global capital flows increasingly into ESG-compliant and sustainable assets, understanding the true downside risk profile of such investments is crucial for investors, risk managers, and policymakers. The authors correctly emphasize that the assumption of Gaussian or even finite fourth moments maybe misleading in real-world renewable energy markets—a point that merits greater attention in both academic and applied finance circles.

Nonetheless, despite its strengths, the paper suffers from several substantive methodological, interpretive, and structural shortcomings that collectively prevent it from being acceptable in its current form. I recommend major revision. Below, I provide a detailed, point-by-point critique organized by section. The comments aim not only to identify weaknesses but also to guide the authors toward a stronger, more rigorous, and publishable manuscript.

| **Comment** | **Response** |
| --- | --- |
| **Section 1 Introduction** | |
| The introduction effectively contextualizes the study within the climate-policy and decarbonization discourse. However, it suffers from redundancy and over-quotation of background facts (e.g., CO₂ emission statistics) that distract from the core econometric contribution. More importantly, the novelty claim is insufficiently articulated. | |
| - The abstract and introduction state that “heavy-tailedness and tail risk in renewable energy markets remain underexplored,” but this assertion is not rigorously defended. Recent works by Ji et al. (2018), Reboredo (2015), and Ghabri et al. (2021)—all cited in the paper—have examined extreme dependence and downside risk in clean energy markets using CoVaR, copulas, and wavelet methods. These studies do engage with tail behavior, albeit not via tail index estimation. The authors must more precisely delineate their contribution: not that tail risk is unexplored, but that the specific question of the existence and order of finite moments via tail index estimation has not been systematically addressed. This distinction is crucial. | We thank the reviewer for this important clarification.  We have revised the Abstract to more precisely delineate the study’s contribution. While acknowledging that prior research has examined downside risk, tail dependence, and extreme co-movements in renewable energy markets using approaches such as CoVaR, copulas, and wavelet methods, we now explicitly emphasize that our contribution lies in tail index estimation and CI-based inference on the existence and order of finite moments.  This distinction clarifies that the paper does not claim tail risk to be unexplored, but rather addresses a complementary and less systematically studied dimension of extreme risk relevant for model validity and risk measurement. |
| - The introduction also conflates volatility (second moment) with tail risk (extremal behavior beyond variance). For instance, the sentence “clean energy stocks’ volatility is typically greater than traditional energy stocks” (lines 83-84) is used to motivate a study of heavy tails—but higher volatility does not necessarily imply heavier tails (e.g., a scaled Gaussian has high volatility but no heavy tails). The authors must clarify that their interest lies not in conditional variance dynamics (GARCH-type) but in the unconditional extremal decay rate of the return distribution. | We thank the reviewer for this important clarification. We have revised Section 1 to clearly distinguish between volatility as a second-moment concept and tail behavior as the unconditional decay of extreme returns. In particular, we now explicitly state that higher volatility does not necessarily imply heavier tails and clarify that the focus of this study is on unconditional tail behavior rather than conditional variance dynamics.  These clarifications are incorporated in Section 1, paragraphs 4 and 5, beginning with “Renewable energy equity indices are often reported to exhibit higher volatility…”. |
| - Finally, the paper mentions the sample up to February 2025, which is in the future relative to the current date (2024). This raises concerns about data validity. Either this is a typographical error (should be 2024), or the authors are using simulated or forecasted data beyond the present, which must be explicitly disclosed and justified. | We appreciate the reviewer for raising this point.  We confirm that the sample period covers April 2005 to February 2025 and consists exclusively of realized historical daily observations obtained from the Bloomberg Terminal. No forecasted or simulated data are used.  To avoid any ambiguity, we have clarified the description of the data, indices, and sample period in Section 1, paragraph 8. |
| **Section 2 Literature Review** | |
| The literature review is extensive but somewhat disorganized. It falls into three subsections, but the connections between them are weak. | |
| **2.1 Renewable Energy Markets**   - This subsection surveys a broad range of papers on oil-renewables linkages, policy risk, and performance, but it lacks thematic focus. The authors should restructure this section to highlight only studies that are directly relevant to tail risk, extremal dependence, or distributional properties (e.g., skewness, kurtosis, tail dependence). Papers discussing mean-variance performance or general volatility spillovers are less relevant and dilute the narrative. Moreover, the claim that “renewable energy firms tend to over-perform in the long run” (lines 229-230) is presented as consensus, but this is contested in the literature (e.g., Rezec & Scholtens, 2017, cited as [53], actually argue that renewable investments are unattractive due to modest risk-adjusted returns). The authors should acknowledge this heterogeneity in empirical findings rather than presenting a monolithic view. | We thank the reviewer for this constructive comment regarding the focus and structure of Section 2.1.  We have revised this subsection to strengthen its thematic coherence and to emphasize studies directly related to tail risk, extremal dependence, and distributional properties of renewable energy returns. In particular, the revised text places greater emphasis on nonlinear dependence, tail behavior, crisis-related heavy-tailedness, and uncertainty-driven risk transmission, while de-emphasizing purely mean-variance interpretations.  In addition, we now explicitly acknowledge the heterogeneity of empirical findings regarding the performance of renewable energy investments. Rather than presenting long-run over-performance as a consensus view, the revised text highlights conflicting evidence in the literature and frames the “over-performance hypothesis” as contested.  These revisions are reflected in Section 2.1, paragraphs 3, 6, and 7; particularly in the discussion of tail-dependent relationships, crisis-related heavy-tailedness, and the revised treatment of performance-based studies. |
| **2.2 Renewable Energy Indices**  This subsection correctly describes the indices, but it misses an opportunity to critically evaluate their representativeness. For example:   - ECO and DJUSEN are U.S.-centric; SPG is global; ERIX is European. How does this geographic heterogeneity affect cross-index comparability? - SUN is sector-specific (solar), while others are broad clean energy. Does solar exhibit structurally different tail behavior due to technology cycles or policy shocks (e.g., tariff changes, silicon shortages)? These differences are later alluded to (e.g., SPG’s heavier tails attributed to emerging market exposure), but the conceptual groundwork should be laid here. | We thank the reviewer for this insightful comment.  We have revised Section 2.2 to move beyond a purely descriptive presentation of renewable energy indices and to explicitly address differences in geographic coverage and sectoral composition across indices. In particular, the revised text clarifies the U.S.-centric nature of ECO, the global scope of SPGCE, the European focus of ERIX, the sector-specific characteristics of the solar index, and the role of DJUSEN as a conventional energy benchmark.  These clarifications establish the conceptual groundwork for cross-index comparability and for potential differences in tail behavior observed in the empirical analysis. The relevant revisions are incorporated in Section 2.2, paragraphs 1, 2, 3, 5, and 6), as indicated by the highlighted text. |
| **2.3 Heavy-Tailedness and Inference**  This is the strongest part of the literature review. However, the authors overlook a critical debate in the extreme value theory (EVT) community: the choice of tail threshold. While they justify using 5% and 10% based on prior studies, they do not engage with the diagnostic tools available (e.g., mean excess plots, stability of Hill estimator across k) to validate this choice for their specific data. Moreover, the assumption of a pure power law (Pareto tail) is rarely satisfied in financial returns, which often exhibit sub exponential but non-Pareto tails (e.g., lognormal, stretched exponential).The robustness of LLRS to such deviations is claimed (citing Gabaix & Ibragimov, 2011), but this should be empirically verified (e.g., via goodness-of-fit tests or comparison with alternative tail models). | We thank the reviewer for this constructive and insightful comment.  In the revised manuscript, we expand Section 2.3 to explicitly acknowledge key methodological considerations in tail index estimation emphasized in the extreme value theory literature. In particular, we discuss the role of tail threshold selection and note that diagnostic tools such as mean excess plots and stability analysis of Hill estimates across varying tail sizes are commonly employed to assess appropriate tail regions. At the same time, we clarify that many empirical studies adopt fixed tail fractions (e.g., 5% and 10%) to ensure comparability across assets and to mitigate small-sample instability, an approach followed in the present study.  We have also included additional discussion and references providing theoretical and empirical support for heavy-tailed power-law frameworks in economics and finance. In particular, contributions by (Gabaix, 1999) and (Gabaix et al., 2003, 2006)  link the emergence of power-law tails in financial returns to economic mechanisms such as the Zipf distribution of firm and investor sizes and their trading impact. These results provide a structural rationale for heavy-tailed behavior in financial markets.  We explicitly acknowledge that financial return distributions may deviate from strict Pareto behavior and may exhibit sub-exponential or semi-heavy-tailed characteristics. In this context, we clarify that tail index estimation is not interpreted as testing a literal Pareto data-generating process. Rather, following Gabaix & Ibragimov (2011), the shifted LLRS estimator with corrected SE is employed to characterize effective tail thickness and the implied finiteness of moments under a broad class of heavy-tailed distributions.  As discussed in Section 3.3 and its references, LLRS inference with optimal rank shift and corrected SE exhibits robustness to deviations from exact power-law behavior, including slowly varying components and dependence structures typical of financial return series (e.g., volatility clustering and GARCH-type dynamics). We further note that classical goodness-of-fit procedures such as Kolmogorov-Smirnov tests rely on i.i.d. asymptotics and may exhibit limited power in finite samples, particularly when distinguishing between power-law and alternative heavy-tailed specifications.  Accordingly, our empirical objective is robust inference on tail thickness and moment implications, rather than validation of an exact Pareto specification. These clarifications are incorporated in Section 2.3 (paragraphs 6-8) of the revised manuscript. |
| **Section 3 Methodology and Data** | |
| **Methodological Concerns**  The paper’s core methodological innovation—LLRS with γ = −1/2—is well motivated. However, several issues arise: | |
| - Return Calculation: The authors state they use “daily simple returns as the percentage change… more appropriate than log returns due to the presence of potential outliers” (lines 409-411). This is incorrect. Simple returns can exceed−100%, leading to undefined log returns, but in practice, for stock indices, this is extremely rare. More importantly, log returns are additive over time and are standard in tail analysis because EVT asymptotic are typically derived for i.i.d. sequences, and log returns better approximate this. The use of simple returns may introduce bias in the tail, especially for large negative moves. The authors must justify this choice more rigorously or switch to log returns. | We thank the reviewer for this observation and agree.  The statement in the previous version suggesting the use of simple returns has been removed. We now compute daily log returns, and all estimations, tables, and figures have been re-generated accordingly. The revised return definition is documented in Section 3.4 paragraph 2.  The main empirical conclusions of the paper remain unchanged. |
| - Tail Index Estimation: The paper uses both LLRS and Hill estimators but relies primarily on LLRS. However, it does not address a key limitation: both methods assume i.i.d. data, yet financial returns exhibit volatility clustering and serial dependence. While Gabaix & Ibragimov (2011) argue LLRS is robust to GARCH-type dependence, this is only asymptotically true under specific conditions. The authors should: - Test for serial dependence in absolute/ squared returns (e.g., Ljung-Box). - Consider blocking methods or subsampling to account for dependence (e.g., Hall et al., 1998). - At minimum, report results using filtered returns (e.g., standardized residuals from a GARCH(1,1) model) to isolate the tail behavior of innovations. | We thank the reviewer for raising the important issue of serial dependence and volatility clustering in financial return series.  In the revised manuscript, we explicitly acknowledge the limitations of i.i.d. assumptions for both Hill and LLRS estimators and discuss their implications for tail inference in financial applications (Section 3.1).  We further clarify the robustness properties of the shifted LLRS estimator in Section 3.3 paragraph 3, emphasizing its favorable robustness properties under certain forms of nonlinear dependence and volatility clustering documented in the literature.  To complement these theoretical considerations, we implement formal diagnostic and robustness checks. Specifically, we test for serial dependence using Ljung-Box diagnostics, apply GARCH(1,1) filtering to obtain standardized return innovations, and re-estimate tail indices using both raw and filtered returns (Section 3.3, paragraph 4; see Table A1, Figure A1, and Table A2 in the Appendix).  These additions ensure that the dependence issue is explicitly acknowledged, empirically assessed, and shown not to alter the main conclusions of the paper. |
| - Confidence Intervals: The CIs for LLRS assume asymptotic normality of the OLS estimator. However, with n as small as 250 (5% of 5000), and given potential deviations from power laws, these CIs may be unreliable. The authors should consider bootstrap CIs (e.g., percentile-t bootstrap) to assess finite-sample accuracy. | We thank the reviewer for this thoughtful comment regarding finite-sample accuracy of confidence intervals.  In this study, tail inference is based on the LLRS regression with optimal rank shift $(\gamma= 1/2)$ and corrected SE as proposed by (Gabaix & Ibragimov, 2011). This estimator is specifically designed to eliminate leading-order finite-sample bias and to provide reliable asymptotic inference under deviations from exact power-law behavior and under dependence structures commonly observed in financial returns.  While we acknowledge that resampling methods such as bootstrap confidence intervals can be informative in some settings, bootstrap procedures for heavy-tailed and dependent data are themselves non-trivial and require additional assumptions regarding dependence structure and block length selection. For this reason, and consistent with much of the empirical EVT literature, we rely on analytically derived confidence intervals and emphasize CI-based interpretation rather than precise point estimates.  Importantly, our conclusions are qualitative and comparative in nature and are robust across alternative tail fractions and volatility-filtered return series. The main empirical findings are therefore not sensitive to small-sample variation in confidence interval construction. |
| - Recursive Estimation: The recursive window approach (Section 4.2, Figs 3-4) is useful for detecting structural breaks. However, the window starts at 500 observations—arbitrary without justification. Why not test sensitivity to initial window size (e.g., 300 vs. 700)? Moreover, recursive estimates are highly autocorrelated, so the apparent “stability” post-2008 may be an artifact of persistence, not true stationarity. | We thank the reviewer for this insightful comment regarding the recursive estimation scheme.  In the revised manuscript, the recursive window is initialized with 500 observations as a pragmatic compromise between ensuring a sufficient number of tail observations for reliable estimation and retaining sensitivity to major structural changes in tail behavior. This choice is not presented as optimal but reflects a balance between estimation precision and responsiveness that is commonly adopted in recursive tail-risk analysis.  We further clarify in Section 4.2, and in the notes to Figures 3 and 4, that recursive tail index estimates obtained from expanding windows are inherently autocorrelated and should be interpreted as descriptive diagnostics of evolving tail behavior rather than as formal tests of stationarity. Accordingly, the apparent post-crisis smoothness in the recursive estimates is not interpreted as evidence of strict stationarity, but reflects both gradual changes in tail risk and the mechanical persistence induced by the recursive estimation procedure.  These clarifications ensure that the recursive analysis is used appropriately to visualize regime shifts and structural changes, while avoiding over-interpretation of persistence or stability in the estimated tail indices. |
| **Data Concerns** | |
| - Sample Period: The paper claims data up to February 2025. Unless this is a typo, this is problematic. If real data exist beyond mid-2024, the authors must clarify the source and vouch for its reliability. If it includes forecasts or simulated data, this must be disclosed, as it invalidates tail inference. | We thank the reviewer for this comment.  As this concern is closely related to the earlier comment raised in Section 1, we have ensured consistency and clarity throughout the manuscript.  In addition to the clarification provided in Section 1, we have explicitly adjusted Section 3.4 paragraph 3 and 4 to confirm that the sample period from April 2005 through February 2025 consists exclusively of realized historical daily observations obtained from the Bloomberg Terminal, with no use of forecasted or simulated data. |
| - Index Definitions: Table 1 states SPG “aims to include 100 constituents,” but the current S&P Global Clean Energy Index actually has ~30 constituents. This discrepancy suggests the authors may be using an outdated or modified version. This must be clarified. | We thank the reviewer for identifying this issue.  In the previous version, the description of the SPGCE index inadvertently referred to the “S&P Global Clean Energy Transition Index” which differs from the index actually used in the analysis.  We have corrected Table 1 (Section 3.4) to reflect the “S&P Global Clean Energy Select Index”, which measures the performance of approximately 30 large clean energy companies from developed and emerging markets.  Note: The revised definition is now fully consistent with the Bloomberg data series employed in the empirical analysis. |
| - Stationarity: The ADF results (mentioned in passing) confirm return stationarity, which is standard. But for tail inference, strict stationarity is required. The recursive plots show clear non-stationarity in tail behavior (e.g., 2008crash). The authors should discuss whether their pooled tail estimates mask important time-varying dynamics. | We thank the reviewer for this important comment.  In the revised manuscript, we explicitly report ADF unit root test results for both price levels and daily log returns in Table A3 (Appendix). These results confirm that all return series are stationary across specifications, which is a standard prerequisite for tail analysis.  At the same time, we do not assume strict stationarity of tail behavior. Time variation in tail risk is explicitly documented through the recursive tail index estimates presented in Section 4.2 (Figures 3 and 4), which reveal pronounced shifts during major market stress episodes such as the 2008 financial crisis. Accordingly, pooled tail index estimates are interpreted as long-run characterizations of tail behavior over the full sample period, rather than as evidence of time-invariant tails. |
| **Section 4 Descriptive and correlation analysis** | |
| **4.1 Return Plots and Summary Statistics**  Figure 1 is helpful but lacks quantitative annotations (e.g., vertical lines for crisis dates). Table 2 shows high kurtosis for all indices, consistent with heavy tails. However, kurtosis is a poor indicator of tail heaviness in small samples or under infinite fourth moments—precisely the issue the paper studies. The authors should de-emphasize kurtosis and focus on tail index estimates. | We thank the reviewer for this constructive comment.  Figure 1 has been revised to include explicit annotations for major global stress events, with shaded regions highlighting the GFC and the COVID-19. In addition, the figure has been extended to jointly present price levels and returns to provide broader market context.  We have also revised Section 4.1 paragraph 4 and Table 2 to de-emphasize kurtosis as a measure of tail heaviness. As clarified in the main text and table notes, kurtosis and normality tests are reported for descriptive purposes only, while formal inference on tail behavior and moment existence is based on tail index estimates and CI-based inference presented in Section 4.2. |
| **4.2 Tail Risk and Tail Index Estimates**  This is the core of the paper, but interpretation is inconsistent.   - Point Estimates vs. CIs: The authors repeatedly compare point estimates (e.g., “SPG has the smallest tail index”) but then note that CIs overlap, implying no statistical difference. This is contradictory. If differences are not significant, they should not be emphasized. The conclusion that “renewable energy indices are less heavy-tailed than conventional” (Abstract) is not supported by statistical tests—only by point estimates. A formal test (e.g., bootstrap difference in tail indices) is needed. | We thank the reviewer for this important clarification.  In the revised manuscript, we have removed all interpretive emphasis on point-estimate comparisons and now base inference exclusively on CI-based reasoning. Section 4.2 has been substantially rewritten to ensure internal consistency between estimation results and their interpretation.  Specifically, we now explicitly state that overlapping CIs imply no statistically distinguishable differences in tail heaviness across renewable and conventional energy indices, and any numerical variation in point estimates is treated as directional only rather than evidence of statistical dominance (see Section 4.2 paragraphs 1, 5, and 9). As a result, the manuscript no longer draws conclusions about renewable energy indices being “less heavy-tailed” based on point estimates alone.  In line with this revision, the corresponding claims in the Abstract, Section 1 Introduction and Section 5 Discussion have been adjusted to reflect CI-based inference.  Given that CI overlap already provides a formal statistical criterion for non-distinguishability, we do not introduce additional bootstrap-based difference tests, which would not alter the substantive conclusions. |
| - Moment Existence: The inference that “all renewable indices have finite second moments” relies on ζ̂> 2. But at 5% truncation, the lower bound of the CI for SPG (LLRS) is 2.34—comfortably above 2. However, at 10%, it’s 2.32, still fine. But what if the true ζ is 1.99? The authors assume the point estimate is the truth, but hypothesis testing should be based on CIs. For example, to claim “finite variance,” one must show the entire CI lies above 2. For SPG10%(LLRS), 2.32 > 2, so OK—but for emerging market indices not included, this may fail. The authors are careful here, but the language could be tightened. | We thank the reviewer for this careful clarification.  In the revised manuscript, we have tightened the language on moment existence to rely strictly on CI-based inference, rather than on point estimates.  In Section 4.2 paragraphs 6-7, we now explicitly state that conclusions regarding finite second moments are drawn **only when the entire CI lies above the threshold** $\boldsymbol{\zeta=2}$. For all indices considered in the sample, including SPGCE at both truncation levels, the **lower bounds (LBs) of the reported CIs exceed**$\boldsymbol{\zeta=2}$, supporting finite variances under CI-based hypothesis testing. We no longer rely on point estimates as indicative of the true tail index.  At the same time, we acknowledge that inference regarding higher-order moments is less definitive. Where CIs extend across $\zeta=3$ or $\zeta=4$, we refrain from asserting finiteness and instead characterize third and fourth moments as **potentially infinite**, consistent with the reviewer’s recommendation. The revised text avoids extrapolation beyond the observed sample and does not generalize conclusions to indices outside the empirical scope of the study.  These revisions ensure that statements about moment existence are fully aligned with formal CI-based hypothesis testing and remove any implicit reliance on point estimates. |
| - Asymmetry (Tables 4-5): The gain/loss asymmetry analysis is valuable. However, the authors note that CIs for left/right tails overlap, yet still claim “downward moves… are somewhat more pronounced.” This is misleading. Without a significant difference, such statements should be qualified as directional tendencies, not established facts. | We thank the reviewer for this important observation. In the revised manuscript, we have revised the asymmetry discussion to ensure that interpretation is fully aligned with CI-based inference.  Specifically, in Section 4.2 paragraphs 8-9, we now state explicitly that while point estimates of right- and left-tail indices may differ, the confidence intervals overlap across indices, truncation levels, and estimation approaches. Accordingly, such differences are interpreted only as directional tendencies, rather than as statistically established gain/loss asymmetries.  All language suggesting that downside moves are “more pronounced” has been removed or carefully qualified, and the revised text emphasizes that CI-based inference does not support strong conclusions regarding systematic dominance of downside or upside tail risk. This ensures consistency between the empirical results reported in Tables 4-5 and their interpretation. |
| - Recursive Results (Figs 3-4): The drop in tail index during 2008 is dramatic, especially for SPG (below 2). This implies infinite variance during crises—a critical finding that deserves more discussion. Yet the authors treat it as a transient shock. They should explore: - The duration of sub-2 tail indices. - Whether such episodes invalidate standard risk models during crises, even if normality resumes later. - Comparison with other crises (e.g., 2020, Ukraine war)—why was 2020 milder? | We thank the reviewer for this important observation.  We have revised Section 4.2 and consolidated the former Figures 3 and 4 into a single revised Figure 3 with Panel A (LLRS) and Panel B (Hill) to facilitate direct comparison of recursive tail dynamics. The revised discussion now explicitly addresses:  (i) the duration of episodes in which recursive tail indices fall below $\zeta=2$ during the 2007-2008 GFC, indicating periods of potentially infinite variance;  (ii) the implications of such episodes for the validity of standard finite-variance risk models during crises, even when tail behavior stabilizes thereafter; and  (iii) comparisons with later stress events, showing that the COVID-19 episode in 2020 was associated with materially smaller and more short-lived declines in tail indices.  These crisis episodes are explicitly interpreted as temporary breakdowns of finite-variance assumptions rather than as permanent structural changes in unconditional tail behavior. The revised discussion clarifies that crisis-period tail dynamics are state-dependent and that the GFC represents a more severe tail-risk regime than subsequent shocks, without implying long-run structural instability. |
| **Section 5 Discussion** | |
| - Risk Management: The authors argue VaR/ES are “appropriate” because ζ > 1. But VaR is incoherent for ζ ≤ 1, while ES is coherent only if ζ > 1 (Embrechts et al., 1997). Since all ζ̂> 2, ES is fine—but the authors should cite the precise conditions from risk measure theory. | We thank the reviewer for this important clarification.  Section 5 has been revised to explicitly state the theoretical conditions under which VaR and ES are well defined. In particular, Section 5 paragraph 5 now clarifies that VaR is non-coherent for distributions with $\zeta\leq1$, while ES is coherent only when first moments are finite ($\zeta>1$), consistent with established risk-measure theory (see [15,91,98], including Embrechts et al. (1997)). Since CI-based inference in Section 4.2 paragraphs 6-7 supports $\zeta>2$ for all indices considered, the revised discussion emphasizes that ES is theoretically appropriate in the present setting, while also cautioning against underestimating tail risk during periods of extreme market stress. |
| - Portfolio Theory: The discussion of Markowitz and MVS portfolios is relevant but superficial. If ζ ≈ 3, then skewness may be unstable (infinite third moment), making MVS optimization numerically unreliable. The authors should cite empirical studies showing MVS failure under heavy tails (e.g., Jondeau & Rockinger, 2006). | We thank the reviewer for this insightful comment.  Section 5 has been revised to deepen and sharpen the discussion of portfolio theory under heavy-tailed returns. In Section 5 paragraphs 6 and 7, we now explicitly acknowledge that while CI-based inference supports the use of mean-variance frameworks ($\zeta>2$), inference on third moments is less conclusive when $\zeta$ is close to 3. This implies that skewness may be unstable or effectively infinite, rendering mean-variance-skewness (MVS) portfolio optimization numerically unreliable in heavy-tailed environments.  To substantiate this point, we now explicitly cite empirical evidence from Jondeau and Rockinger (2006), which documents the poor performance and instability of skewness-based portfolio strategies under heavy-tailed return distributions.  The revised text therefore clarifies the conditions under which Markowitz-type approaches remain applicable, while cautioning against uncritical application of MVS frameworks in renewable energy markets characterized by pronounced tail risk. |
| - Policy Implications: The claim that renewables are “lower-risk” could encourage overallocation to green assets. But if tail risk is underestimated (e.g., due to climate policy shocks), this could backfire. The authors should discuss this moral hazard. | We thank the reviewer for this important observation.  We have revised Section 5 paragraphs 9 and 10 to explicitly avoid characterizing renewable energy assets as inherently “lower-risk” and to caution against over-allocation based on point estimates.  The revised discussion emphasizes that CI-based inference does not support statistically significant differences in tail heaviness between renewable and conventional energy markets, and highlights the potential moral hazard arising from underestimation of tail risk in the presence of climate policy uncertainty, regulatory shifts, or geopolitical shocks.  The policy implications are therefore reframed toward prudent diversification, stress testing, and dynamic tail-risk monitoring. |
| **Section 6 Conclusion** | |
| The conclusion reiterates findings but misses an opportunity to outline a research agenda. Future work should include:   - Multivariate tail dependence (e.g., via copulas). - Real-time tail monitoring systems for renewable portfolios. - Integration with climate stress testing (e.g., NGFS scenarios). | We thank the reviewer for this valuable suggestion.  We have substantially revised Section 6 Conclusion to strengthen its forward-looking component and to explicitly outline a clear research agenda. In particular, we now:  (i) propose extensions to multivariate tail dependence analysis using copula-based frameworks;  (ii) discuss the development of real-time tail monitoring systems based on recursive tail estimation; and  (iii) explicitly highlight the integration of tail-risk analysis with climate stress-testing frameworks, including NGFS scenario-based assessments.  These additions are presented in the final paragraphs of Section 6 and directly address the reviewer’s recommendations. |

| **Other Issues** | |
| --- | --- |
| - Writing and Clarity: The manuscript suffers from grammatical errors, inconsistent notation (e.g., ζ vs. α for tail index),and formatting issues (e.g., line breaks in equations). Professional copyediting is advised. | We thank the reviewer for this comment.  The manuscript has been thoroughly revised and language-edited throughout, with particular attention to grammatical consistency, notation (using a single tail-index symbol consistently), and formatting of equations and text.  We believe these revisions have substantially improved clarity and presentation. |
| - Figures: Figures 1-4 are referenced but not included in the PDF. Without them, assessing the recursive results is difficult. Ensure figures are embedded or provided separately. | We thank the reviewer for pointing this out.  All figures and tables are now included in the revised manuscript to facilitate readability and assessment during review.  We will, of course, comply with PLOS ONE’s figure-submission requirements (e.g., in a separate document) at the production stage if the manuscript is accepted. |
| - Reproducibility: No code or data availability statement is provided. PLOS ONE requires this. The authors should commit to sharing replication files. | We agree with the reviewer on the importance of reproducibility.  A Data Availability statement has now been added (before References), confirming that the data and replication codes used in this study will be made available upon reasonable request, in line with PLOS ONE’s data-sharing policy. |
| - References: Some references are incomplete (e.g., [39] lacks journal name in the main text). Ensure all follow a consistent style. | We thank the reviewer for noting this.  All references have been carefully checked, completed, and harmonized to ensure consistency with journal style and full bibliographic information using a refence management system. |

**References**

Embrechts, P., Klüppelberg, C., & Mikosch, T. (1997). *Modelling Extremal Events*. Springer Berlin Heidelberg. https://doi.org/10.1007/978-3-642-33483-2

Gabaix, X. (1999). Zipf’s Law and the Growth of Cities. *American Economic Review*, *89*(2), 129–132. https://doi.org/10.1257/aer.89.2.129

Gabaix, X., Gopikrishnan, P., Plerou, V., & Stanley, H. E. (2003). A theory of power-law distributions in financial market fluctuations. *Nature*, *423*(6937), 267–270. https://doi.org/10.1038/nature01624

Gabaix, X., Gopikrishnan, P., Plerou, V., & Stanley, H. E. (2006). Institutional Investors and Stock Market Volatility. *The Quarterly Journal of Economics*, *121*(2), 461–504. https://doi.org/10.1162/qjec.2006.121.2.461

Gabaix, X., & Ibragimov, R. (2011). Rank — 1/2: A Simple Way to Improve the OLS Estimation of Tail Exponents. *Journal of Business & Economic Statistics*, *29*(1), 24–39.

Jondeau, E., & Rockinger, M. (2006). Optimal Portfolio Allocation under Higher Moments. *European Financial Management*, *12*(1), 29–55. https://doi.org/10.1111/j.1354-7798.2006.00309.x
