# Supplementary material for: Tail risk, large fluctuations and downfalls in renewable energy markets
Source: PLoS One. 2026 Jul 15;21(7):e0351106. doi: 10.1371/journal.pone.0351106 (PMC13372164; doi:10.1371/journal.pone.0351106)
Supplement: Attachment — Submitted filename: PONE-D-24-59227_Response to Editor and Reviewers (Rev3).docx [file pone.0351106.s011.docx]

**Academic Editor Comments and Author Response**

**Academic Editor (AE) – Overall Comment:**

As the Academic Editor, I have carefully evaluated your revised manuscript along with the feedback from the three reviewers. While Reviewer #1 is satisfied with your previous revisions, Reviewers #3 and #4 have raised substantial concerns that must be addressed. Both reviewers agree that the empirical analysis is robust and timely; however, they require deeper economic contextualization, stronger methodological justification, and improvements to the manuscript's structure. Therefore, I am recommending a Major Revision. Please ensure that your next submission comprehensively addresses the following key areas

| **AE.1 Economic Context and Policy Implications** | |
| --- | --- |
| **AE.1.1 Real-Economy Linkages** | **Response** |
| **AE-C1:** Broaden your motivation to explicitly connect financial tail risk with the socio-economic goals of the global energy transition. Discuss how high tail risk impacts the cost of capital, infrastructure deployment, and climate targets. | We sincerely thank the Academic Editor for this insightful and constructive comment.  In response, we have revised Section 1 and Section 5 to more explicitly connect financial tail risk with the broader socio-economic dimensions of the energy transition, including financing conditions, infrastructure investment, capital allocation, and the challenges associated with the transition toward low-carbon energy systems.  Specifically, we have:   - strengthened the discussion linking tail risk with renewable energy financing conditions and required risk premia; - clarified how elevated tail risk may affect long-term infrastructure investment and clean-energy deployment; and - emphasized the importance of reliable tail-risk measurement for maintaining stable investment environments in support of global decarbonization efforts.   These revisions, highlighted in blue, have been incorporated primarily in Section 1 Introduction and Section 5 Discussion. |
| **AE.1.2 Systemic and Regulatory Implications** | **Response** |
| **AE-C2:** Elaborate on the implications of your findings for policymakers and regulators. Specifically, address how the potential for infinite higher moments affects standard risk management tools (e.g., mean-variance optimization, VaR, ES estimation risks) and regulatory stress testing. | We thank the reviewer for this important observation.  In response, we have expanded Section 5 to further elaborate on the implications of heavy-tailedness and potentially non-finite higher moments for financial risk management, portfolio construction, and regulatory oversight in renewable energy markets.  Specifically, we have:   - clarified the conditions under which commonly used risk measures such as VaR and ES remain theoretically valid under heavy-tailed return distributions; - expanded the discussion of the limitations and potential instability of higher-moment-based portfolio optimization approaches, particularly in the presence of potentially non-finite third and fourth moments; - strengthened the interpretation regarding the implications of heavy-tailedness for econometric modelling and volatility-based risk forecasting frameworks; and - incorporated an explicit discussion of regulatory stress-testing considerations, emphasizing that underestimation of tail heaviness may result in insufficient capital buffers and inadequate assessment of systemic downside risk during severe market stress.   These revisions, highlighted in blue, have been incorporated primarily in Section 5 Discussion, with additional supporting clarification in Sections 4 and 6. |
| **AE.1.3 Economic Drivers** | **Response** |
| **AE-C3:** Provide a clearer explanation of the underlying economic drivers that cause renewable and conventional energy sectors to exhibit similar tail behaviors and responses to systemic stress. | We thank the reviewer for this valuable observation.  In response, we have expanded Section 5 to provide a clearer economic interpretation of why renewable and conventional energy assets may exhibit broadly similar tail behavior during periods of systemic stress.  Specifically, we have:   - clarified that renewable and conventional energy equities are increasingly integrated into broader global financial markets and are therefore exposed to common macro-financial shocks; - emphasized the role of liquidity contractions, shifts in investor risk sentiment, and systemic deleveraging during crisis periods as potential drivers of comparable tail-risk dynamics across energy sectors; and - further explained that similarities in tail behavior may arise despite important differences in underlying technologies, market structures, and business models.   These revisions highlighted in blue have been incorporated primarily in Section 5 Discussion, with additional supporting context in Sections 1 and 4. |
| **AE.1.4 Crisis Comparison** | **Response** |
| **AE-C4:** Deepen your analysis regarding the COVID-19 pandemic versus the 2007-2008 Global Financial Crisis. Explain why the tail risk response was notably more modest during the pandemic. | We thank the reviewer for this thoughtful observation.  In response, we have expanded the discussion of the recursive tail analysis to provide a clearer comparison between the 2007-2008 GFC and the COVID-19 pandemic period.  Specifically, we have:   - clarified that the recursive tail index estimates indicate a substantially stronger increase in tail heaviness during the GFC relative to the COVID-19 shock; - discussed the differing nature of the two crises, emphasizing that the GFC originated within the financial system and was associated with severe banking distress, prolonged deleveraging, and elevated systemic uncertainty; - explained that the COVID-19 shock was comparatively more exogenous in nature and accompanied by rapid monetary and fiscal policy interventions, which may have mitigated the persistence and severity of tail-risk amplification; and - further highlighted that these differences are consistent with the comparatively milder recursive tail dynamics observed during the pandemic period across the energy indices examined.   These revisions, highlighted in blue, have been incorporated primarily in Section 4 Results, with additional synthesis in Sections 5 and 6. |

| **AE.2 Methodological Rigor and Robustness** | |
| --- | --- |
| **AE.2.1 Higher-Order Moments** | **Response** |
| **AE-C5:** Provide more rigorous sensitivity analyses or additional statistical tests to solidify your claims regarding the potential infiniteness of third and fourth moments, as these claims currently appear speculative. | We thank the reviewer for this important observation.  In response, we have revised the manuscript to ensure that all statements regarding higher-order moments are grounded strictly in CI-based inference and are not presented as definitive claims of literal infiniteness in finite samples.  Specifically, we have:   - clarified that moment-existence conclusions are based on the position of the reported CIs relative to the theoretical thresholds $\zeta=1$, $\zeta=2$, $\zeta=3$, and $\zeta=4$; - emphasized that finite first and second moments are supported only when the lower bounds (LB) of the reported CIs exceed the relevant threshold; - revised the interpretation of third and fourth moments to state that inference is inconclusive where CIs cross $\zeta=3$ or extend below $\zeta=4$; and - clarified that these results should be interpreted as evidence of extremely large and unstable higher-moment behavior rather than as definitive proof of literal divergence in finite samples.   These revisions, highlighted in blue, have been incorporated primarily in Sections 2.3, 4.2, 5, and 6. |
| **AE.2.2 Truncation Levels** | **Response** |
| **AE-C6:** Justify the choice of 5% and 10% truncation levels. Explicitly discuss why confidence intervals widen significantly at the 5% level and what this suggests about power-law behavior in the extreme tail. | We thank the reviewer for this constructive observation regarding the choice of truncation levels.  In response, we have expanded the discussion of the 5% and 10% truncation thresholds and further clarified the interpretation of the associated CIs in Section 4.2.   - The revised manuscript now explains that these truncation levels are widely used in the EVT and financial tail-risk literature and are intended to balance asymptotic tail inference with sufficient finite-sample precision. - We further clarify that CIs widen at lower truncation levels because fewer extreme observations are retained in the estimation sample, thereby increasing estimation uncertainty in the deepest tail region. At the same time, the substantial overlap of CIs across truncation levels supports the overall robustness of the inferred power-law behavior and indicates that the main conclusions are not driven by a specific threshold selection. - We additionally refer to the truncation-stability analysis presented in Figure 2, which evaluates LLRS-based tail index estimates across a broader range of truncation levels from 2.5% to 15%.   These revisions, highlighted in blue, have been incorporated primarily in Section 4.2. |

| **AE.2.3 GARCH Filtered Residuals** | **Response** |
| --- | --- |
| **AE-C7:** Move the discussion of the GARCH(1,1) filtered residuals from the appendix to the main text. Address the discrepancy between tail index estimates for raw returns versus standardized residuals, clarifying whether the observed heavy-tailedness is driven by conditional heteroskedasticity or unconditional distributional properties. | We thank the reviewer for this valuable suggestion.  In response, we have moved the discussion of the GARCH(1,1)-filtered residual analysis from the Appendix into the main text and expanded the interpretation of the corresponding robustness results.   - We clarify that GARCH filtering reduces the influence of volatility clustering and conditional heteroskedasticity, leading in several cases to somewhat higher tail index estimates, implying comparatively thinner tails. - At the same time, the persistence of heavy-tailed behavior in the standardized residuals indicates that extremal risk cannot be attributed solely to time-varying volatility dynamics and also reflects broader unconditional distributional properties.   These revisions, highlighted in blue, have been incorporated primarily in Section 3.3, with supporting reference to Appendix Figure A1 and Table A2. |
| **AE.2.4 Recursive vs. Rolling Windows** | **Response** |
| **AE-C8:** Discuss the sensitivity of your recursive expanding-window approach to the initial 500-observation window size. Include a comparative discussion on how a rolling window might differentiate between persistent structural breaks and temporary shock absorption. | We thank the reviewer for this helpful suggestion regarding the recursive tail analysis.   - The revised manuscript now explains that the recursive procedure begins with an initial estimation window of 500 daily observations and subsequently expands the sample one observation at a time until the end of the sample period. - We further clarify that recursive estimation permanently retains past extreme events in subsequent estimation windows and is therefore particularly suitable for assessing the persistence and long-run influence of systemic shocks on tail behavior. - In contrast, rolling-window approaches may produce more transitory fluctuations in estimated tail risk because earlier crisis observations eventually exit the estimation window, thereby allowing stronger mean reversion in tail estimates over time.   These revisions, highlighted in blue, have been incorporated primarily in Section 4.2, with additional discussion in Section 6. |

| **AE.3 Structure, Presentation, and Clarity** | |
| --- | --- |
| **AE.3.1 Streamline Introduction** | **Response** |
| **AE-C9:** Condense the excessively long introduction and literature review. Specifically, integrate the background descriptions of the renewable energy indices more concisely into the Data or Methodology sections. | We thank the reviewer for this constructive observation regarding the structure and presentation of the manuscript.  In response, we have streamlined and refined both Sections 1 and 2 to improve readability, reduce repetition, and strengthen the overall flow of the paper.   - In particular, we condensed several background discussions and integrated parts of the descriptive material on renewable energy indices more directly into Sections 2 and 3, where these details are more naturally connected to the data and empirical framework. - At the same time, we retained Sections 1 and 2 as separate sections to preserve conceptual clarity and maintain a clear distinction between the study motivation, prior literature, and methodological setup.   These revisions, highlighted in blue, have been incorporated primarily in Sections 1-3. |
| **AE.3.2 Truncation Levels** | **Response** |
| **AE-C10:** Explicitly clarify the end date of your dataset (stated as February 2025). Ensure this is accurate, provide exact cut-off dates, and include Bloomberg terminal codes for full replicability. Briefly comment on how index weighting schemes (equal-dollar vs. market-cap) might influence tail properties. | We thank the reviewer for this helpful observation regarding data transparency and replicability.  In response, we have revised Section 3.4 to explicitly clarify the sample period and include the Bloomberg tickers corresponding to all indices analyzed in the study. Specifically, the revised manuscript now states that the price series cover the period from 1 April 2005 to 25 February 2025, with return calculations beginning on 4 April 2005 due to the log-return transformation.  We have also added the Bloomberg tickers directly to Table 1 and briefly clarified that differences in weighting schemes and index construction may contribute to differences in tail behavior across indices.  These revisions, highlighted in blue, have been incorporated primarily in Section 3.4 and Table 1. |
| **AE.3.3 GARCH Filtered Residuals** | **Response** |
| **AE-C11:** Improve the readability of Tables 3, 4, and 5 by clearly separating confidence intervals from point estimates. Ensure Figure 1 Panel B is legible in grayscale, and state the standardized scale in Figure 3's caption. Carefully proofread all equations and ensure consistent notation throughout the manuscript. | We thank the reviewer for this helpful observation regarding presentation clarity and consistency.  In response, we carefully reviewed the formatting, notation, and presentation of the empirical results throughout the manuscript.  Specifically, we refined the presentation of Tables 3 to 5 to improve the visual separation between point estimates and CIs, clarified in the caption of Figure 3 that the y-axes are standardized within each index for comparability purposes, and reviewed the figures to ensure readability under grayscale reproduction.  We additionally conducted a careful proofreading of equations, symbols, and notation to improve consistency throughout the manuscript.  These revisions, highlighted in blue, have been incorporated throughout the revised manuscript. |

**Reviewer #3 Comments and Author Response**

We sincerely thank **Reviewer 3** for the detailed, thoughtful, and constructive comments provided on the manuscript. We greatly appreciate the reviewer’s recognition of the methodological contribution of the paper, as well as the numerous suggestions aimed at strengthening the broader contextualization, policy relevance, and practical implications of the study.

Many of the methodological, interpretational, and presentation-related observations raised by **Reviewer 3** substantially overlapped and were subsequently synthesized by the Academic Editor (AE) into a consolidated set of editorial comments (AE-C1 to AE-C11). In addition, several comments addressed closely related themes across different parts of the manuscript. For clarity and to avoid unnecessary repetition, we therefore grouped the reviewer’s observations into **seven (7) broader thematic comment categories**, presented in the table below, and refer where appropriate to the corresponding AE responses in which the associated revisions are discussed in greater detail.

Overall, the revised manuscript strengthens the discussion of the economic and policy relevance of tail risk in renewable energy markets, clarifies the interpretation of CI-based inference regarding moment existence, expands the methodological discussion surrounding truncation levels and recursive estimation, integrates the discussion of GARCH-filtered residuals into the main text, and improves the overall clarity, consistency, and presentation of the empirical results.

**Reviewer 3 – Overall Comment:**

The manuscript presents a timely and empirically rigorous analysis of tail risk and heavy-tailed behavior in renewable energy equity markets compared to conventional energy benchmarks. The authors employ robust econometric techniques, specifically the Log-Log Rank-Size (LLRS) regression with optimal rank shift and Hill's estimator, to assess the existence of finite moments across five major energy indices. The use of a recursive expanding-window approach to capture time-varying tail risk during systemic stress events, such as the Global Financial Crisis and the COVID-19 pandemic, adds significant value to the existing literature. The finding that renewable energy indices exhibit heavy tails similar to conventional energy assets challenges the notion that green assets are inherently safer from an extreme risk perspective. However, while the statistical analysis is sound, the manuscript requires substantial revisions to better contextualize these financial findings within the broader socio-economic and policy frameworks governing the energy transition. The discussion on implications for investors and policymakers needs deepening, and the motivation section requires strengthening to align with broader sustainability goals. Consequently, I recommend a Major Revision to address the following concerns.

| **Comments** | **Response** |
| --- | --- |
| **R3-C1. Motivation, socio-economic context, and policy relevance**:  Reviewer 3 requested a stronger connection between financial tail risk and the broader socio-economic objectives of the global energy transition, including renewable energy financing, infrastructure deployment, capital allocation, and regulatory stress testing. The reviewer further encouraged a clearer discussion of how extreme downside risk may affect the cost of capital, long-term energy investment, and the stability of renewable energy markets. | We thank **Reviewer 3** for this important observation regarding the broader economic and policy relevance of tail risk in renewable energy markets.  Sections 1, 5, and 6 were revised to strengthen the discussion linking tail risk to renewable energy financing, capital allocation, infrastructure investment, and regulatory stress testing. We also expanded the discussion of the implications of higher-order moment uncertainty for portfolio construction, VaR/ES-based risk management, and systemic financial stability in energy markets.  These revisions overlap substantially with the Academic Editor’s synthesized comments AE-C1 to AE-C4 discussed above. |
| **R3-C2. Broader sustainability, resource-governance, and energy-transition literature**:  Reviewer 3 suggested positioning the study more explicitly within the broader sustainability, circular economy, resource-governance, and energy-transition literature, including the incorporation of several additional references related to socio-economic development, sustainable practices, and energy/environmental systems. | We thank **Reviewer 3** for the helpful suggestions regarding the broader sustainability and energy-transition literature.  We reviewed the suggested references and further strengthened the broader policy and sustainable-investment framing of the manuscript, particularly in Sections 1, 5, and 6. At the same time, the paper remains primarily positioned within the EVT and financial tail-risk literature, and the revisions were therefore implemented in a manner consistent with the methodological focus and scope of the study.  These revisions overlap with AE-C1 to AE-C4 discussed above. |
| **R3-C3. Truncation levels and widening confidence intervals**:  Reviewer 3 requested clearer justification and interpretation of the 5% and 10% truncation levels, particularly why confidence intervals widen at lower truncation thresholds and how the truncation-stability analysis should be interpreted in relation to power-law behavior in the extreme tail. | We thank **Reviewer 3** for this constructive observation regarding truncation levels and confidence interval interpretation.  The revised manuscript further clarifies the bias-variance trade-off underlying tail index estimation and explains more explicitly why lower truncation levels lead to wider CIs in the deepest tail region. We also clarified the interpretation of the truncation-stability analysis in Figure 2 and emphasized that the substantial overlap of CIs across truncation levels supports the robustness of the inferred power-law behavior.  These revisions correspond primarily to AE-C6 discussed above. |
| **R3-C4. GARCH-filtered residuals and interpretation of heavy-tailedness**:  Reviewer 3 requested that the robustness analysis based on GARCH(1,1)-filtered residuals be discussed in the main text rather than only in the appendix. The reviewer further requested clarification regarding whether the observed heavy-tailedness primarily reflects volatility clustering and conditional heteroskedasticity or broader unconditional distributional properties. | We thank **Reviewer 3** for this valuable observation regarding the interpretation of heavy-tailedness and the role of volatility clustering.  We integrated the discussion of the GARCH(1,1)-filtered residual analysis into the main text and clarified the interpretation of the corresponding robustness results. The revised discussion now more clearly distinguishes between heavy-tailedness associated with conditional volatility dynamics and broader unconditional distributional properties reflected in the return series.  These revisions correspond primarily to AE-C7 discussed above. |
| **R3-C5. Recursive expanding-window estimation versus rolling-window approaches**:  Reviewer 3 requested additional discussion regarding the recursive expanding-window methodology, including sensitivity to the initial 500-observation window and the conceptual differences between recursive and rolling-window estimation approaches in the context of crisis persistence and mean reversion in tail risk. | We thank **Reviewer 3** for this helpful suggestion regarding the recursive tail analysis.  We clarified the discussion of the recursive expanding-window framework and added a brief comparison with rolling-window approaches. The revised manuscript now more clearly explains that recursive estimation retains the influence of past extreme events in subsequent estimation windows and therefore captures the persistent impact of systemic shocks on tail behavior, whereas rolling-window approaches may allow stronger mean reversion once earlier crisis observations exit the estimation window.  These revisions correspond primarily to AE-C8 discussed above. |
| **R3-C6. Implications for investors, diversification, VaR/ES estimation, and systemic risk**:  Reviewer 3 requested a deeper discussion of the implications of the empirical findings for investors, portfolio diversification, risk management, and regulatory oversight. In particular, the reviewer encouraged further discussion of the implications for mean-variance optimization, Value at Risk (VaR), Expected Shortfall (ES), crisis-period diversification benefits, contagion risk, and the interpretation of renewable energy assets as potential safe havens. | We thank **Reviewer 3** for this important observation regarding the practical implications of the empirical findings for investors, risk management, and regulatory oversight.  The revised manuscript now includes a substantially expanded discussion of the implications of heavy-tailedness and higher-moment uncertainty for portfolio diversification, mean-variance analysis, Value at Risk (VaR), Expected Shortfall (ES), stress testing, and systemic risk assessment. We additionally clarify that the empirical results do not support the interpretation of renewable energy assets as systematically safer or crisis-resilient relative to conventional energy assets from a tail-risk perspective.  These revisions overlap substantially with the broader policy and systemic-risk discussion addressed in R3-C1 and correspond primarily to AE-C1 to AE-C4 discussed above. |
| **R3-C7. Data transparency, presentation, notation, and minor comments**:  Reviewer 3 requested clarification of the exact sample end date and Bloomberg tickers, additional discussion of index weighting schemes and tail behavior, improved readability of Tables 3–5, clarification of notation and confidence interval presentation, explicit definition of “Inconcl.” in Appendix Table A4, and improved figure-caption clarity and grayscale readability. | We thank **Reviewer 3** for these helpful comments regarding data transparency, notation, and presentation clarity.  We clarified the exact sample period and Bloomberg tickers in Section 3.4 and added a brief discussion regarding index weighting schemes and tail behavior. We also refined the presentation of Tables 3-5, clarified the notation used for confidence intervals, explicitly defined “Inconcl.” in Appendix Table A4, and carefully reviewed all equations and notation throughout the manuscript for consistency.  In addition, the figure captions were revised for clarity, including explicit clarification in Figure 3 that the y-axes are standardized within each index for comparability purposes. These revisions correspond primarily to AE-C9 to AE-C11 discussed above. |

**Reviewer #4 Comments and Author Response**

We sincerely thank **Reviewer 4** for the careful reading of the manuscript and for the constructive and insightful comments provided. We appreciate the reviewer’s recognition of the methodological contribution of the study, particularly the use of CI-based tail index inference within the EVT framework.

Several of the reviewer’s observations substantially overlap with the Academic Editor(AE)’s synthesized comments (AE-C1 to AE-C11). For clarity and to avoid repetition, we grouped the reviewer’s observations into **five (5) broader thematic comment categories** and refer, where appropriate, to the corresponding AE responses where the associated revisions are discussed in greater detail.

Overall, the revised manuscript has been streamlined and strengthened in terms of policy relevance, methodological clarification, economic interpretation, and presentation consistency. Particular attention was given to the discussion of higher-order moment inference, recursive tail dynamics during the GFC and COVID-19 periods, and the broader interpretation of tail-risk similarities across renewable and conventional energy markets.

**Reviewer 4 – Overall Comment:**

The manuscript provides a comprehensive empirical analysis of heavy-tailedness in renewable and conventional energy equity indices, utilizing daily data from 2005 to 2025. The study’s primary contribution lies in its application of confidence-interval (CI)-based tail index inference, specifically using the log-log rank-size (LLRS) regression with an optimal rank shift of -1/2 to identify the existence of finite moments. While the topic is timely and the methodology is generally robust, several substantial issues must be addressed to justify publication. First, the introduction and literature review are excessively long and occasionally repetitive, particularly regarding the descriptions of the various renewable energy indices (ECO, SPGCE, ERIX, and SUN) . This background information should be more concisely integrated into the methodology or data section to improve the paper's flow. Second, while the study emphasizes that all examined indices possess finite first and second moments ($ζ > 2$) , the discussion on the potential infiniteness of third and fourth moments remains somewhat speculative. The authors should provide a more rigorous sensitivity analysis or additional statistical tests to solidify these claims, as they form the basis for the paper's warnings against skewness-based portfolio optimization. Third, the recursive analysis reveals a sharp decline in tail indices during the 2007-2008 Global Financial Crisis (GFC) , but the comparative analysis for the COVID-19 period is less detailed. Given the unique nature of the pandemic shock, a deeper exploration of why the tail risk response was "more modest" compared to the GFC would significantly enhance the discussion. Additionally, the manuscript would benefit from a clearer explanation of the economic drivers behind the observed similarities in tail behavior between renewable and conventional energy sectors, beyond just noting that they respond to systemic stress similarly. Finally, the formatting of the equations and the consistency of the notation throughout the paper need careful proofreading to ensure technical accuracy and readability. Addressing these points—conciseness in the literature review, more rigorous support for higher-order moment claims, deeper analysis of the COVID-19 period, and enhanced economic interpretation—is essential for the manuscript to meet the journal's standards.

| **Comments** | **Response** |
| --- | --- |
| **R4-C1. Streamlining the Introduction and literature review**:  Reviewer 4 noted that the Introduction and literature review were excessively long and occasionally repetitive, particularly regarding the descriptions of the renewable energy indices. The reviewer suggested integrating parts of this background material more concisely into the methodology or data sections to improve the overall flow and readability of the manuscript. | We thank **Reviewer 4** for this helpful observation regarding the structure and readability of the manuscript.  Sections 1-3 were streamlined to reduce repetition and improve the overall flow of the paper. In particular, portions of the descriptive background discussion on renewable energy indices were condensed and more closely integrated with the data and empirical framework sections where appropriate.  These revisions correspond primarily to AE-C9 discussed above. |
| **R4-C2. Higher-order moments and robustness of inference**:  Reviewer 4 expressed concern that the discussion regarding the potential infiniteness of third and fourth moments appeared somewhat speculative and requested more rigorous support and clarification regarding the CI-based inference underlying these conclusions and their implications for skewness-based portfolio optimization. | We thank **Reviewer 4** for this important observation regarding the interpretation of higher-order moments and the associated robustness of CI-based inference.  The revised manuscript now more clearly emphasizes that the discussion of third- and fourth-moment existence is based strictly on CI-based inference and is interpreted cautiously rather than as definitive evidence of moment non-existence. We also strengthened the discussion of the implications of higher-order moment uncertainty for skewness-based portfolio optimization and related risk-management applications.  These revisions correspond primarily to AE-C5 discussed above. |
| **R4-C3. GFC versus COVID-19 tail dynamics**:  Reviewer 4 requested a deeper comparative discussion of the recursive tail dynamics observed during the 2007–2008 GFC and the COVID-19 period, particularly regarding why the tail-risk response during the pandemic appeared comparatively more modest. | We thank **Reviewer 4** for this valuable suggestion regarding the comparison between the 2007-2008 GFC and the COVID-19 period.  The revised manuscript expands the discussion of the recursive tail dynamics during both episodes and provides additional interpretation regarding the comparatively more modest tail-risk response during the pandemic. In particular, we clarify the differing nature of the two shocks and discuss how the GFC represented a deeper and more persistent systemic financial disruption relative to the COVID-19 episode.  These revisions correspond primarily to AE-C4 discussed above. |
| **R4-C4. Economic interpretation of similarities**:  Reviewer 4 requested a clearer explanation of the underlying economic drivers behind the observed similarities in tail behavior between renewable and conventional energy markets beyond the general observation that both sectors respond similarly to systemic stress events. | We thank **Reviewer 4** for this important observation regarding the economic interpretation of the empirical findings.  The revised manuscript now more clearly discusses the broader macro-financial and systemic factors that may contribute to similar tail-risk dynamics across renewable and conventional energy markets, including common exposure to global financial conditions, liquidity shocks, and changes in investor risk sentiment during crisis periods.  These revisions correspond primarily to AE-C3 discussed above. |
| **R4-C5. Notation, formatting, and presentation clarity**:  Reviewer 4 requested careful proofreading of equations, notation, and formatting throughout the manuscript to improve technical consistency, readability, and overall presentation clarity. | We thank **Reviewer 4** for this helpful observation regarding notation, formatting, and presentation consistency.  We carefully reviewed all equations, notation, CI presentation, figure captions, and overall formatting throughout the manuscript to improve readability and technical consistency. We additionally refined the presentation of Tables 3-5 and clarified several figure captions and appendix notes for improved clarity.  These revisions correspond primarily to AE-C11 discussed above. |
