# Supplementary material for: Tail risk, large fluctuations and downfalls in renewable energy markets
Source: PLoS One. 2026 Jul 15;21(7):e0351106. doi: 10.1371/journal.pone.0351106 (PMC13372164; doi:10.1371/journal.pone.0351106)
Supplement: Peer review history

# Peer review: Decision Letter 0

**DOI:** 10.1371/journal.pone.0351106.r001

**Authors:** Azza Béjaoui

13 Feb 2025

PONE-D-24-59227

Tail Risk, Large Fluctuations and Downfalls in Renewable Energy Markets

PLOS ONE

Dear Dr. Ibragimov,

Thank you for submitting your manuscript to PLOS ONE. After careful consideration, we feel that it has merit but does not fully meet PLOS ONE’s publication criteria as it currently stands. Therefore, we invite you to submit a revised version of the manuscript that addresses the points raised during the review process.

==============================

**ACADEMIC EDITOR: Reviewers have now comments on your paper. You will see that they advising that you revise your paper. If you are prepared to undertake the work required, I would be pleased to review a revision.**

**In addition, please add the following issues:**

**- What are the contributions of this study? Please add them in the introduction.**

**- Add captions for Tables & Figures.**

**- Update your literature review.**

==============================

Please submit your revised manuscript by Mar 30 2025 11:59PM. If you will need more time than this to complete your revisions, please reply to this message or contact the journal office at plosone@plos.org. When you're ready to submit your revision, log on to https://www.editorialmanager.com/pone/ and select the 'Submissions Needing Revision' folder to locate your manuscript file.

Please include the following items when submitting your revised manuscript:

- A rebuttal letter that responds to each point raised by the academic editor and reviewer(s). You should upload this letter as a separate file labeled 'Response to Reviewers'.
- A marked-up copy of your manuscript that highlights changes made to the original version. You should upload this as a separate file labeled 'Revised Manuscript with Track Changes'.
- An unmarked version of your revised paper without tracked changes. You should upload this as a separate file labeled 'Manuscript'.

If you would like to make changes to your financial disclosure, please include your updated statement in your cover letter. Guidelines for resubmitting your figure files are available below the reviewer comments at the end of this letter.

If applicable, we recommend that you deposit your laboratory protocols in protocols.io to enhance the reproducibility of your results. Protocols.io assigns your protocol its own identifier (DOI) so that it can be cited independently in the future. For instructions see: https://journals.plos.org/plosone/s/submission-guidelines#loc-laboratory-protocols. Additionally, PLOS ONE offers an option for publishing peer-reviewed Lab Protocol articles, which describe protocols hosted on protocols.io. Read more information on sharing protocols at https://plos.org/protocols?utm_medium=editorial-email&utm_source=authorletters&utm_campaign=protocols.

We look forward to receiving your revised manuscript.

Kind regards,

Azza Béjaoui

Academic Editor

PLOS ONE

Journal requirements:

When submitting your revision, we need you to address these additional requirements.

1.  Please ensure that your manuscript meets PLOS ONE's style requirements, including those for file naming. The PLOS ONE style templates can be found at

https://journals.plos.org/plosone/s/file?id=wjVg/PLOSOne_formatting_sample_main_body.pdf  and

https://journals.plos.org/plosone/s/file?id=ba62/PLOSOne_formatting_sample_title_authors_affiliations.pdf

2. We note that you have indicated that there are restrictions to data sharing for this study. PLOS only allows data to be available upon request if there are legal or ethical restrictions on sharing data publicly. For more information on unacceptable data access restrictions, please see http://journals.plos.org/plosone/s/data-availability#loc-unacceptable-data-access-restrictions.

Before we proceed with your manuscript, please address the following prompts:

a) If there are ethical or legal restrictions on sharing a de-identified data set, please explain them in detail (e.g., data contain potentially identifying or sensitive patient information, data are owned by a third-party organization, etc.) and who has imposed them (e.g., a Research Ethics Committee or Institutional Review Board, etc.). Please also provide contact information for a data access committee, ethics committee, or other institutional body to which data requests may be sent.

b) If there are no restrictions, please upload the minimal anonymized data set necessary to replicate your study findings to a stable, public repository and provide us with the relevant URLs, DOIs, or accession numbers. For a list of recommended repositories, please see

https://journals.plos.org/plosone/s/recommended-repositories. You also have the option of uploading the data as Supporting Information files, but we would recommend depositing data directly to a data repository if possible.

We will update your Data Availability statement on your behalf to reflect the information you provide.

Additional Editor Comments:

What are the main contributions of this study? Please add them in the introduction.

The last paragraph of the introduction should "introduce" the remaining parts of the paper.

[Note: HTML markup is below. Please do not edit.]

Reviewers' comments:

Reviewer's Responses to Questions

**Comments to the Author**

1. Is the manuscript technically sound, and do the data support the conclusions?

The manuscript must describe a technically sound piece of scientific research with data that supports the conclusions. Experiments must have been conducted rigorously, with appropriate controls, replication, and sample sizes. The conclusions must be drawn appropriately based on the data presented.

Reviewer #1: Partly

Reviewer #2: Yes

**********

2. Has the statistical analysis been performed appropriately and rigorously?

Reviewer #1: Yes

Reviewer #2: Yes

**********

3. Have the authors made all data underlying the findings in their manuscript fully available?

The PLOS Data policy requires authors to make all data underlying the findings described in their manuscript fully available without restriction, with rare exception (please refer to the Data Availability Statement in the manuscript PDF file). The data should be provided as part of the manuscript or its supporting information, or deposited to a public repository. For example, in addition to summary statistics, the data points behind means, medians and variance measures should be available. If there are restrictions on publicly sharing data—e.g. participant privacy or use of data from a third party—those must be specified.

Reviewer #1: No

Reviewer #2: Yes

**********

4. Is the manuscript presented in an intelligible fashion and written in standard English?

PLOS ONE does not copyedit accepted manuscripts, so the language in submitted articles must be clear, correct, and unambiguous. Any typographical or grammatical errors should be corrected at revision, so please note any specific errors here.

Reviewer #1: Yes

Reviewer #2: Yes

**********

5. Review Comments to the Author

Please use the space provided to explain your answers to the questions above. You may also include additional comments for the author, including concerns about dual publication, research ethics, or publication ethics. (Please upload your review as an attachment if it exceeds 20,000 characters)

Reviewer #1: Dear Authors

It was a pleasure reading your work. It is interesting research, but it requires some improvements to be published and to maximize the article's impact.

Please find them in the attached file.

All the best

Reviewer #2: Based on its strengths and areas for improvement, I recommend the manuscript for minor revisions. Addressing the major comments will significantly enhance its clarity, relevance, and practical utility for stakeholders in renewable energy markets.

Strengths of the Paper:

The study addresses an important and timely topic, analyzing tail risks and extreme fluctuations in renewable energy markets, a crucial area for ESG investing and sustainable finance. The use of robust econometric techniques, such as the LLRS regression with optimal rank shifts and Hill's estimator, adds rigor to the heavy-tailedness analysis. The inclusion of multiple renewable energy indices, such as ECO, ERIX, SPG, and SUN, alongside a conventional energy index, provides a broad perspective for comparative analysis. The findings offer significant insights for investors, policymakers, and risk managers regarding portfolio diversification, tail risk management, and market behavior under extreme conditions.

Comments:

Comment 1: While the paper highlights the importance of renewable energy markets, the manuscript could better articulate how its findings advance existing knowledge or address gaps in the literature.

Comment 2: Clearly define the unique contribution of this study compared to previous works on tail risks in renewable energy markets.

Comment 3: While the selected indices are relevant, the manuscript could provide more justification for their inclusion. For instance, are these indices the most representative of global renewable energy markets? The analysis would benefit from explaining whether the selected indices adequately represent regional diversity (e.g., North America, Europe, Asia). Including indices from emerging markets could add value, given their growing role in renewable energy.

Comment 4: The manuscript should better explain why the LLRS regression and Hill's estimator are the most suitable methods for analyzing renewable energy indices. Comparing their advantages over alternative methodologies, such as EVT (Extreme Value Theory) or Copula-based approaches, would strengthen the study. The interpretation of tail index estimates and their implications for risk management could be more elaborated, especially for indices like SPG with pronounced heavy-tailed properties.

Comment 5: What can an investor take away from Figures 2 and 3? The manuscript should explicitly highlight the practical insights these figures provide, particularly in terms of actionable strategies, risk management, or portfolio diversification. Clarifying these points will enhance the relevance of the analysis for investors

Comment 6: The asymmetry in gain/loss behavior for renewable indices is a valuable insight but is not deeply explored. Expand the discussion on why such asymmetry exists and how it could influence investment strategies.

Comment 7: Although the study has significant implications for policymakers, the recommendations are not explicitly discussed. Add a dedicated section summarizing actionable insights for policymakers aiming to stabilize renewable energy markets.

**********

6. PLOS authors have the option to publish the peer review history of their article (what does this mean?). If published, this will include your full peer review and any attached files.

If you choose “no”, your identity will remain anonymous but your review may still be made public.

**Do you want your identity to be public for this peer review?**  For information about this choice, including consent withdrawal, please see our Privacy Policy.

Reviewer #1: No

Reviewer #2: No

**********

[NOTE: If reviewer comments were submitted as an attachment file, they will be attached to this email and accessible via the submission site. Please log into your account, locate the manuscript record, and check for the action link "View Attachments". If this link does not appear, there are no attachment files.]

While revising your submission, please upload your figure files to the Preflight Analysis and Conversion Engine (PACE) digital diagnostic tool, https://pacev2.apexcovantage.com/. PACE helps ensure that figures meet PLOS requirements. To use PACE, you must first register as a user. Registration is free. Then, login and navigate to the UPLOAD tab, where you will find detailed instructions on how to use the tool. If you encounter any issues or have any questions when using PACE, please email PLOS at figures@plos.org. Please note that Supporting Information files do not need this step.

Attachment Submitted filename: Review_to_Authors.docx

# Author comment: Author response to Decision Letter 1

**DOI:** 10.1371/journal.pone.0351106.r002

15 May 2025

We are very grateful to the Academic Editor and the reviewers for many useful comments and suggestions on the manuscript. They have helped us greatly to improve the paper. The response to the reviewers and a summary of changes in the paper following the the reviewers' and editor comments and suggestions are provided in the submitted rebuttal letter.

Attachment Submitted filename: Point-by-point Response to Reviewers.docx

# Peer review: Decision Letter 1

**DOI:** 10.1371/journal.pone.0351106.r003

**Authors:** Kyungwon Kim

8 Dec 2025

PONE-D-24-59227R1

Tail Risk, Large Fluctuations and Downfalls in Renewable Energy Markets

PLOS One

Dear Dr. Ibragimov,

Thank you for submitting your manuscript to PLOS ONE. After careful consideration, we feel that it has merit but does not fully meet PLOS ONE’s publication criteria as it currently stands. Therefore, we invite you to submit a revised version of the manuscript that addresses the points raised during the review process.

Please submit your revised manuscript by Jan 22 2026 11:59PM. If you will need more time than this to complete your revisions, please reply to this message or contact the journal office at plosone@plos.org. When you're ready to submit your revision, log on to https://www.editorialmanager.com/pone/ and select the 'Submissions Needing Revision' folder to locate your manuscript file.

Please include the following items when submitting your revised manuscript:

- A rebuttal letter that responds to each point raised by the academic editor and reviewer(s). You should upload this letter as a separate file labeled 'Response to Reviewers'.
- A marked-up copy of your manuscript that highlights changes made to the original version. You should upload this as a separate file labeled 'Revised Manuscript with Track Changes'.
- An unmarked version of your revised paper without tracked changes. You should upload this as a separate file labeled 'Manuscript'.

If you would like to make changes to your financial disclosure, please include your updated statement in your cover letter. Guidelines for resubmitting your figure files are available below the reviewer comments at the end of this letter.

If applicable, we recommend that you deposit your laboratory protocols in protocols.io to enhance the reproducibility of your results. Protocols.io assigns your protocol its own identifier (DOI) so that it can be cited independently in the future. For instructions see: https://journals.plos.org/plosone/s/submission-guidelines#loc-laboratory-protocols. Additionally, PLOS ONE offers an option for publishing peer-reviewed Lab Protocol articles, which describe protocols hosted on protocols.io. Read more information on sharing protocols at https://plos.org/protocols?utm_medium=editorial-email&utm_source=authorletters&utm_campaign=protocols.

We look forward to receiving your revised manuscript.

Kind regards,

Kyungwon Kim

Academic Editor

PLOS One

Journal Requirements:

If the reviewer comments include a recommendation to cite specific previously published works, please review and evaluate these publications to determine whether they are relevant and should be cited. There is no requirement to cite these works unless the editor has indicated otherwise.

Additional Editor Comments:

The review process for the research paper submitted to our journal has been completed. Please resubmit the paper incorporating the reviewers' comments.

Thank you for your submission. One reviewer has recommended minor revisions, but the other two reviewers have expressed significant concerns. We request substantial revisions to improve the quality of the details addressed in your manuscript. Thoroughly addressing these issues is a necessary condition for the manuscript to proceed to further review.

[Note: HTML markup is below. Please do not edit.]

Reviewers' comments:

Reviewer's Responses to Questions

**Comments to the Author**

1. If the authors have adequately addressed your comments raised in a previous round of review and you feel that this manuscript is now acceptable for publication, you may indicate that here to bypass the “Comments to the Author” section, enter your conflict of interest statement in the “Confidential to Editor” section, and submit your "Accept" recommendation.

Reviewer #1: All comments have been addressed

Reviewer #3: (No Response)

Reviewer #4: (No Response)

**********

2. Is the manuscript technically sound, and do the data support the conclusions?

The manuscript must describe a technically sound piece of scientific research with data that supports the conclusions. Experiments must have been conducted rigorously, with appropriate controls, replication, and sample sizes. The conclusions must be drawn appropriately based on the data presented.

Reviewer #1: Yes

Reviewer #3: (No Response)

Reviewer #4: (No Response)

**********

3. Has the statistical analysis been performed appropriately and rigorously?

Reviewer #1: Yes

Reviewer #3: (No Response)

Reviewer #4: (No Response)

**********

4. Have the authors made all data underlying the findings in their manuscript fully available?

The PLOS Data policy requires authors to make all data underlying the findings described in their manuscript fully available without restriction, with rare exception (please refer to the Data Availability Statement in the manuscript PDF file). The data should be provided as part of the manuscript or its supporting information, or deposited to a public repository. For example, in addition to summary statistics, the data points behind means, medians and variance measures should be available. If there are restrictions on publicly sharing data—e.g. participant privacy or use of data from a third party—those must be specified.

Reviewer #1: Yes

Reviewer #3: (No Response)

Reviewer #4: (No Response)

**********

5. Is the manuscript presented in an intelligible fashion and written in standard English?

PLOS ONE does not copyedit accepted manuscripts, so the language in submitted articles must be clear, correct, and unambiguous. Any typographical or grammatical errors should be corrected at revision, so please note any specific errors here.

Reviewer #1: Yes

Reviewer #3: (No Response)

Reviewer #4: (No Response)

**********

6. Review Comments to the Author

Please use the space provided to explain your answers to the questions above. You may also include additional comments for the author, including concerns about dual publication, research ethics, or publication ethics. (Please upload your review as an attachment if it exceeds 20,000 characters)

Reviewer #1: Dear Authors,

Thank you for your thoughtful and detailed revision of the manuscript. You have addressed all comments comprehensively, and the overall quality of the manuscript has improved significantly.

Yet, there is a minor issue that needs your attention:

Please, remove the sentence “We thank the reviewer for highlighting the importance of the analysis of heavy-tailedness of indices from emerging markets, given their growing role in the renewable energy sector. A study in this direction will be the focus of future research.” (page 15, lines 397-399)” from the main body of the manuscript. Once corrected, I believe the manuscript will be ready for publication.

All the best!

Reviewer #3: Recommendation: Major Revision

Overall Assessment:

This paper presents a timely and relevant empirical investigation into the heavy-tailedness and tail risk properties of major renewable energy equity indices, comparing them with a conventional energy benchmark. The topic is of significant importance to investors, portfolio managers, and policymakers given the rapid growth and integration of renewable assets into global financial markets. The study employs robust methodologies, specifically the log-log rank-size (LLRS) regression with an optimal shift parameter (-1/2) and Hill's estimator, to analyze daily return data from 2005 to 2025. The core finding—that renewable energy indices exhibit heavy tails but are generally less heavy-tailed than the conventional energy index, with finite first and second moments but likely infinite higher moments—is a valuable contribution to the literature on clean energy finance and financial risk management.

The paper is generally well-structured, the literature review is comprehensive, and the methodological choice is justified based on recent advances in tail index estimation. The application of a recursive window approach to observe the dynamic evolution of tail risk through crises (2008 GFC, 2020 COVID-19 shock) is a notable strength.

However, the manuscript, in its current form, requires substantial revisions before it can be considered for publication. The major concerns revolve around the depth of methodological justification, the interpretation and economic contextualization of results, robustness checks, and the overall narrative flow. Several analytical claims require more rigorous statistical support and clearer exposition. The following report details major and minor concerns that must be addressed to strengthen the manuscript significantly.

1. Major Concerns

1.1. Methodological Robustness and Justification:

While the authors correctly highlight the advantages of the LLRS regression with a -1/2 shift (Gabaix & Ibragimov, 2011) over the standard Hill estimator in the presence of dependence and small-sample bias, the application and validation of this method within the specific context of renewable energy returns need deeper exposition.

• Threshold Selection (k or n): The choice of the tail truncation level (5% and 10%) is stated to be supported by Hill-type plots (Fig 2) and references to prior literature. However, Figure 2, as described, shows stability and overlapping CIs but does not convincingly demonstrate a clear "flat" region (a stability plateau) which is the standard diagnostic for appropriate threshold selection in tail index estimation. The authors should provide a more formal analysis for threshold selection. They could employ widely used diagnostic plots like the Hill plot, the alt-plot, or the mean excess plot for the absolute returns of each index. A discussion on how the chosen thresholds (250-500+ observations) are sufficiently large for asymptotic approximations while not encroaching into the central part of the distribution is necessary. Sensitivity analysis using a range of thresholds (e.g., from 2.5% to 15% in finer increments) and reporting how the tail index estimates and their standard errors evolve would greatly enhance robustness.

• Dependence and Volatility Clustering: The authors rightly note that financial returns exhibit volatility clustering and that the LLRS method with the optimal shift is robust to dependence. However, they do not demonstrate this property for their dataset. A key question is whether the extreme returns used in the estimation (the upper 5%/10% of absolute returns) themselves exhibit significant temporal dependence. Simple tests for autocorrelation in the extremes or in squared extremes could be presented. If significant dependence is found, the authors should discuss its potential impact even on the "robust" LLRS method and whether techniques like block bootstrapping for standard errors (as an additional check) were considered and why they were or were not employed.

• Goodness-of-Fit to Power Law: The entire analysis hinges on the assumption that the tails of the return distributions follow a power law (Pareto-type tail). The authors should provide more formal goodness-of-fit tests for the power-law hypothesis. Visual Pareto quantile plots (QQ-plots against the exponential distribution) for the log-transformed exceedances are a standard tool. Statistical tests, such as the Kolmogorov-Smirnov test adapted for Pareto distributions or the Clauset-Shalizi-Newman test, should be applied and reported. This is crucial because if the tails deviate significantly from a power law, the interpretation of the tail index ζ loses its precise meaning regarding the finiteness of moments.

1.2. Data and Sample Representativeness:

• Index Composition and Heterogeneity: The analysis treats indices (ECO, ERIX, SPG, SUN, DJUSEN) as monolithic entities. However, these indices have vastly different compositions. ECO is US-focused and equal-weighted, SPG is global and presumably market-cap weighted, SUN is sector-specific (solar). DJUSEN is a US oil & gas index. This heterogeneity is a double-edged sword: it provides diversity but complicates direct comparison. The finding that SPG (global) has the smallest tail index (heaviest tail) is attributed to its inclusion of emerging market companies. This is a plausible hypothesis but remains untested. The authors should attempt to control for or discuss this factor more deeply. Could the heavier tail of SPG be due to its weighting scheme, its sectoral breadth (beyond solar), or indeed its geographic exposure? A correlation analysis between the tail index estimates and index characteristics (e.g., average market cap, geographic concentration Herfindahl index, sector diversity) across a broader set of indices (if data were available) would be insightful. As it stands, the conclusion is suggestive but not conclusive.

• Time Period and Structural Breaks: The sample covers 2005-2025, a period encompassing multiple regimes: pre-GFC boom, GFC, post-GFC recovery, the shale revolution (impacting DJUSEN), the COVID-19 crash, and the recent energy crisis. The recursive window analysis in Section 4.2 is excellent for showing dynamics but the full-sample tail index estimates presented in Table 3 may be an average over these heterogeneous periods. The authors should consider conducting a formal test for structural breaks in the tail index itself, using methods such as the Quintos, Fan, and Phillips (2001) test or similar approaches referenced in their literature review (e.g., Xing & Ibragimov, 2023). Identifying breakpoints (e.g., around 2008, 2020) and reporting sub-period tail indices would provide a richer narrative. For instance, is the "less heavy-tailed" result for renewables stable across all sub-periods, or is it a feature of the post-GFC era?

• Benchmark Choice and Omitted Comparisons: Using DJUSEN (US Oil & Gas) as the sole conventional energy benchmark is reasonable but narrow. The risk profile of a global integrated energy index (e.g., MSCI World Energy) might differ. More critically, to truly assess the "alternative" nature of renewables, a comparison with broad equity market indices (e.g., S&P 500, MSCI World) is almost mandatory. The claim that renewables are a "relatively lower-risk investment alternative" (Abstract) is made relative to dirty energy. How do their tail risks compare to the overall equity market? This is a key question for portfolio managers considering renewables for diversification. Adding this comparison would significantly broaden the paper's impact.

1.3. Interpretation of Findings and Statistical Significance:

• The "Less Heavy-Tailed" Conclusion: This is a central finding. However, the authors note that the confidence intervals for the tail indices of renewable and conventional indices overlap, and thus the differences are "not statistically significant" (pp. 19, 29). This is a critical caveat that is underemphasized. The abstract and conclusion sections (pp. 1, 28-29) strongly state that renewables are less heavy-tailed, while the results section correctly highlights the lack of statistical significance. This creates a contradiction. The narrative must be consistently cautious. The paper should clearly state: Point estimates suggest renewable indices (except SPG) have larger tail indices than DJUSEN, but these differences are not statistically distinguishable at conventional levels. The economic implication—that investors cannot reliably conclude that renewables have significantly lighter tails than oil/gas based on this data—must be front and center. The discussion on pp. 27-28 ("aligns with existing literature that renewable energy assets are less riskier") should be tempered accordingly.

• Inference on Higher Moments: The discussion about infinite third and fourth moments (pp. 20, 26-27, 29) is based on hypothesis tests (ζ=3, ζ=4). The authors must report the actual p-values for these tests, not just rejection/non-rejection statements. More importantly, they must discuss the economic and practical significance of an "infinite" third or fourth moment. In finite samples, what we observe is "very large" skewness/kurtosis, not literal infinity. The key takeaway is that higher-moment estimates are extremely unstable and unreliable. The warning about the limitations of mean-variance-skewness (MVS) models (p. 27) is valid, but it should be framed as: "Given the extremely high and unstable estimates of higher moments, models relying on precise estimates of skewness and kurtosis are likely to be unreliable and should be used with extreme caution," rather than a definitive statement about infinities.

• Gain/Loss Asymmetry Analysis (Tables 4 & 5): The analysis of left vs. right tails is valuable. However, the interpretation is muddled. The authors find mixed patterns (e.g., ECO heavier left at 5%, heavier right at 10%) and correctly note that CIs largely overlap. They then state, "Overall, the results... suggest that significant downfalls... tend to be somewhat more pronounced than their significant upward moves" (p. 22). This summary seems to lean on the point estimates for SPG, SUN, DJUSEN and downplays the mixed/overlapping results for ECO and ERIX. A more balanced conclusion is needed: there is weak and inconsistent evidence of asymmetry across these indices, with no statistically significant left-right difference for most. This should be linked to the literature on asymmetric volatility in renewables – is the asymmetry in volatility mirrored in the tail indices? A brief discussion is warranted.

1.4. The Recursive Window Analysis (Section 4.2):

• Interpretation of Persistence: The description of the recursive method is good. However, the interpretation of the "stabilization" of tail indices post-2008 needs refinement. The authors state that extreme values from the crisis persist in the window, maintaining tail heaviness. This is technically true, but it raises a question: does the stabilized tail index (e.g., ~3 for ECO) represent the new steady-state tail behavior of the market, or is it an artifact of the permanently embedded 2008 crisis data? A rolling window analysis (with a fixed window size, e.g., 1000 days) could complement the recursive analysis. A rolling window would show whether the tail index eventually reverts to a pre-crisis level once the crisis data rolls out of the sample, or if the change is permanent. This would offer a sharper view of whether crises permanently alter the tail risk structure.

• The 2020 Event: The minimal reaction of tail indices (except DJUSEN) to the COVID-19 shock is an interesting finding. The authors provide two interpretations (p. 24). They should add a third, methodological, possibility: the 2008 crisis was so extreme that it dominates the tail of the distribution. The 2020 shock, while severe, may not have generated observations that were more extreme than the top 5% or 10% already occupied by 2008 data, thus leaving the tail index estimate unchanged. This speaks to the "persistence" feature they described. Analyzing the entry and exit of specific extreme dates into/from the top *n* observations in the recursive window could visually demonstrate this.

1.5. Discussion and Implications Section:

• Connecting Tail Indices to Practical Risk Metrics: Section 5 begins well by linking heavy-tailedness to model robustness. However, it stops short of demonstrating the quantitative implications for risk management. The authors should include a small, illustrative calculation. For example, using the estimated tail index ζ for ECO and a parametric (Pareto) tail model, calculate the 1-day 99% Value at Risk (VaR) and Expected Shortfall (ES). Then, compare this to the VaR/ES estimated under a false assumption of normality (using sample mean and variance). This would vividly show how underestimating tail heaviness leads to a severe underestimation of risk capital. This practical demonstration would bridge the gap between the econometric results and the paper's stated audience of risk managers and investors.

• Portfolio Implications: The discussion on portfolio theory (pp. 26-27) is good but could be more specific. Given the finding of finite variance, mean-variance analysis is theoretically justified. But what does the potential infinite higher moments imply for popular empirical practices? For instance, many portfolio optimizers use historical return samples, which will produce finite but wildly unstable estimates of skewness and kurtosis. The practical advice should be: "While mean-variance frameworks are applicable, attempting to optimize portfolios based on historical higher-moment estimates (e.g., for skewness-seeking strategies) in renewable energy markets is likely to lead to erratic and unreliable portfolio weights due to the extreme estimation error." This is a stronger, more practical warning than just stating MVS may be "limited."

• Overstatement of Certain Conclusions: Some statements are too broad. E.g., p. 28: "provides investors with a further incentive to substitute their holdings in dirty energy assets with renewable energy assets." This is an investment recommendation that goes beyond the scope of the risk analysis presented. The paper shows comparable or possibly slightly milder tail risk, but does not analyze expected returns, correlations, or other diversification benefits. The conclusion should stick to the risk dimension: "From a tail risk perspective, our findings do not present an obstacle and may provide mild support for considering renewable energy assets as a substitute for conventional energy holdings within a diversified portfolio."

2. Minor Concerns

2.1. Clarity and Exposition:

• The paper is lengthy and can be verbose. Some sections, particularly the literature review, could be tightened without losing essential information. The introduction effectively sets the stage, but the transition to the paper's specific contributions could be sharper.

• Abstract: The abstract states the study uses data "from 2005 to 2025." As of the current date (presumably 2024 or early 2025), this is a future date. It should read "through [last available date]" or "covering the period from 2005 to [specific date in 2024/2025]."

• Defining "Heavy-Tailedness": The definition in Section 2.3 is technically correct but could be made more accessible early on. A simple intuitive explanation (e.g., "higher probability of extreme crashes and booms compared to the normal distribution") in the introduction would help a broader audience.

2.2. Figures and Tables:

• Figure 1 (Return Plots): This figure is essential but as described, it seems to be a simple time-series plot. Consider adding horizontal lines marking the +/− thresholds that correspond to the 5% and 10% tails. This would visually connect the return series to the extreme events used in estimation.

• Tables 3, 4, 5: These are dense. Consider using a format that highlights key comparisons. For example, in Table 3, use bold or shading to indicate the smallest tail index (SPG) and the largest (ECO at 5%). A summary table presenting the moment existence conclusions (Finite/Infinite for 1st, 2nd, 3rd, 4th) for each index based on both methods would be extremely helpful for the reader.

• Figures 3 & 4 (Recursive Plots): Ensure the y-axis scales are consistent across subplots to facilitate visual comparison of the magnitude of drops across indices. Clearly label the 2008 and 2020 periods with shaded vertical bands.

2.3. Literature Review and Context:

• The literature review is comprehensive but could be better synthesized to directly motivate the paper's gap. Section 2.1 adequately covers linkages (oil, tech) and risks (policy). Section 2.2 on indices is useful. However, the transition from the general literature to the specific gap on tail index estimation in renewable markets should be more forceful. The paragraph starting at line 101 does this, but it could be placed more prominently.

• The review of heavy-tailedness methods (2.3) is good. Ensure all key methodological references (Gabaix & Ibragimov, 2011; Embrechts et al., 1997; etc.) are consistently cited.

2.4. Technical Details:

• Data Transformation: The authors use simple returns rather than log returns due to "potential outliers" (p. 15). This is acceptable, but a brief justification in the context of tail estimation would be helpful. Do log returns dampen extreme values in a way that could bias tail index estimates? A sentence or two would suffice.

• Stationarity (ADF Test): The results mentioned on p. 18 should be moved to a footnote or a brief paragraph in the main text. Simply stating "The return series are confirmed to be stationary via ADF tests (results available upon request)" is sufficient for the main flow.

• Software and Code: For reproducibility, the authors should state the statistical software used (e.g., R, MATLAB, Python) and consider making their code available as part of the submission's supplementary materials, if the journal allows.

3. Recommendation

This paper addresses an important topic with appropriate and sophisticated methodology. The core results are interesting and have clear relevance. However, as detailed above, significant revisions are required to solidify the methodological foundations, clarify the interpretation (especially regarding statistical significance), contextualize the findings within a broader financial landscape, and draw more nuanced and practical conclusions.

I recommend Major Revision. The authors have the opportunity to transform a good paper into an excellent one by addressing these concerns. The revisions should focus on:

1. Enhancing methodological transparency and robustness checks (threshold selection, goodness-of-fit, dependence).

2. Re-framing results with consistent emphasis on statistical uncertainty.

3. Deepening the economic and practical interpretation of findings, including illustrative risk calculations and broader benchmark comparisons.

4. Improving the narrative flow and precision of language, particularly in the abstract and conclusions.

Reviewer #4: Overall Assessment

This manuscript addresses a relevant and timely topic: the tail risk characteristics of renewable energy stock indices in comparison to conventional energy indices. The authors employ a robust econometric framework—specifically, log–log rank–size (LLRS) regressions with optimal rank shifts and valid standard errors—to analyze the heavy-tailedness of return distributions from five major energy-sector indices over a 20-year period (2005–2025). The paper makes a valuable contribution to the literature by focusing on the often-overlooked extremal behavior and higher-moment properties of renewable energy returns, using a methodologically sound approach grounded in recent advances in tail index estimation.

The motivation is compelling. As global capital flows increasingly into ESG-compliant and sustainable assets, understanding the true downside risk profile of such investments is crucial for investors, risk managers, and policymakers. The authors correctly emphasize that the assumption of Gaussianity or even finite fourth moments may be misleading in real-world renewable energy markets—a point that merits greater attention in both academic and applied finance circles.

Nonetheless, despite its strengths, the paper suffers from several substantive methodological, interpretive, and structural shortcomings that collectively prevent it from being acceptable in its current form. I recommend major revision. Below, I provide a detailed, point-by-point critique organized by section. The comments aim not only to identify weaknesses but also to guide the authors toward a stronger, more rigorous, and publishable manuscript.

________________________________________

1. Introduction

The introduction effectively contextualizes the study within the climate-policy and decarbonization discourse. However, it suffers from redundancy and over-quotation of background facts (e.g., CO₂ emission statistics) that distract from the core econometric contribution. More importantly, the novelty claim is insufficiently articulated.

• The abstract and introduction state that “heavy-tailedness and tail risk in renewable energy markets remain underexplored,” but this assertion is not rigorously defended. Recent works by Ji et al. (2018), Reboredo (2015), and Ghabri et al. (2021)—all cited in the paper—have examined extreme dependence and downside risk in clean energy markets using CoVaR, copulas, and wavelet methods. These studies do engage with tail behavior, albeit not via tail index estimation. The authors must more precisely delineate their contribution: not that tail risk is unexplored, but that the specific question of the existence and order of finite moments via tail index estimation has not been systematically addressed. This distinction is crucial.

• The introduction also conflates volatility (second moment) with tail risk (extremal behavior beyond variance). For instance, the sentence “clean energy stocks’ volatility is typically greater than traditional energy stocks” (lines 83–84) is used to motivate a study of heavy tails—but higher volatility does not necessarily imply heavier tails (e.g., a scaled Gaussian has high volatility but no heavy tails). The authors must clarify that their interest lies not in conditional variance dynamics (GARCH-type) but in the unconditional extremal decay rate of the return distribution.

• Finally, the paper mentions the sample up to February 2025, which is in the future relative to the current date (2024). This raises concerns about data validity. Either this is a typographical error (should be 2024), or the authors are using simulated or forecasted data beyond the present, which must be explicitly disclosed and justified.

________________________________________

2. Literature Review

The literature review is extensive but somewhat disorganized. It falls into three subsections, but the connections between them are weak.

2.1 Renewable Energy Markets

This subsection surveys a broad range of papers on oil–renewables linkages, policy risk, and performance, but it lacks thematic focus. The authors should restructure this section to highlight only studies that are directly relevant to tail risk, extremal dependence, or distributional properties (e.g., skewness, kurtosis, tail dependence). Papers discussing mean–variance performance or general volatility spillovers are less relevant and dilute the narrative.

Moreover, the claim that “renewable energy firms tend to over-perform in the long run” (lines 229–230) is presented as consensus, but this is contested in the literature (e.g., Rezec & Scholtens, 2017, cited as [53], actually argue that renewable investments are unattractive due to modest risk-adjusted returns). The authors should acknowledge this heterogeneity in empirical findings rather than presenting a monolithic view.

2.2 Renewable Energy Indices

This subsection correctly describes the indices, but it misses an opportunity to critically evaluate their representativeness. For example:

• ECO and DJUSEN are U.S.-centric; SPG is global; ERIX is European. How does this geographic heterogeneity affect cross-index comparability?

• SUN is sector-specific (solar), while others are broad clean energy. Does solar exhibit structurally different tail behavior due to technology cycles or policy shocks (e.g., tariff changes, silicon shortages)?

These differences are later alluded to (e.g., SPG’s heavier tails attributed to emerging market exposure), but the conceptual groundwork should be laid here.

2.3 Heavy-Tailedness and Inference

This is the strongest part of the literature review. However, the authors overlook a critical debate in the extreme value theory (EVT) community: the choice of tail threshold. While they justify using 5% and 10% based on prior studies, they do not engage with the diagnostic tools available (e.g., mean excess plots, stability of Hill estimator across k) to validate this choice for their specific data. Moreover, the assumption of a pure power law (Pareto tail) is rarely satisfied in financial returns, which often exhibit subexponential but non-Pareto tails (e.g., lognormal, stretched exponential). The robustness of LLRS to such deviations is claimed (citing Gabaix & Ibragimov, 2011), but this should be empirically verified (e.g., via goodness-of-fit tests or comparison with alternative tail models).

________________________________________

3. Methodology and Data

3.1–3.4: Methodological Concerns

The paper’s core methodological innovation—LLRS with γ = −1/2—is well motivated. However, several issues arise:

1. Return Calculation: The authors state they use “daily simple returns as the percentage change… more appropriate than log returns due to the presence of potential outliers” (lines 409–411). This is incorrect. Simple returns can exceed −100%, leading to undefined log returns, but in practice, for stock indices, this is extremely rare. More importantly, log returns are additive over time and are standard in tail analysis because EVT asymptotics are typically derived for i.i.d. sequences, and log returns better approximate this. The use of simple returns may introduce bias in the tail, especially for large negative moves. The authors must justify this choice more rigorously or switch to log returns.

2. Tail Index Estimation: The paper uses both LLRS and Hill estimators but relies primarily on LLRS. However, it does not address a key limitation: both methods assume i.i.d. data, yet financial returns exhibit volatility clustering and serial dependence. While Gabaix & Ibragimov (2011) argue LLRS is robust to GARCH-type dependence, this is only asymptotically true under specific conditions. The authors should:

o Test for serial dependence in absolute/ squared returns (e.g., Ljung–Box).

o Consider blocking methods or subsampling to account for dependence (e.g., Hall et al., 1998).

o At minimum, report results using filtered returns (e.g., standardized residuals from a GARCH(1,1) model) to isolate the tail behavior of innovations.

3. Confidence Intervals: The CIs for LLRS assume asymptotic normality of the OLS estimator. However, with n as small as 250 (5% of 5000), and given potential deviations from power laws, these CIs may be unreliable. The authors should consider bootstrap CIs (e.g., percentile-t bootstrap) to assess finite-sample accuracy.

4. Recursive Estimation: The recursive window approach (Section 4.2, Figs 3–4) is useful for detecting structural breaks. However, the window starts at 500 observations—arbitrary without justification. Why not test sensitivity to initial window size (e.g., 300 vs. 700)? Moreover, recursive estimates are highly autocorrelated, so the apparent “stability” post-2008 may be an artifact of persistence, not true stationarity.

Data Concerns

• Sample Period: The paper claims data up to February 2025. Unless this is a typo, this is problematic. If real data exist beyond mid-2024, the authors must clarify the source and vouch for its reliability. If it includes forecasts or simulated data, this must be disclosed, as it invalidates tail inference.

• Index Definitions: Table 1 states SPG “aims to include 100 constituents,” but the current S&P Global Clean Energy Index actually has ~30 constituents. This discrepancy suggests the authors may be using an outdated or modified version. This must be clarified.

• Stationarity: The ADF results (mentioned in passing) confirm return stationarity, which is standard. But for tail inference, strict stationarity is required. The recursive plots show clear non-stationarity in tail behavior (e.g., 2008 crash). The authors should discuss whether their pooled tail estimates mask important time-varying dynamics.

________________________________________

4. Results

4.1 Return Plots and Summary Statistics

Figure 1 is helpful but lacks quantitative annotations (e.g., vertical lines for crisis dates). Table 2 shows high kurtosis for all indices, consistent with heavy tails. However, kurtosis is a poor indicator of tail heaviness in small samples or under infinite fourth moments—precisely the issue the paper studies. The authors should de-emphasize kurtosis and focus on tail index estimates.

4.2 Tail Risk and Tail Index Estimates

This is the core of the paper, but interpretation is inconsistent.

• Point Estimates vs. CIs: The authors repeatedly compare point estimates (e.g., “SPG has the smallest tail index”) but then note that CIs overlap, implying no statistical difference. This is contradictory. If differences are not significant, they should not be emphasized. The conclusion that “renewable energy indices are less heavy-tailed than conventional” (Abstract) is not supported by statistical tests—only by point estimates. A formal test (e.g., bootstrap difference in tail indices) is needed.

• Moment Existence: The inference that “all renewable indices have finite second moments” relies on ζ̂ > 2. But at 5% truncation, the lower bound of the CI for SPG (LLRS) is 2.34—comfortably above 2. However, at 10%, it’s 2.32, still fine. But what if the true ζ is 1.99? The authors assume the point estimate is the truth, but hypothesis testing should be based on CIs. For example, to claim “finite variance,” one must show the entire CI lies above 2. For SPG10% (LLRS), 2.32 > 2, so OK—but for emerging market indices not included, this may fail. The authors are careful here, but the language could be tightened.

• Asymmetry (Tables 4–5): The gain/loss asymmetry analysis is valuable. However, the authors note that CIs for left/right tails overlap, yet still claim “downward moves… are somewhat more pronounced.” This is misleading. Without a significant difference, such statements should be qualified as directional tendencies, not established facts.

• Recursive Results (Figs 3–4): The drop in tail index during 2008 is dramatic, especially for SPG (below 2). This implies infinite variance during crises—a critical finding that deserves more discussion. Yet the authors treat it as a transient shock. They should explore:

o The duration of sub-2 tail indices.

o Whether such episodes invalidate standard risk models during crises, even if normality resumes later.

o Comparison with other crises (e.g., 2020, Ukraine war)—why was 2020 milder?

________________________________________

5. Discussion

This section is somewhat repetitive of results and lacks depth on key implications.

• Risk Management: The authors argue VaR/ES are “appropriate” because ζ > 1. But VaR is incoherent for ζ ≤ 1, while ES is coherent only if ζ > 1 (Embrechts et al., 1997). Since all ζ̂ > 2, ES is fine—but the authors should cite the precise conditions from risk measure theory.

• Portfolio Theory: The discussion of Markowitz and MVS portfolios is relevant but superficial. If ζ ≈ 3, then skewness may be unstable (infinite third moment), making MVS optimization numerically unreliable. The authors should cite empirical studies showing MVS failure under heavy tails (e.g., Jondeau & Rockinger, 2006).

• Policy Implications: The claim that renewables are “lower-risk” could encourage overallocation to green assets. But if tail risk is underestimated (e.g., due to climate policy shocks), this could backfire. The authors should discuss this moral hazard.

________________________________________

6. Conclusion

The conclusion reiterates findings but misses an opportunity to outline a research agenda. Future work should include:

• Multivariate tail dependence (e.g., via copulas).

• Real-time tail monitoring systems for renewable portfolios.

• Integration with climate stress testing (e.g., NGFS scenarios).

________________________________________

Other Issues

• Writing and Clarity: The manuscript suffers from grammatical errors, inconsistent notation (e.g., ζ vs. α for tail index), and formatting issues (e.g., line breaks in equations). Professional copyediting is advised.

• Figures: Figures 1–4 are referenced but not included in the PDF. Without them, assessing the recursive results is difficult. Ensure figures are embedded or provided separately.

• Reproducibility: No code or data availability statement is provided. PLOS ONE requires this. The authors should commit to sharing replication files.

• References: Some references are incomplete (e.g., [39] lacks journal name in the main text). Ensure all follow a consistent style.

________________________________________

Recommendations for Revision

1. Clarify the novelty: Emphasize the moment-existence angle, not just “tail risk.”

2. Address data validity: Confirm whether 2025 data are real or simulated.

3. Improve methodology:

o Justify return type.

o Address dependence in tail estimation.

o Use formal tests for tail index differences.

4. Reinterpret results cautiously: Avoid overclaiming where CIs overlap.

5. Deepen discussion: Link findings to risk measure theory, portfolio practice, and climate policy.

6. Enhance transparency: Provide data/code, fix figures, improve writing.

**********

7. PLOS authors have the option to publish the peer review history of their article (what does this mean?). If published, this will include your full peer review and any attached files.

If you choose “no”, your identity will remain anonymous but your review may still be made public.

**Do you want your identity to be public for this peer review?**  For information about this choice, including consent withdrawal, please see our Privacy Policy.

Reviewer #1: No

Reviewer #3: No

Reviewer #4: No

**********

[NOTE: If reviewer comments were submitted as an attachment file, they will be attached to this email and accessible via the submission site. Please log into your account, locate the manuscript record, and check for the action link "View Attachments". If this link does not appear, there are no attachment files.]

To ensure your figures meet our technical requirements, please review our figure guidelines: https://journals.plos.org/plosone/s/figures

You may also use PLOS’s free figure tool, NAAS, to help you prepare publication quality figures: https://journals.plos.org/plosone/s/figures#loc-tools-for-figure-preparation.

NAAS will assess whether your figures meet our technical requirements by comparing each figure against our figure specifications.

# Author comment: Author response to Decision Letter 2

**DOI:** 10.1371/journal.pone.0351106.r004

9 Mar 2026

Please see the attached Response to reviewers file submitted.

Attachment Submitted filename: z_Response to Reviewer (R2)_revised_final.docx

# Peer review: Decision Letter 2

**DOI:** 10.1371/journal.pone.0351106.r005

**Authors:** Kyungwon Kim

6 Apr 2026

PONE-D-24-59227R2

Tail Risk, Large Fluctuations and Downfalls in Renewable Energy Markets

PLOS One

Dear Dr. Ibragimov,

Thank you for submitting your manuscript to PLOS ONE. After careful consideration, we feel that it has merit but does not fully meet PLOS ONE’s publication criteria as it currently stands. Therefore, we invite you to submit a revised version of the manuscript that addresses the points raised during the review process.

**ACADEMIC EDITOR:**

As the Academic Editor, I have carefully evaluated your revised manuscript along with the feedback from the three reviewers. While Reviewer #1 is satisfied with your previous revisions, Reviewers #3 and #4 have raised substantial concerns that must be addressed. Both reviewers agree that the empirical analysis is robust and timely; however, they require deeper economic contextualization, stronger methodological justification, and improvements to the manuscript's structure.

Therefore, I am recommending a Major Revision. Please ensure that your next submission comprehensively addresses the following key areas:

<h3 data-path-to-node="6">1. Economic Context and Policy Implications</h3>

- Real-Economy Linkages: Broaden your motivation to explicitly connect financial tail risk with the socio-economic goals of the global energy transition. Discuss how high tail risk impacts the cost of capital, infrastructure deployment, and climate targets.
- Systemic and Regulatory Implications: Elaborate on the implications of your findings for policymakers and regulators. Specifically, address how the potential for infinite higher moments affects standard risk management tools (e.g., mean-variance optimization, VaR, ES estimation risks) and regulatory stress testing.
- Economic Drivers: Provide a clearer explanation of the underlying economic drivers that cause renewable and conventional energy sectors to exhibit similar tail behaviors and responses to systemic stress.
- Crisis Comparison: Deepen your analysis regarding the COVID-19 pandemic versus the 2007-2008 Global Financial Crisis. Explain why the tail risk response was notably more modest during the pandemic.

<h3 data-path-to-node="8">2. Methodological Rigor and Robustness</h3>

- Higher-Order Moments: Provide more rigorous sensitivity analyses or additional statistical tests to solidify your claims regarding the potential infiniteness of third and fourth moments, as these claims currently appear speculative.
- Truncation Levels: Justify the choice of 5% and 10% truncation levels. Explicitly discuss why confidence intervals widen significantly at the 5% level and what this suggests about power-law behavior in the extreme tail.
- GARCH Filtered Residuals: Move the discussion of the GARCH(1,1) filtered residuals from the appendix to the main text. Address the discrepancy between tail index estimates for raw returns versus standardized residuals, clarifying whether the observed heavy-tailedness is driven by conditional heteroskedasticity or unconditional distributional properties.
- Recursive vs. Rolling Windows: Discuss the sensitivity of your recursive expanding-window approach to the initial 500-observation window size. Include a comparative discussion on how a rolling window might differentiate between persistent structural breaks and temporary shock absorption.

<h3 data-path-to-node="10">3. Structure, Presentation, and Clarity</h3>

- Streamline Introduction: Condense the excessively long introduction and literature review. Specifically, integrate the background descriptions of the renewable energy indices more concisely into the Data or Methodology sections.
- Data Clarification: Explicitly clarify the end date of your dataset (stated as February 2025). Ensure this is accurate, provide exact cut-off dates, and include Bloomberg terminal codes for full replicability. Briefly comment on how index weighting schemes (equal-dollar vs. market-cap) might influence tail properties.
- Formatting and Readability: Improve the readability of Tables 3, 4, and 5 by clearly separating confidence intervals from point estimates. Ensure Figure 1 Panel B is legible in grayscale, and state the standardized scale in Figure 3's caption. Carefully proofread all equations and ensure consistent notation throughout the manuscript.

Please provide a detailed, point-by-point response to all reviewer comments in your rebuttal letter.

Please submit your revised manuscript by May 21 2026 11:59PM. If you will need more time than this to complete your revisions, please reply to this message or contact the journal office at plosone@plos.org. When you're ready to submit your revision, log on to https://www.editorialmanager.com/pone/ and select the 'Submissions Needing Revision' folder to locate your manuscript file.

Please include the following items when submitting your revised manuscript:

- A letter that responds to each point raised by the academic editor and reviewer(s). You should upload this letter as a separate file labeled 'Response to Reviewers'.
- A marked-up copy of your manuscript that highlights changes made to the original version. You should upload this as a separate file labeled 'Revised Manuscript with Track Changes'.
- An unmarked version of your revised paper without tracked changes. You should upload this as a separate file labeled 'Manuscript'.

If you would like to make changes to your financial disclosure, please include your updated statement in your cover letter. Guidelines for resubmitting your figure files are available below the reviewer comments at the end of this letter.

If applicable, we recommend that you deposit your laboratory protocols in protocols.io to enhance the reproducibility of your results. Protocols.io assigns your protocol its own identifier (DOI) so that it can be cited independently in the future. For instructions see: https://journals.plos.org/plosone/s/submission-guidelines#loc-laboratory-protocols. Additionally, PLOS ONE offers an option for publishing peer-reviewed Lab Protocol articles, which describe protocols hosted on protocols.io. Read more information on sharing protocols at https://plos.org/protocols?utm_medium=editorial-email&utm_source=authorletters&utm_campaign=protocols.

As the corresponding author, your ORCID iD is verified in the submission system and will appear in the published article. PLOS supports the use of ORCID, and we encourage all coauthors to register for an ORCID iD and use it as well. Please encourage your coauthors to verify their ORCID iD within the submission system before final acceptance, as unverified ORCID iDs will not appear in the published article. *Only*  the individual author can complete the verification step; PLOS staff *cannot*  verify ORCID iDs on behalf of authors.

We look forward to receiving your revised manuscript.

Kind regards,

Kyungwon Kim

Academic Editor

PLOS One

Journal Requirements:

If the reviewer comments include a recommendation to cite specific previously published works, please review and evaluate these publications to determine whether they are relevant and should be cited. There is no requirement to cite these works unless the editor has indicated otherwise.

[Note: HTML markup is below. Please do not edit.]

Reviewers' comments:

Reviewer's Responses to Questions

**Comments to the Author**

1. If the authors have adequately addressed your comments raised in a previous round of review and you feel that this manuscript is now acceptable for publication, you may indicate that here to bypass the “Comments to the Author” section, enter your conflict of interest statement in the “Confidential to Editor” section, and submit your "Accept" recommendation.

Reviewer #1: All comments have been addressed

Reviewer #3: (No Response)

Reviewer #4: (No Response)

**********

2. Is the manuscript technically sound, and do the data support the conclusions?

The manuscript must describe a technically sound piece of scientific research with data that supports the conclusions. Experiments must have been conducted rigorously, with appropriate controls, replication, and sample sizes. The conclusions must be drawn appropriately based on the data presented.

Reviewer #1: Yes

Reviewer #3: (No Response)

Reviewer #4: (No Response)

**********

3. Has the statistical analysis been performed appropriately and rigorously?

Reviewer #1: Yes

Reviewer #3: (No Response)

Reviewer #4: (No Response)

**********

4. Have the authors made all data underlying the findings in their manuscript fully available?

The PLOS Data policy requires authors to make all data underlying the findings described in their manuscript fully available without restriction, with rare exception (please refer to the Data Availability Statement in the manuscript PDF file). The data should be provided as part of the manuscript or its supporting information, or deposited to a public repository. For example, in addition to summary statistics, the data points behind means, medians and variance measures should be available. If there are restrictions on publicly sharing data—e.g. participant privacy or use of data from a third party—those must be specified.

Reviewer #1: Yes

Reviewer #3: (No Response)

Reviewer #4: (No Response)

**********

5. Is the manuscript presented in an intelligible fashion and written in standard English?

PLOS ONE does not copyedit accepted manuscripts, so the language in submitted articles must be clear, correct, and unambiguous. Any typographical or grammatical errors should be corrected at revision, so please note any specific errors here.

Reviewer #1: Yes

Reviewer #3: (No Response)

Reviewer #4: (No Response)

**********

6. Review Comments to the Author

Please use the space provided to explain your answers to the questions above. You may also include additional comments for the author, including concerns about dual publication, research ethics, or publication ethics. (Please upload your review as an attachment if it exceeds 20,000 characters)

Reviewer #1: Dear Authors,

Thank you for your detailed revision of the manuscript.

You have addressed all comments comprehensively.

All the best!

Reviewer #3: Recommendation: Revision

The manuscript presents a timely and empirically rigorous analysis of tail risk and heavy-tailed behavior in renewable energy equity markets compared to conventional energy benchmarks. The authors employ robust econometric techniques, specifically the Log-Log Rank-Size (LLRS) regression with optimal rank shift and Hill's estimator, to assess the existence of finite moments across five major energy indices. The use of a recursive expanding-window approach to capture time-varying tail risk during systemic stress events, such as the Global Financial Crisis and the COVID-19 pandemic, adds significant value to the existing literature. The finding that renewable energy indices exhibit heavy tails similar to conventional energy assets challenges the notion that green assets are inherently safer from an extreme risk perspective. However, while the statistical analysis is sound, the manuscript requires substantial revisions to better contextualize these financial findings within the broader socio-economic and policy frameworks governing the energy transition. The discussion on implications for investors and policymakers needs deepening, and the motivation section requires strengthening to align with broader sustainability goals. Consequently, I recommend a Major Revision to address the following concerns.

Comments:

The current introduction effectively outlines the financial motivation for studying tail risk, focusing on volatility, decarbonization goals, and investor exposure. However, the manuscript would benefit significantly from a more robust connection between financial tail risk and the broader objectives of the global energy transition. Financial stability in renewable energy markets is not an isolated metric; it is a prerequisite for the sustained capital flow required to meet climate targets. Therefore, the authors should expand the motivation section to explicitly link statistical tail behavior with the real-economy implications of energy investment. Enhance the motivation for your research by drawing on pertinent literature that emphasizes the pivotal role of your field in advancing socio-economic gains (doi: 10.1142/S3082844925500174; 10.1515/jafio-2016-0006) within the wider framework of energy/environmental systems and resource governance. The current text treats the indices primarily as financial assets. While this is methodologically necessary, the discussion should acknowledge that these indices represent firms critical to infrastructure development. High tail risk implies higher costs of capital, which can slow down the deployment of renewable technologies. The authors need to articulate how understanding extreme downside risk contributes to more stable investment environments, which is crucial for long-term planning in energy infrastructure.

Illustrate how your study aligns with existing research highlighting the transformative potential of sustainable practices, technological advancements, and efficient resource use in fostering economic development, social welfare, and global energy/environmental sustainability (doi: 10.1142/S263053482550007X; 10.1007/s43674-022-00045-9). By integrating these perspectives, the paper moves beyond a purely econometric exercise to become a policy-relevant contribution. The authors should discuss how their findings on moment existence (or lack thereof) impact the reliability of standard risk management tools used by pension funds and sovereign wealth funds that are increasingly mandated to invest in green assets. If higher moments are infinite, as suggested by some confidence intervals in the results, standard mean-variance optimization may fail, leading to suboptimal capital allocation that hinders the energy transition. This strategy situates your work within established academic conversations while underscoring its significance in tackling urgent challenges related to energy/environmental transitions, circular economy models, and fair resource allocation (doi: 10.1142/S1752890926500030; 10.1515/jafio-2017-0018). Specifically, the authors should revise the Introduction and Discussion sections to incorporate these themes. Explain how accurate tail risk measurement prevents market failures that could arise from mispriced risk in the renewable sector. Furthermore, discuss the implication of "finite variance but potentially infinite kurtosis" for regulatory stress testing. If regulators rely on models assuming finite fourth moments, they may underestimate the probability of extreme crashes in the renewable sector, leading to insufficient capital buffers. This connection between statistical properties and regulatory adequacy is currently underdeveloped and needs to be a central pillar of the revised manuscript.

While the use of LLRS with an optimal rank shift is a strong methodological choice, the manuscript requires a more detailed justification for the chosen truncation levels. The authors utilize 5% and 10% truncation levels, which are standard in Extreme Value Theory (EVT) literature. However, given the heterogeneity in the sample sizes across indices (ranging from roughly 5,000 to 5,176 observations), the effective number of tail observations varies. The authors should provide a more rigorous sensitivity analysis regarding the choice of the tail threshold. While Figure 2 shows stability across 2.5% to 15%, the discussion should explicitly address why the confidence intervals widen significantly at lower truncation levels (e.g., 5%) compared to 10%. Does this widening suggest that the power-law behavior is less robust in the extreme tail than in the semi-heavy tail? This distinction is critical for risk managers who are most concerned with the deepest tails (e.g., 99% VaR).

Furthermore, the robustness check using GARCH(1,1) filtered residuals (Appendix Table A2) yields notably different tail index estimates compared to raw returns. For instance, the tail indices for standardized residuals are generally higher, indicating thinner tails once volatility clustering is removed. The authors should discuss this discrepancy in the main text rather than relegating it to the appendix. Does this imply that the heavy-tailedness observed in raw returns is primarily driven by volatility clustering rather than inherent innovation distribution properties? If the innovations themselves have thinner tails, this has profound implications for modeling. A GARCH-EVT approach might be more appropriate than a static tail index estimation for forecasting future risk. The manuscript should clarify whether the heavy tails are due to conditional heteroskedasticity or unconditional distributional properties, as this dictates the appropriate risk modeling framework for practitioners. Additionally, the recursive expanding-window approach starts with an initial window of 500 observations. Given that the sample begins in 2005, this initial window ends around 2007, just prior to the Global Financial Crisis (GFC). The authors should discuss how sensitive the early recursive estimates are to this initialization point. Would starting with a larger initial window (e.g., 1,000 observations) alter the perceived magnitude of the tail risk spike during the GFC? The persistence of extreme events in an expanding window is noted, but the authors should also consider a rolling window approach in the discussion to differentiate between persistent structural breaks and temporary shock absorption. The current method inherently preserves the memory of past crises forever, which may overstate tail risk in calm periods. A comparative discussion on how a rolling window might show mean reversion in tail indices would strengthen the methodological critique and provide a more nuanced view of risk persistence.

The Discussion section currently summarizes the statistical findings well but falls short on actionable recommendations. The conclusion that renewable and conventional energy markets share similar tail risk profiles is vital, but the implications need to be unpacked. If renewable energy is not statistically safer in terms of tail risk, why should investors allocate capital there? The authors should address the risk-return trade-off more explicitly. Does the renewable sector offer a premium that compensates for this heavy-tailed risk? The literature review mentions mixed findings on performance; the discussion should reconcile these performance findings with the risk findings.

Moreover, the policy implications regarding "finite first and second moments" need elaboration. The authors state that Value at Risk (VaR) and Expected Shortfall (ES) are theoretically well-defined. However, in practice, estimation error for ES is known to be high even when moments exist. The authors should caution practitioners about the estimation risk involved in calculating ES for these indices, given the wide confidence intervals observed in Table 3. For policymakers, the finding that tail risk spikes during systemic crises (like the GFC) suggests that renewable energy stocks are not a safe haven during broad market downturns. This contradicts some narratives about green assets acting as hedges. The revised manuscript should explicitly discuss the diversification benefits (or lack thereof) during crisis periods based on the recursive analysis. If tail indices drop below 2 during crises, correlation structures likely change, potentially leading to contagion. This systemic risk aspect should be highlighted to warn regulators about the potential for synchronized failures in energy portfolios during macroeconomic shocks.

Minor Suggestions:

The data section mentions the sample ends in February 2025. Given the current date, this implies either a forecast or a very recent update. The authors must clarify the data source and availability. If this is projected data or if there is a typo in the year, it must be corrected immediately to maintain credibility. Assuming the data is accurate up to the most recent available date, please specify the exact cut-off date and ensure the Bloomberg terminal codes are provided for replicability. Additionally, the description of the indices in Table 1 is helpful, but the weighting schemes (equal-dollar vs. market-cap) should be discussed in the context of tail risk. Equal-weighted indices (like ECO) might exhibit different tail properties compared to cap-weighted indices (like SPGCE) due to exposure to smaller, potentially riskier firms. A brief comment on how index construction influences the tail index would be valuable.

Table 3 and Tables 4-5 are dense and difficult to read in their current format. The confidence intervals should be clearly separated from the point estimates, perhaps using brackets or distinct columns. The notation "Inconcl." in Appendix Table A4 should be defined in the table note explicitly as "Inconclusive" to avoid ambiguity. Figure 1 Panel B uses grey shaded areas for stress events; ensure these are clearly distinguishable in black-and-white printing, as many journals print in grayscale. Figure 3's y-axes are standardized, which is good for comparison, but the scale should be mentioned in the caption to prevent misinterpretation of the absolute levels of the tail indices.

Reviewer #4: The manuscript provides a comprehensive empirical analysis of heavy-tailedness in renewable and conventional energy equity indices, utilizing daily data from 2005 to 2025. The study’s primary contribution lies in its application of confidence-interval (CI)-based tail index inference, specifically using the log-log rank-size (LLRS) regression with an optimal rank shift of -1/2 to identify the existence of finite moments. While the topic is timely and the methodology is generally robust, several substantial issues must be addressed to justify publication. First, the introduction and literature review are excessively long and occasionally repetitive, particularly regarding the descriptions of the various renewable energy indices (ECO, SPGCE, ERIX, and SUN) . This background information should be more concisely integrated into the methodology or data section to improve the paper's flow. Second, while the study emphasizes that all examined indices possess finite first and second moments ($ζ > 2$) , the discussion on the potential infiniteness of third and fourth moments remains somewhat speculative. The authors should provide a more rigorous sensitivity analysis or additional statistical tests to solidify these claims, as they form the basis for the paper's warnings against skewness-based portfolio optimization. Third, the recursive analysis reveals a sharp decline in tail indices during the 2007-2008 Global Financial Crisis (GFC) , but the comparative analysis for the COVID-19 period is less detailed. Given the unique nature of the pandemic shock, a deeper exploration of why the tail risk response was "more modest" compared to the GFC would significantly enhance the discussion. Additionally, the manuscript would benefit from a clearer explanation of the economic drivers behind the observed similarities in tail behavior between renewable and conventional energy sectors, beyond just noting that they respond to systemic stress similarly. Finally, the formatting of the equations and the consistency of the notation throughout the paper need careful proofreading to ensure technical accuracy and readability. Addressing these points—conciseness in the literature review, more rigorous support for higher-order moment claims, deeper analysis of the COVID-19 period, and enhanced economic interpretation—is essential for the manuscript to meet the journal's standards.

**********

7. PLOS authors have the option to publish the peer review history of their article (what does this mean?). If published, this will include your full peer review and any attached files.

If you choose “no”, your identity will remain anonymous but your review may still be made public.

**Do you want your identity to be public for this peer review?**  For information about this choice, including consent withdrawal, please see our Privacy Policy.

Reviewer #1: No

Reviewer #3: No

Reviewer #4: No

**********

[NOTE: If reviewer comments were submitted as an attachment file, they will be attached to this email and accessible via the submission site. Please log into your account, locate the manuscript record, and check for the action link "View Attachments". If this link does not appear, there are no attachment files.]

To ensure your figures meet our technical requirements, please review our figure guidelines: https://journals.plos.org/plosone/s/figures

You may also use PLOS’s free figure tool, NAAS, to help you prepare publication quality figures: https://journals.plos.org/plosone/s/figures#loc-tools-for-figure-preparation.

NAAS will assess whether your figures meet our technical requirements by comparing each figure against our figure specifications.

# Author comment: Author response to Decision Letter 3

**DOI:** 10.1371/journal.pone.0351106.r006

16 May 2026

We are grateful to the Editors and the reviewers for very helpful comments and suggestions on the manuscript. The attached Response to Reviewers file contain the description of the changes made in the paper following them.

Attachment Submitted filename: PONE-D-24-59227_Response to Editor and Reviewers (Rev3).docx

# Peer review: Decision Letter 3

**DOI:** 10.1371/journal.pone.0351106.r007

**Authors:** Kyungwon Kim

24 May 2026

Tail Risk, Large Fluctuations and Downfalls in Renewable Energy Markets

PONE-D-24-59227R3

Dear Dr. Ibragimov,

We’re pleased to inform you that your manuscript has been judged scientifically suitable for publication and will be formally accepted for publication once it meets all outstanding technical requirements.

Within one week, you’ll receive an e-mail detailing the required amendments. When these have been addressed, you’ll receive a formal acceptance letter and your manuscript will be scheduled for publication.

An invoice will be generated when your article is formally accepted. Please note, if your institution has a publishing partnership with PLOS and your article meets the relevant criteria, all or part of your publication costs will be covered. Please make sure your user information is up-to-date by logging into Editorial Manager at Editorial Manager® and clicking the ‘Update My Information' link at the top of the page. For questions related to billing, please contact billing support.

If your institution or institutions have a press office, please notify them about your upcoming paper to help maximize its impact. If they’ll be preparing press materials, please inform our press team as soon as possible -- no later than 48 hours after receiving the formal acceptance. Your manuscript will remain under strict press embargo until 2 pm Eastern Time on the date of publication. For more information, please contact onepress@plos.org.

Kind regards,

Kyungwon Kim

Academic Editor

PLOS One

Additional Editor Comments (optional):

Dear Authors,

Congratulations on the acceptance of your manuscript! I would like to personally commend you for your persistence and the rigorous revisions undertaken over multiple review rounds. Both reviewers have indicated that you have comprehensively addressed all outstanding concerns, resulting in a highly robust and timely analysis of tail risks in renewable energy markets.

Thank you for choosing to publish your valuable research with PLOS ONE. I wish you continued success in your future academic endeavors.

Reviewers' comments:

Reviewer's Responses to Questions

**Comments to the Author**

1. If the authors have adequately addressed your comments raised in a previous round of review and you feel that this manuscript is now acceptable for publication, you may indicate that here to bypass the “Comments to the Author” section, enter your conflict of interest statement in the “Confidential to Editor” section, and submit your "Accept" recommendation.

Reviewer #1: All comments have been addressed

Reviewer #3: (No Response)

**********

2. Is the manuscript technically sound, and do the data support the conclusions?

The manuscript must describe a technically sound piece of scientific research with data that supports the conclusions. Experiments must have been conducted rigorously, with appropriate controls, replication, and sample sizes. The conclusions must be drawn appropriately based on the data presented.

Reviewer #1: Yes

Reviewer #3: (No Response)

**********

3. Has the statistical analysis been performed appropriately and rigorously?

Reviewer #1: Yes

Reviewer #3: (No Response)

**********

4. Have the authors made all data underlying the findings in their manuscript fully available?

The PLOS Data policy requires authors to make all data underlying the findings described in their manuscript fully available without restriction, with rare exception (please refer to the Data Availability Statement in the manuscript PDF file). The data should be provided as part of the manuscript or its supporting information, or deposited to a public repository. For example, in addition to summary statistics, the data points behind means, medians and variance measures should be available. If there are restrictions on publicly sharing data—e.g. participant privacy or use of data from a third party—those must be specified.

Reviewer #1: Yes

Reviewer #3: (No Response)

**********

5. Is the manuscript presented in an intelligible fashion and written in standard English?

PLOS ONE does not copyedit accepted manuscripts, so the language in submitted articles must be clear, correct, and unambiguous. Any typographical or grammatical errors should be corrected at revision, so please note any specific errors here.

Reviewer #1: Yes

Reviewer #3: (No Response)

**********

6. Review Comments to the Author

Please use the space provided to explain your answers to the questions above. You may also include additional comments for the author, including concerns about dual publication, research ethics, or publication ethics. (Please upload your review as an attachment if it exceeds 20,000 characters)

Reviewer #1: Dear Authors,

Thank you for your detailed revision of the manuscript.

You have addressed all comments comprehensively.

All the best!

Reviewer #3: recommend acceptance

recommend acceptance

recommend acceptance

recommend acceptance

recommend acceptance

recommend acceptance

**********

7. PLOS authors have the option to publish the peer review history of their article (what does this mean?). If published, this will include your full peer review and any attached files.

If you choose “no”, your identity will remain anonymous but your review may still be made public.

**Do you want your identity to be public for this peer review?**  For information about this choice, including consent withdrawal, please see our Privacy Policy.

Reviewer #1: No

Reviewer #3: No

**********

# Editor report: Acceptance letter

**DOI:** 10.1371/journal.pone.0351106.r008

**Authors:** Kyungwon Kim

PONE-D-24-59227R3

PLOS One

Dear Dr. Ibragimov,

I'm pleased to inform you that your manuscript has been deemed suitable for publication in PLOS One. Congratulations! Your manuscript is now being handed over to our production team.

At this stage, our production department will prepare your paper for publication. This includes ensuring the following:

* All references, tables, and figures are properly cited

* All relevant supporting information is included in the manuscript submission,

* There are no issues that prevent the paper from being properly typeset

You will receive further instructions from the production team, including instructions on how to review your proof when it is ready. Please keep in mind that we are working through a large volume of accepted articles, so please give us a few days to review your paper and let you know the next and final steps.

Lastly, if your institution or institutions have a press office, please let them know about your upcoming paper now to help maximize its impact. If they'll be preparing press materials, please inform our press team within the next 48 hours. Your manuscript will remain under strict press embargo until 2 pm Eastern Time on the date of publication. For more information, please contact onepress@plos.org.

You will receive an invoice from PLOS for your publication fee after your manuscript has reached the completed accept phase. If you receive an email requesting payment before acceptance or for any other service, this may be a phishing scheme. Learn how to identify phishing emails and protect your accounts at https://explore.plos.org/phishing.

If we can help with anything else, please email us at customercare@plos.org.

Thank you for submitting your work to PLOS ONE and supporting open access.

Kind regards,

PLOS ONE Editorial Office Staff

on behalf of

Professor Kyungwon Kim

Academic Editor

PLOS One
